# Ixabepilone Administered Weekly or Every Three Weeks in HER2-Negative Metastatic Breast Cancer Patients; A Randomized Non-Comparative Phase II Trial

**DOI:** 10.1371/journal.pone.0069256

**Published:** 2013-07-23

**Authors:** George Fountzilas, Vassiliki Kotoula, Dimitrios Pectasides, George Kouvatseas, Eleni Timotheadou, Mattheos Bobos, Xanthipi Mavropoulou, Christos Papadimitriou, Eleni Vrettou, Georgia Raptou, Angelos Koutras, Evangelia Razis, Dimitrios Bafaloukos, Epaminontas Samantas, George Pentheroudakis, Dimosthenis V. Skarlos

**Affiliations:** 1 Department of Medical Oncology, “Papageorgiou” Hospital, Aristotle University of Thessaloniki School of Medicine, Thessaloniki, Greece; 2 Department of Pathology, Aristotle University of Thessaloniki School of Medicine, Thessaloniki, Greece; 3 Oncology Section, Second Department of Internal Medicine, “Hippokration” Hospital, Athens, Greece; 4 Health Data Specialists Ltd, Athens, Greece; 5 Laboratory of Molecular Oncology, Hellenic Foundation for Cancer Research, Aristotle University of Thessaloniki School of Medicine, Thessaloniki, Greece; 6 Department of Radiology, “AHEPA” Hospital, Aristotle University of Thessaloniki School of Medicine, Thessaloniki, Greece; 7 Department of Clinical Therapeutics, “Alexandra” Hospital, University of Athens School of Medicine, Athens, Greece; 8 Division of Oncology, Department of Medicine, University Hospital, University of Patras Medical School, Patras, Greece; 9 Third Department of Medical Oncology, “Hygeia” Hospital, Athens, Greece; 10 First Department of Medical Oncology, “Metropolitan” Hospital, Piraeus, Greece; 11 Third Department of Medical Oncology, “Agii Anargiri” Cancer Hospital, Athens, Greece; 12 Department of Medical Oncology, Ioannina University Hospital, Ioannina, Greece; 13 Second Department of Medical Oncology, “Metropolitan” Hospital, Piraeus, Greece; Institute for Medical Biomathematics, Israel

## Abstract

To explore the activity and safety of two schedules of ixabepilone, as first line chemotherapy, in patients with metastatic breast cancer previously treated with adjuvant chemotherapy, a randomized non-comparative phase II study was conducted. From November 2008 until December 2010, 64 patients were treated with either ixabepilone 40 mg/m^2^ every 3 weeks (Group A, 32 patients) or ixabepilone 20 mg/m^2^ on days 1, 8 and 15 every 4 weeks (Group B, 32 patients). Overall response rate (the primary end point) was 47% in Group A and 50% in Group B. The most frequent severe adverse events were neutropenia (32% vs. 23%), metabolic disturbances (29% vs. 27%) and sensory neuropathy (12% vs. 27%). Two patients in Group A and 3 in Group B developed febrile neutropenia. After a median follow-up of 22.7 months, median progression-free survival (PFS) was 9 months in Group A and 12 months in Group B. Median survival was 26 months in Group A, whereas it was not reached in Group B. Multiple genetic and molecular markers were examined in tumor and peripheral blood DNA, but none of them was associated with ORR or drug toxicity. Favorable prognostic markers included: the T-variants of ABCB1 SNPs c.2677G/A/T, c.1236C/T and c.3435C/T, as well as high MAPT mRNA and Tau protein expression, which were all associated with the ER/PgR-positive phenotype; absence of TopoIIa; and, an interaction between low TUBB3 mRNA expression and Group B. Upon multivariate analysis, tumor ER-positivity was a favorable (p = 0.0092) and TopoIIa an unfavorable (p = 0.002) prognostic factor for PFS; PgR-positivity was favorable (p = 0.028) for survival. In conclusion, ixabepilone had a manageable safety profile in both the 3-weekly and weekly schedules. A number of markers identified in the present trial appear to deserve further evaluation for their prognostic and/or predictive value in larger multi-arm studies.

**Trial Registration:**

ClinicalTrials.gov NCT 00790894

## Introduction

Breast cancer is the most common malignant neoplasm and the second leading cause of death from cancer in women both in the USA and Europe [1], [2]. It is established that both adjuvant chemotherapy and hormonal therapy prolong disease-free and overall survival [3]. Anthracyclines and taxanes are the two most commonly used classes of agents in this setting. However, despite the optimal management of patients with early-stage breast cancer, eventually approximately 30% of them suffer from disease relapse [4].

Metastatic breast cancer (MBC) is an incurable disease with few therapeutic options. With the increasing use of anthracyclines and taxanes in the adjuvant setting the number of available drugs for these patients is even more limited. Obviously, there is an unmet need for the introduction to the clinic of agents with novel modes of action, lack of cross-resistance with existing agents and promising activity in metastatic breast cancer.

Epothilones comprise a novel class of chemotherapeutic drugs, which, like paclitaxel, are stabilizing microtubules and cause cell cycle arrest [5] and subsequent apoptosis. Ixabepilone (Bristol-Myers Squibb, BMS-247550) is a semisynthetic analogue of epothilone B, in which the lactone oxygen of epothilone B is replaced by nitrogen to increase drug stability [6].

In vitro studies have shown that ixabepilone is active in cancer cells with upregulated βIII-tubulin expression, which is actually linked with resistance to taxanes and vinca alkaloids [7]–[9]. Furthermore, in preclinical models the drug was found to be a poor substrate for multidrug resistance (MDR) and does not strongly induce P-glycoprotein expression [10], [11]. Its low susceptibility to multiple mechanisms of drug resistance and the lack of cross-resistance with commonly used agents, such as taxanes [12], [13], make ixabepilone an attractive potential candidate for the treatment of breast cancer previously exposed to taxanes and anthracyclines. In support of this notion, a phase III trial clearly demonstrated that the combination of ixabepilone and capecitabine was more efficacious than capecitabine monotherapy in patients with MBC resistant to anthracyclines and taxanes [14]. Currently, ixabepilone is the only epothilone to receive approval by the United States Food and Drug Administration (FDA) and other Regulatory Agencies for the treatment of MBC.

Ixabepilone has been extensively studied in MBC, both in chemotherapy-naive and in heavily pretreated patients (reviewed in ref. [11], [15], [16]). In most phase II or randomized trials, reported so far, ixabepilone was administered at a dose of 40 mg/m^2^ as a 3-hour infusion every 3 weeks. In an attempt to repeat the success story of weekly paclitaxel in the treatment of MBC, as demonstrated in large phase III trials [17], [18], investigators are currently evaluating the efficacy and safety profile of weekly ixabepilone.

To further explore the clinical profile of weekly administration of ixabepilone, the Hellenic Cooperative Oncology Group (HeCOG) designed a randomized non-comparative phase II trial (HE11A/08) in patients with MBC, previously treated with adjuvant chemotherapy, evaluating the approved dose of ixabepilone monotherapy and that of 20 mg/m^2^ on days 1, 8 and 15 of a 4-week cycle, as first line chemotherapy. The primary objective of the study was overall response rate (ORR). Secondary objectives were safety profile, progression-free survival (PFS), duration of overall response, time to treatment failure (TTF), time to tumor progression (TTP) and survival. We report here the final analysis of this study. Results of a pre-planned collateral translational research study exploring the predictive/prognostic role of certain biological markers are also reported.

## Patients and Methods

### Eligibility

Eligibility criteria were biopsy-proven diagnosis of HER2-negative, locally recurrent or MBC, age ≥18 years, Eastern Cooperative Oncology Group (ECOG) performance status (PS) of 0 or 1, life expectancy of at least 12 weeks, adequate hematological, hepatic and renal function, measurable disease by RECIST criteria and history of adjuvant chemotherapy containing an anthracycline or a taxane, with no previous chemotherapy treatment in the metastatic setting. In July 2009 the protocol was amended and patients were allowed to enter the study independently of the type of adjuvant chemotherapy. Detailed eligibility criteria are provided as supporting information (Supporting Information S1).

All patients were required to sign a study-specific informed consent before randomization. A separate informed consent was also signed, allowing the acquisition of biological material by the investigator for future research studies. The protocol was approved by the Institutional Review Board in each participating Institution and by the National Organization for Medicines. Ixabepilone was provided free of charge by Bristol-Myers Squibb (Princeton, NJ). The protocol for this trial and supporting CONSORT checklist are available as supporting information; see Checklist S1 and Protocol S1.

### Treatment

Stratified block randomization balanced by center was performed centrally, at the HeCOG Data Office in Athens. Stratification factors included time to recurrence from the date of the last dose of adjuvant chemotherapy to the date of recurrence (≤1 year vs. >1 year) and following the amendment of the protocol, history of taxane-containing adjuvant chemotherapy (yes vs. no).

Patients were randomized to receive, as first line chemotherapy, either the approved dose of ixabepilone of 40 mg/m^2^ over a 3-hour infusion on day 1 of a 3-week cycle (Group A) or ixabepilone 20 mg/m^2^ over a 3-hour infusion weekly for three weeks followed by one week off (Group B), as recommended in a phase I trial by Dickson N et al [19] available at the time of the design of the study. Prior to drug infusion all patients received hypersensitivity prophylaxis with an H_3_-antagonist, dimetindene maleate and ranitidine intravenously. Premedication with corticosteroids was not routinely administered. However, oral corticosteroids were required 12 hours prior to the ixabepilone infusion for all patients who had experienced a hypersensitivity reaction (HSR) in any previous cycle. Antiemetics were not routinely administered but were added to the regimen in patients who experienced severe nausea or vomiting. Granulocyte-colony stimulating factor (G-CSF) was not given initially during cycle 1, but could be added to subsequent treatment cycles in patients with febrile neutropenia or delayed neutrophil recovery requiring a dose delay. Treatment was continued until disease progression, intolerable toxicity or consent withdrawal by the patient. Dose adjustments for adverse reactions are shown as supporting information (Supporting Information S1). In case toxicities recurred after the initial dose reduction, an additional 20% dose reduction was recommended. If toxicities recurred after the second dose reduction, administration of ixabepilone was discontinued permanently. In case bisphophonates had to be given concurrently with the ixabepilone treatment, they were administered sequentially following the infusion of the drug.

**Figure 1:**
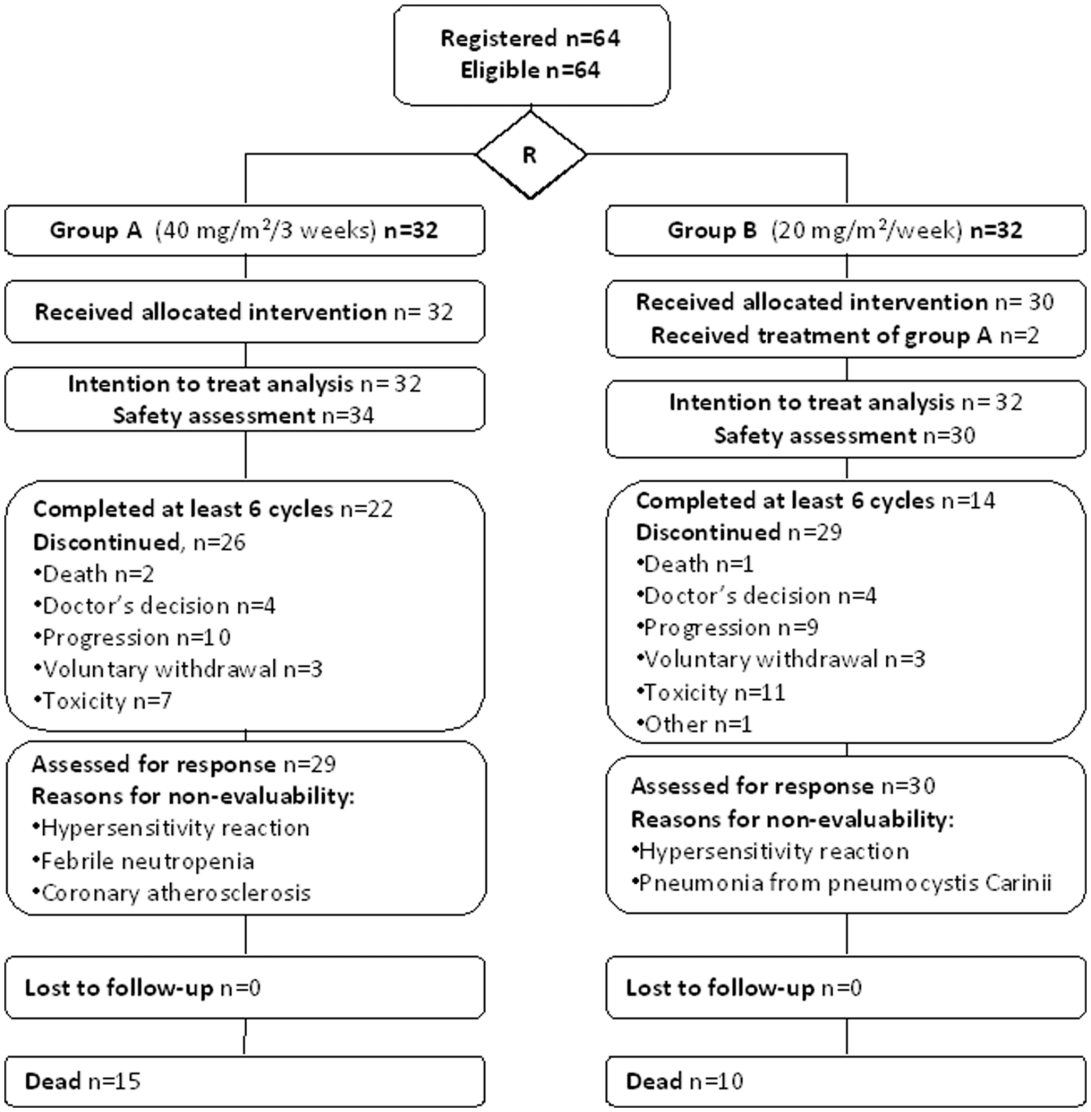
Consort diagram describing the main characteristics of the present clinical study.

Data entry was performed in a central data-base by trained HeCOG data managers located in the different participating centers. The study was internally monitored by certified HeCOG personnel. Patients were examined at the Clinic every three months following the discontinuation of the treatment with ixabepilone. All imaging material pertinent to treatment response was assessed centrally by one of the authors (X.M.) after the completion of the study. The consort diagram of the study is shown in Figure 1.

### Patient Samples and Translational Biomarker Assessments

A total of 62 formalin-fixed paraffin-embedded (FFPE) tumor tissue blocks and 62 peripheral blood samples were obtained before the initiation of the treatment for the investigation of genetic/molecular markers, which was accomplished at the Laboratory of Molecular Oncology of the Hellenic Foundation of Cancer Research/Aristotle University of Thessaloniki. FFPE sections were histologically evaluated for tumor presence and cell content. Tissue microarray (TMA) blocks were constructed using a manual arrayer (Model I, Beecher Instruments, Sun Prairie, WI), including two 1.5 mm cores per tumor and multiple neoplastic and non-neoplastic tissue samples as controls for slide-based assays.

#### Immunohistochemistry (IHC)

IHC was applied on serial 2.5 µm thick TMA sections for the following proteins: estrogen receptor alpha (ER), progesterone receptor (PgR), HER2, Ki67, tau protein, βΙΙΙ-tubulin, γ-tubulin, topoisomerase II alpha (TopoIIa) and MLH-1, MSH-2, MSH-6 and PMS-2 (mismatch repair [MMR] proteins). Tau protein was selected as a marker associated with response to paclitaxel [20]; tubulins as epothilone targets [5]; and MMR proteins in order to observe the microsatellite stability status of the primary tumors before patients received any chemotherapy [21]. Method details and evaluation criteria are presented as supporting information (Supporting Information S2).

#### Fluorescence in situ hybridization (FISH)

FISH was applied on 6 µm thick TMA sections using the ZytoLight® SPEC HER2/TOP2A/CEN17 triple color probe (ZytoVision, Bremerhaven, Germany), as previously described [22]. Evaluation criteria are described in the supporting information (Supporting Information S2). Overall, HER2 was considered to be positive if the gene was amplified (ratio >2.2 or copy number >6) by FISH and/or a HER2 score of 3+ was obtained by IHC.

#### Single nucleotide polymorphism (SNP) assessment

Missense SNPs previously associated with taxanes toxicity (http://www.pharmgkb.org) were examined on DNA from peripheral blood samples: ABCB1 gene, rs2032582 (c.2677T/G/A, p.S893T/A), rs1128503 (c.1236T/C, p.G412G) and rs1045642 (c. 3435T/C, p.I1145I); CYP3A4 gene, rs12721627 (c.20716C/G, p.T185S); and, CYP2C8 gene, rs11572080 (c.7225G/A, p.R69K). Details on SNP selection and sequencing assays can be found in the supporting information (Supporting Information S2).

#### mRNA expression studies

Tumor tissue RNA was extracted from TMA cores with TRIZOL-LS and reverse transcribed with Superscript III (all from Invitrogen, Life technologies, Paisley, UK). Relative mRNA expression was assessed with qPCR by using premade exon-spanning TaqMan® Gene Expression Assays (Applied Biosystems/Life Technologies) for ABCB1, CYP2C8, CYP3A4, MAPT (microtubule-associated protein tau) and TUBB3 (tubulin β-3). Samples were run in duplicates in an ABI7900HT real time PCR system. Target mRNA expression was normalized against GUSB, which was used as the endogenous reference [23]. Relative quantification (RQ) was assessed in a linear mode as (40– dCT) [24], whereby dCT = (avg CT target) – (avg CT GUSB). Details on RNA extraction, qPCR results evaluation and assay description can be found in the supporting information (Supporting Information S2).

### Statistical Analysis

In this multicenter phase II randomized, open-label, non-comparative trial (parallel assignment and efficacy study) the primary objective on an intent-to-treat analysis was ORR (by RECIST criteria). With a fixed sample size of 150 patients (75 patients in each treatment arm), a 95% confidence level and an expected objective response rate around 30%, the maximum width of the confidence interval (using the normal approximation) for ORR would be ±10% (i.e. 20%). The study was closed prematurely due to the low rate of accrual. Regarding the definition of other end points reported in this study: Time to best overall response was calculated as the time from random assignment until the measurement criteria were met for complete or partial response (best of the two). Duration of best overall response was calculated as the time from when the measurement criteria were met for complete or partial response (best of the two) until disease progression or death from any cause. TTF was calculated as the time from random assignment to disease progression or death from any cause or early treatment discontinuation. TTP was calculated as the time from random assignment to documentation of disease progression. PFS was calculated as the time from random assignment to disease progression or death from any cause. Survival was calculated as the time from random assignment to death from any cause. Event-free patients at last contact were censored (for all time to event variables). Safety profile was reported according to the treatment actually received.

Biomarkers (germline SNPs, proteins and relative mRNA expression) were associated with each other and were explored in relation to ORR, PFS and survival. SNPs were evaluated for homozygous vs. heterozygous alleles (3-scale variables) and for one allele vs. the other (binary variables). RQ values for mRNA markers were examined as continuous variables and as categorical ones with cutoffs at the 1^st^, 2^nd^ (median RQ value) and 3^rd^ quartiles. IHC assessed biomarkers included the following: ER and PgR (positive vs. negative); Ki67 (<14% vs. ≥14%); tau protein and βΙΙΙ-tubulin (intensity 0–1 vs. 2–3); γ-tubulin (intensity 1 and 2 vs. 3); and, TopoIIa (intensity 2 or 3 in >5% of the tumor cells vs. all other).

Time to event curves were estimated by the Kaplan-Meier method and compared with the log-rank test, while the Fisher’s exact test was used for all univariate tests of categorical variables. The predictive value of the biomarkers on PFS and survival was evaluated in an unplanned exploratory univariate analysis, with an interaction test between group and the biomarker in a Cox's proportional hazards model. All tests were two sided at alpha = 5%.

The prognostic effect of the various factors that were found statistically significant in univariate analysis on PFS and survival was tested in a multivariate Cox's proportional hazards model using the Wald chi square for estimating p-values. These were: treatment group, number of metastatic sites (1–3 vs. >3), visceral metastasis (yes vs. no), ER (positive vs. negative), PgR (positive vs. negative), Ki67 (<14% vs. ≥14%), dichotomized MAPT mRNA (high vs. low at the 2^nd^ quartile), dichotomized TUBB3 mRNA (high vs. low at the 3^rd^ quartile), tau protein (intensity 0–1 vs. 2–3), ABCB1 3435 (T or T/C vs. C), ABCB1 1236 (T or T/C vs. C), ABCB1 2677 (G or G/A vs. T or T/G) and TopoIIa (positive vs. negative). The process to identify the best subset of prognostic factors was not only based on a backward selection process but also involved factor exclusion in subsequent steps. This applied for factors with a large number of missing values and/or with coefficients demonstrating high variability between subsequent steps. This methodology was followed in order to assess the stability of the model findings, since the size of the study sample was smaller than initially planned. Overall, given the small number of patients and large number of markers, p-values are presented as “descriptive statistics” and should be used as guidelines and not formal definitive probabilities. Results of this study were presented according to reporting recommendations for tumor marker prognostic studies [25].

## Results

### Patient Characteristics and Compliance

Selected important patient and tumor characteristics are depicted in Tables 1 and 2, respectively. In total, 64 patients (32 in each group) were randomized from November 2008 until December 2010. Two patients were treated as in Group A even though they were randomized to Group B (protocol violators). These patients were analyzed for response and survival as allocated and for safety as treated. Most of the patients were postmenopausal with a median PS of 0. None of the patients received prior chemotherapy for advanced disease, however all 64 patients had been treated with chemotherapy in the adjuvant setting (Table 1). Visceral metastases were recorded in 26 (81%) of the patients in Group A and 27 (84%) in Group B (Table 2).

**Table 1:** Selected patient characteristics.

	Group A(3-weekly)	Group B(weekly)
Age (years)		
Median	53.4	60.65
Range	32–75	31–74
	N (%)	N (%)
PS		
0	26 (81)	27 (84)
1	6 (19)	5 (16)
Menopausal status		
Premenopausal	11 (34)	5 (16)
Postmenopausal	21 (66)	27 (84)
Adjuvant CT		
Anthracycline-containing	26 (81)	27 (84)
Taxane-containing	20 (63)	26 (81)
Adjuvant HT		
Yes	28 (88)	28 (88)
No	4 (13)	4 (13)
Adjuvant RT		
Yes	26 (81)	24 (75)
No	6 (19)	8 (25)
RFI (months)		
≤12	4 (13)	4 (13)
>12	28 (88)	28 (88)
HT for advanced disease		
Yes	0 (0)	6 (19)
No	31 (97)	26 (81)
Unknown	1 (3)	0 (0)

PS = performance status; CT = chemotherapy; HT = hormonal therapy; RT = radiation therapy; RFI = relapse-free interval.

**Table 2:** Selected tumor characteristics.

	Group A(3-weekly)	Group B(weekly)
	N (%)	N (%)
ER status (n = 62)		
Positive	29 (91)	24 (80)
Negative	3 (9)	6 (20)
PgR status (n = 60)		
Positive	24 (75)	17 (61)
Negative	8 (25)	11 (39)
HER2 (n = 61)		
Positive	5 (16)	2 (7)
Negative	26 (84)	28 (93)
Ki67 (n = 62)		
Positive (High: ≥14%)	24 (75)	22 (73)
Negative (Low: <14%)	8 (25)	8 (27)
Histological grade		
1	2 (6)	2 (6)
2	13 (41)	17 (53)
3	15 (47)	12 (38)
Unknown	2 (6)	1 (3)
Triple-negative patients (n = 59; A = 31 and B = 28)	1 (3)	5 (18)
Metastatic sites		
Locoregional	14 (44)	9 (28)
Nodes	17 (53)	9 (28)
Skin	3 (9)	0 (0)
Residual breast	2 (6)	0 (0)
Soft tissue	4 (12)	3 (9)
Distant	31 (97)	32 (100)
Bones	14 (44)	18 (56)
Lung/pleura	22 (69)	22 (69)
Liver	17 (53)	16 (50)
Brain	1 (3)	1 (3)
Soft tissue	17 (56)	23 (72)
Other breast	0 (0)	1 (3)
Visceral metastases	26 (81.3%)	27 (84.4%)
Multiple metastases		
1–3	24 (75)	23 (72)
>3	8 (25)	9 (28)

Twenty-two patients (69%) completed at least six cycles in Group A and 14 (44%) in Group B (Table 3). Eighteen patients from Group A continued ixabepilone beyond the sixth cycle (range of cycles 1–9). In Group B, no patient continued treatment beyond the 9^th^ cycle. Treatment data for all patients are presented in Table 3.

**Table 3:** Treatment Characteristics.

	Group A(3-weekly) (N = 32)	Group B(weekly) (N = 32)
Number of cycles	212	150
% of cycles at full dosage	44	40
% of cycles with a delay^a^	9	21
Number of cycles	N	N
1	32	32
2	30	30
3	28	25
4	25	23
5	25	16
6	22	14
7	18	5
8	12	4
9	7	1
10	4	0
11	2	0
12	2	0
13	2	0
14	2	0
15	1	0
Median number of cycles	7	5
N with treatment discontinuation^b^	14	27
Cumulative dose^b^ (mg/m^2^)		
Median	230.6	240.0
Range	39.2–240.7	21.4–360.0
DI^b^ of ixabepilone		
Planned	13.3	15.0
Median delivered	13.1	11.1
Range	7.2–13.6	4.3–13.9
Relative DI		
Median delivered	0.98	0.74
Range	0.5–1.0	0.3–0.9

N = number of patients, DI = dose intensity (mg/m^2^/week).

a >2 days.

b Estimated at the 6^th^ cycle.

Twenty-six (81%) of the patients in Group A and 29 (91%) in Group B discontinued treatment before the completion of the study. Three patients died while on treatment. The first one, a 41-year old woman in Group A (treated in A but randomized in B) died from pneumocystis carinii pneumonia (PCP) a few days after the first cycle (the cause of death was not treatment-related according to the investigator but possibly treatment-related according to HeCOG). The second one, a 68-year old patient in Group A, with a history of ischemic heart disease died suddenly after the first cycle. Autopsy revealed extensive coronary atherosclerosis. The cause of death was therefore considered to be non-treatment related. The third one, a 57-year old patient in Group A, died from sepsis associated with febrile neutropenia and diarrhea after the completion of the third cycle. Other reasons for treatment discontinuation included tumor progression (10 patients in Group A and 9 patients in Group B), voluntary withdrawal (3 patients in each group) and adverse events (7 patients in Group A, 5 due to neurotoxicity, one due to musculoskeletal pain and one due to an allergic reaction and 11 patients in Group B, 10 due to neurotoxicity and one due to a hypersensitivity reaction).

Maintenance hormonal therapy was given to 5 patients of Group A and 9 of Group B. Further, chemotherapy following progression of disease was given to 16 patients of Group A and 12 of Group B.

### Response and Safety

In total, 59 patients (29 vs. 30 in Groups A and B, respectively) were evaluable for response. Reasons for non-evaluable response included early death of three patients, as previously stated, and severe HSR in two patients (one in each Group). Of note, central assessment of response was performed in 55 patients. For the rest of the patients imaging material was not available for central review.

**Figure 2:**
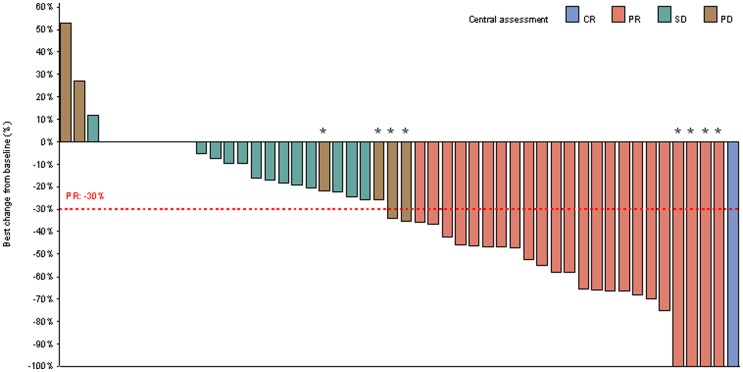
Waterfall plot of best change in target-lesion size from baseline, assessed by central review. In four patients (marked with a star), the best change in total lesion size from baseline showed an overall decrease but they were characterized as having progressive disease (PD) due to the development of new lesions. In four other patients (marked with a star as well), the best change in total lesion size from baseline was 100% but they were identified as partial response (PR) since in non-target lesions the response was stable disease (SD). CR: complete response.

Best response data as given by local investigators or following central review are given in Table 4, while in Figure 2 (Waterfall), best change in target-lesion size from baseline is shown, as assessed by central review. Locally assessed ORR in Group A and Group B was 47% and 50%, respectively, while following central review, it was 44% and 45%, respectively (Table 4). Median time interval to achieve best overall response (locally-assessed) was 9 weeks (95% confidence interval [CI] 8–16; range 8–30, N = 15) in Group A and 9 weeks (95% CI 8–16; range 7–18, N = 16) in Group B. Respectively, following central review, it was 18 weeks (95% CI 9–22; range 8–37, N = 13) in Group A and 17 (95% CI 16–25; range 8–26, N = 14) in Group B, a result of a number of responses seen in early imaging material by local investigators not being verified by central review. Notably, improvement of response (locally-assessed) was not observed in any patient while on the study beyond the 6^th^ cycle of chemotherapy.

**Table 4:** Best response as given by local investigators (n = 59).

	Group A (3-weekly)			Group B (weekly)		
	N	%	95% CI	N	%	95% CI
Patients reviewed	32 (29)			32 (31)		
NE^a^	3 (3)	9 (10)	2–25	2 (2)	6 (6)	1–21
CR	1 (1)	3 (3)	0.1–16	2 (0)	6 (0)	0.8–21
PR	14 (12)	44 (41)	26–62	14 (14)	44 (45)	26–62
SD	10 (10)	31 (34)	16–50	10 (9)	31 (29)	16–50
PD	4 (3)	13 (10)	4–29	4 (6)	13 (19)	4–29
ORR	15 (13)	47 (44)	29–65	16 (14)	50 (45)	32–68
Clinical benefit^b^	20 (17)	63 (59)	44–79	21 (17)	66 (55)	47–81

CR = complete response; PR = partial response; ORR = overall response rate; SD = stable disease; PD = progressive disease; NE = non-evaluable.

% values were rounded up.

a For details see text.

b CR+PR+SD for at least 24 weeks.

c Four patients were not centrally assessed.

(Numbers in parentheses indicate best response by central review [N = 55^c^]).

Median duration of overall response assessed locally was 34 weeks (95% CI 13–53; range 11–100) in Group A and 87 weeks (95% CI 22-not reached; range 15–134) in Group B. Respectively, following central review, it was 35 weeks (95% CI 13–63; range 11–101) in Group A and 87 weeks (95% CI 27-not reached yet; range 3–143+) in Group B.

The incidence of severe adverse events (severity grade 3–5) is shown in Table 5. The most frequent severe adverse events (Group A vs. Group B) were neutropenia (32% vs. 23%), metabolic disturbances (29% vs. 27%) and sensory neuropathy (12% vs. 27%). Median time to onset of peripheral neuropathy was 2 months in both groups, while median duration was 3 months in Group A and 2.5 months in Group B including 65% of events in Group A and 74% in Group B, which were still unresolved 30 days after treatment discontinuation, the time point at which adverse events’ follow-up, as part of the study, ended. All adverse events of any grade are shown as supporting information (Table S1). One patient in Group A died from sepsis after the third cycle, associated with febrile neutropenia, as previously stated. Five patients (2 vs. 3) developed febrile neutropenia. Twelve patients in total, 7 (22%) in Group A and 5 (16%) in Group B received G-CSF.

**Table 5:** Number of patients with severe adverse events.

	Group A (N = 34)			Group B (N = 30)		
	(3-weekly)			(weekly)		
	Grade			Grade		
	3	4	5	3	4	5
Allergic reaction	2 (6%)			1 (3%)		
Anemia		1 (3%)		1 (3%)		
Cardiac ischemia/infarction	1 (3%)		1 (3%)			
Coagulation				1 (3%)		
Constipation	1 (3%)					
Diarrhea	1 (3%)			1 (3%)		
Fatigue	3 (9%)			5 (17%)		
Febrile neutropenia	1 (3%)		1* (3%)	3 (10%)		
Hypotension		1 (3%)				
Infection			1 (3%)	7 (23%)		
Insomnia	1 (3%)					
Leukopenia	5 (15%)	1 (3%)		7 (23%)	1 (3%)	
Metabolic/Laboratory†	9 (26%)	1 (3%)		8 (27%)		
Motor neuropathy	2 (6%)			1 (3%)		
Mucositis	1 (3%)					
Musculoskeletal				1 (3%)		
Nail changes	1 (3%)			1 (3%)		
Neutropenia	10 (29%)	1 (3%)		4 (13%)	3 (10%)	
Pain	3 (9%)			1 (3%)		
Pulmonary‡	2 (6%)	1 (3%)			1 (3%)	
Renal failure	1 (3%)					
Sensory neuropathy	4 (12%)			8 (27%)		
Vomiting	2 (6%)					
Weight gain				1 (3%)		

† Included hyperkalemia, hypermagnesemia, hypokalemia, hyponatremia, hypophosphatemia and increased levels of ALT, GGT, CPK, LDH, urea and bilirubin.

‡ Included cough, dyspnea and acute pulmonary edema.

* Died from sepsis.

### Survival

**Figure 3:**
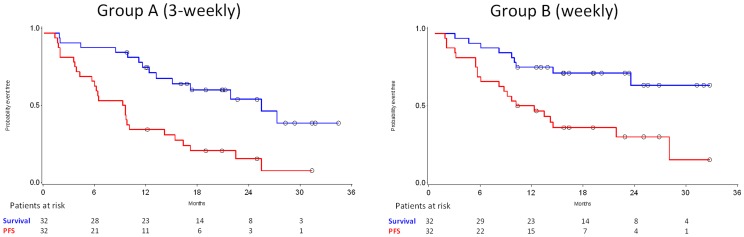
PFS and survival for Groups A and B (red lines: PFS; blue lines: survival).

After a median follow-up of 22.7 months for Group A patients and 20.2 months for Group B, 39 (61%) of the patients demonstrated tumor progression (23 vs. 16) and 25 (39%) died (15 vs. 10). Median PFS in Group A was 9 months (95% CI 4–14) and in Group B 12 months (95% CI 6–28). Respectively, median survival in Group A was 26 months (95% CI 13-not reached), whereas it was not reached in Group B (95% 24-not reached) (Figure 3). Data (median, 95% CI and range) on TTP and TTF are given as supporting information (Table S2).

### Biomarker Associations

Samples available for the DNA, RNA and IHC studies and the method performance for each marker are shown in the REMARK diagram as supporting information (Figure S1). SNP and IHC results are shown as supporting information in Tables S3 and S4, respectively, while mRNA RQ values are described in Tables S5 and S6. The number of samples was too small to create groups with SNP combinations. The majority of the patients were homozygous for the ancestral C-allele for CYP3A4 rs12721627, while none was homozygous for the variant T-allele. With respect to IHC results, only one tumor was identified as MSI-low (MSH6-negative, MSH2, MLH1, PMS2 positive), the rest being MSI-stable (all four proteins positive). CYP3A4 RQ values were always <31, which correspond to very low to undetectable transcript levels. Because of this finding, all tumors examined were considered as CYP3A4 non-expressors.

Out of the SNPs examined, none was associated with ABCB1 mRNA expression, while a T-insertion in intron 3 of the CYP2C8 gene in the neighborhood of rs11572080 (CYP2C8 c.7225G/A) was associated with increased expression of the corresponding mRNA (p = 0.007) (Table 6). CYP2C8 mRNA expression was higher in tumors with low Ki67 expression (p = 0.046). The presence of the ABCB1 1236T allele was preferentially found in ER and PgR positive tumors (p = 0.045 and p = 0.013, respectively), while the presence of the ABCB1 2677T allele was more frequent in PgR positive tumors (p = 0.036) (Table 7).

**Table 6:** Categorical (SNP and IHC) marker associations with mRNA RQ values (continuous variables).

		N	Median	Range	P-value
		**CYP2C8 RQ values**			
SNP, CYP2C8 intron 3	lns T	21	32.2	25.1–37.5	0.007
	No lns T	24	28.2	25.6–33.2	
IHC, Ki67 score	High (≥14%)	35	29	25.1–35.4	0.046
	Low (<14%)	11	30.5	27.0–37.5	
		**MAPT RQ values**			
IHC, Ki67 score	High (≥14%)	35	39.9	32.7–42.3	0.007
	Low (<14%)	11	41.1	39.9–42.4	
IHC, PgR	Negative	12	39.6	33.1–42.0	0.019
	Positive	34	40.5	32.7–42.4	
IHC, Tau protein	Negative	24	39.2	32.7–42.3	<0.001
	Positive	21	40.8	39.8–42.4	
IHC, TopoIIa	Negative	35	40.6	32.7–42.4	0.001
	Positive	11	38.4	33.1–40.2	
		**TUBB3 RQ values**			
SNP, ABCB1_2677G/A/T (rs2032582)	G or G/A	14	39.4	37.2–43.3	0.028
	T or T/G	30	38.3	29.0–41.2	
IHC, PgR	Negative	13	40.2	35.9–43.3	0.036
	Positive	31	38.1	29.0–41.4	
IHC, βIII-tubulin	Negative	30	37.6	29.0–41.1	0.002
	Positive	13	40.2	38.1–43.3	

**Table 7:** Associations between SNP and IHC markers.

		ER (IHC)			PgR (IHC)		
		Negative	Positive	P-value	Negative	Positive	P-value
ABCB1_1236C/T (rs1128503)	C	5 (62.5)	13 (25.0)	0.15	10 (55.6)	8 (20.0)	0.033
	T	1 (12.5)	11 (21.2)		2 (11.1)	10 (25.0)	
	T/C	2 (25.0)	28 (53.8)		6 (33.3)	22 (55.0)	
ABCB1_1236C/T (rs1128503)	C	5 (62.5)	13 (25.0)	0.045	10 (55.6)	8 (20.0)	0.013
	T or T/C	3 (37.5)	39 (75.0)		8 (44.4)	32 (80.0)	
ABCB1_3435C/T (rs1045642)	C or T/C	7 (87.5)	34 (66.7)	0.414	16 (88.9)	25 (62.5)	0.061
	T	1 (12.5)	17 (33.3)		2 (11.1)	15 (37.5)	
ABCB1_2677G/A/T (rs2032582)	G or G/A	5 (62.5)	15 (28.8)	0.103	10 (55.6)	10 (25.0)	0.036
	T or T/G	3 (37.5)	37 (71.2)		8 (44.4)	30 (75.0)	

Relatively high MAPT mRNA expression was observed in tumors with a low Ki67 score (p = 0.007), PgR-positive (p = 0.019) and TopoIIa-negative (p = 0.001), while it was strongly concordant with IHC positivity for the corresponding protein (p<0.001). TUBB3 transcript levels also strongly correlated with IHC positivity for βIII-tubulin (p = 0.002), while they were higher in PgR-negative tumors (p = 0.036) (Table 6).

### Biomarker Effects on Patient Outcome

None of the SNP, mRNA and IHC markers examined was associated with patient response to ixabepilone (both locally and centrally assessed response) or with drug-related neutropenia and sensory neuropathy. The statistical significance observed for the association of fewer responders (centrally assessed) among patients with tumors expressing high as compared to those expressing low CYP3A4 mRNA (median cut-off for high vs. low, p = 0.041) was biologically irrelevant because, as described above, CYP3A4 mRNA expression was practically absent in the tumors examined.

Median survival had not been reached in all cases. With respect to survival, among the SNP markers examined, germline ABCB1 1236T, 2677T and 3435T alleles, either homozygous or heterozygous, were associated with a favorable outcome in comparison to non-T alleles (log-rank, p = 0.056, p = 0.013 and p = 0.002, respectively) (Table 8). A favorable outcome was also observed for the intronic CYP2C8 2077G allele (G/G or G/A versions in intron 3); however, this effect was compared with only two patients who carried the corresponding A/A allele and had a very aggressive disease course. Hence, this result could not be further considered in the context of prognostic impact of this SNP. Longer survival was noticed for tumors expressing high compared to low MAPT transcript levels at the median RQ value cut-off (log-rank, p = 0.035). By contrast, high TUBB3 transcript levels (cut-off at 75% of RQ values) were expressed in tumors from patients with unfavorable outcome, although these results did not reach statistical significance (p = 0.072). Among protein markers, only tumor PgR positivity correlated significantly with longer survival (p = 0.022) (Table 8).

**Table 8:** Biomarker associations with patient survival (log-rank test).

						95% CI		
Marker	Category	N	Failed	% Alive at 12 months	Median (mo)	LL	UL	P-value
**ABCB1 1236C/T (rs1128503)**	C	19	10	68.4%	23.6	8.2	.	0.056
	T or T/C	43	13	81.2%	.	21.9	.	
**ABCB1 2677G/A/T (rs2032582)**	G or G/A	21	12	71.4%	17.2	11.2	25.5	0.013
	T or T/G	41	11	80.3%	.	27.3	.	
**ABCB1 3435C/T (rs1045642)**	C	14	10	71.4%	14.5	4.4	25.5	0.002
	T or T/C	47	13	78.5%	.	27.3	.	
**MAPT RQ values, 50% cut-off**	High	24	6	83.1%	.	23.6	.	0.035
	Low	24	14	62.5%	15.9	9.9	.	
**TUBB3 RQ values, 75% cut-off**	High	11	7	54.5%	12.4	4.4	.	0.072
	Low	35	12	79.7%	.	17.2	.	
**IHC, PgR**	Negative	19	12	57.9%	14.5	8.5	27.3	0.012
	Positive	41	13	80.2%	.	21.9	.	

CI, confidence interval; LL, lower limit; N, number; UL, upper limit; “.”, Not reached yet for the median; “.”, Cannot be calculated yet for the UL.

With respect to PFS, only IHC markers showed significant associations yet independently of treatment arm. Patients with tumors positive for ER and tau protein had longer PFS in comparison to patients with negative such tumors (log-rank, p = 0.015 and p = 0.003, respectively), while TopoIIa protein positivity was associated with unfavorable PFS (p = 0.003) (Table 9).

**Table 9:** Biomarker associations with patient progression-free survival (log-rank test).

						95% CI		
Marker	Category	N	Failed	% event free at 12 months	Median (mo)	LL	UL	P-value
**IHC, ER**	Negative	9	9	11.1%	5.5	0.8	10.2	0.015
	Positive	53	39	47.2%	9.8	6.5	14.5	
**IHC, Tau protein**	Negative	35	31	28.6%	6.5	5.5	9.8	0.003
	Positive	25	15	60.0%	16.4	9.2	.	
**IHC, TopoIIa**	Negative	47	34	48.9%	10.2	8.8	17.2	0.003
	Positive	15	14	20.0%	5.7	1.7	9.3	

CI, confidence interval; LL, lower limit; N, number; UL, upper limit; “.”, Cannot be calculated yet.

**Figure 4:**
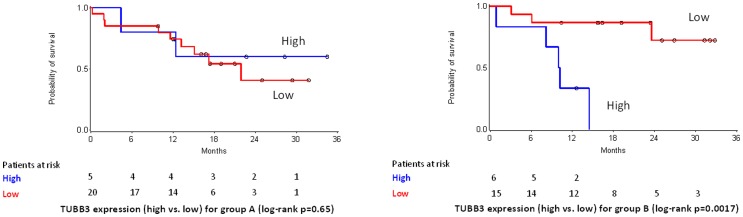
Predictive specificity of TUBB3 mRNA expression for survival in treatment Groups A (40 mg/m^2^, 3-weekly) and B (20 mg/m^2^, weekly). TUBB3 RQ value cut-off was set at 75%. Among patients in Group B, those with tumors expressing high TUBB3 transcript levels had significantly shorter survival.

Treatment arm-specific interactions between the examined SNP and protein markers were not observed (univariate Cox analysis). Among mRNA targets a significant interaction was observed for TUBB3 (p = 0.018). In particular, survival of Group B patients with tumors expressing high TUBB3 transcript levels (75% cut-off for RQ values) was significantly worse in comparison to patients in the same group with low TUBB3 mRNA expressing tumors (HR: 9.747; 95% CI: 2.162–43.933; Wald’s p = 0.003). The same marker was not predictive for survival in Group A patients (Figure 4).

All results from the three-step multivariate analysis are presented as supporting information (Table S7 for PFS and Table S8 for survival). Significant results were considered from the third step only, which included the largest number of patients. Thus, IHC ER positivity was a favorable (Wald’s p = 0.009) and IHC TopoIIa positivity an unfavorable (p = 0.002) prognostic factor for PFS. In addition, IHC PgR positivity was found to be favorable (p = 0.028) and a high number of metastatic sites marginally unfavorable (p = 0.056) for survival.

## Discussion

A number of phase II (reviewed in ref. [26]) and one randomized study [27] showed that ixabepilone has demonstrable activity in patients with MBC. Even though the approved by the FDA dose of 40 mg/m^2^ every 3 weeks is accompanied by a manageable toxicity profile, other schedules of administration have been tested in an attempt to improve the risk/benefit ratio. In the present study we report the final results on the activity and safety of two schedules, the approved one and the weekly schedule at a dose of 20 mg/m^2^. Unfortunately, the study was closed prematurely due to a low rate of accrual. This was probably due to the reluctance of physicians and patients to further participate in the study following the decision of Bristol-Myers Squibb, in March 2009, to withdraw the application to the European Medicines Agency for a centralized marketing authorization for ixabepilone and arrest its development in Europe.

The ORR of the 3-weekly and weekly schedules was 47% and 50%, respectively, which is in the range of that reported in some of the phase II studies [28], [29], albeit higher than that achieved in others [14], [30]. It is worthy of note, that in a randomized phase II study (reported only in abstract form) comparing the approved dose with that of 16 mg/m^2^ on days 1, 8 and 15 every 28 days, the ORR was 14% for the 3-weekly and 8% for the weekly schedule [31]. In the registration phase III trial [27], the combination of ixabepilone and capecitabine demonstrated significantly improved ORR compared to capecitabine monotherapy (35% vs. 14%, p<0.0001) with longer PFS (median 5.8 vs. 4.2 months, HR 0.75, p = 0.0003). Importantly, in a recent phase III trial (CALGB 40502), weekly ixabepilone (16 mg/m^2^) was found to have inferior PFS and greater toxicity compared to weekly paclitaxel (90 mg/m^2^), both given on a 3-weeks on, 1-week off schedule to chemotherapy naïve MBC patients [32]. Nevertheless, it has to be kept in mind that cross-study comparisons of response rates (or survival) is frequently misleading, since differences in important patient or tumor characteristics, study sample size, ethnicity and previous treatments in combination with other confounding factors may influence the results.

In general, ixabepilone, given as first line chemotherapy, has a manageable safety profile with neutropenia, sensory neuropathy, arthralgias/myalgias and fatigue being the most prominent adverse events (reviewed in ref. [15], [26]. Even though in almost all studies ixabepilone induced neutropenia in the vast majority of patients [28]–[30], [33], the incidence of febrile neutropenia appears to be low [15]. Of note, there were five cases of febrile neutropenia among the 64 patients treated with ixabepilone, one of them fatal in the 3-weekly schedule. In our study grade 3/4 neutropenia was recorded in 28% of the patients (32% in the 3-weekly vs. 23% in the weekly schedule). Even though the 23% incidence of grade 3/4 neutropenia, observed in the weekly schedule of 20 mg/m^2^ of our study, is lower compared to the standard 3-weekly schedule, it is nevertheless higher than that reported in a number of studies using a lower ixabepilone dose (16 mg/m^2^) in a weekly schedule, such as by Rugo et al: 11% [34], Smith et al: 7% [31] and Rugo et al, all grade 3+ hematologic toxicities: 20% [32]. The numerically higher incidence of grade 3/4 neutropenia observed in our study with the use of an ixabepilone dose of 20 mg/m^2^ in a weekly schedule, brings into question the appropriateness of this dose, as opposed to the 16 mg/m^2^ weekly dose used in most of the recent studies.

Sensory neuropathy induced by ixabepilone may be schedule-dependent and is usually reversible. In a recently published analysis on the incidence of peripheral neuropathy induced by ixabepilone in reported phase II/III trials, the rate of grade III–IV toxicity according to CRC-NCI criteria ranged from 1% in patients with early untreated breast cancer to 24% in heavily pretreated MBC [35]. Furthermore, in an interim safety analysis of the previously mentioned randomized phase II study [31], it was shown that the weekly administration was associated with higher (but non-significant) incidence of severe neuropathy compared to the 3-weekly schedule (20% vs. 11%). Likewise, in the present study, grade III sensory neuropathy occurred in 4/34 (11.8%) of the patients treated with the 3-weekly and in 8/30 (26.7%) with the weekly schedule. However, in all patients, except one, sensory neuropathy showed complete resolution to baseline or grade I after the completion of ixabepilone treatment.

The main route of metabolism of ixabepilone is via CYP3A4, with substances that inhibit CYP3A4 activity decreasing its metabolism [26]. The tumors examined here, however, expressed very low to undetectable CYP3A4 mRNA, while all patients carried the ancestral C-allele for rs12721627 in the same gene. It seems therefore unlikely that the CYP3A4 gene or its derivatives may have influenced the effect of ixabepilone in the present patient series. CYP2C8 is another cytochrome P450 gene that has been implicated in taxane metabolism [36] but has not as yet been explored in epothilones. In comparison to CYP3A4, CYP2C8 transcripts were detected at variable levels in our breast carcinoma samples and were preferentially high in tumors with low proliferation rates, as indicated by low Ki67 scores. A new variation was identified, concerning a T-insertion in the 3^rd^ intron of CYP2C8, which was strongly associated with increased expression of this gene. These observations may be useful when assessing the role of CYP2C8 in breast cancer, especially during treatment with taxanes or epothilones.

Other than the above cytochrome P450 components, all ABCB1 polymorphisms tested were associated with patient survival. Of note, in all cases, the presence of the variant T-allele seemed to confer longer survival. The presence of this variant T-allele has been associated with taxane toxicity in one study [37], however it was not related to paclitaxel toxicity in another [38], as was also the case with ixabepilone toxicity in the present study. ABCB1 has recently been described as a substrate for ixabepilone [39]. ABCB1 was overall expressed at low levels in the tumors examined, while the presence of the T-allele was not significantly associated with ABCB1 mRNA expression, except in the homozygotes. The association of the T variant with tumor PgR-positivity, which was per se a favorable prognostic factor for survival in this study, may have accounted for the observed better patient outcome. Indeed, none of the ABCB1 T variants remained prognostic for survival upon multivariate adjustment, while PgR-positivity, assessed by IHC, did. In any case, the association of the evolutionarily more recent ABCB1 T-alleles in the SNPs examined with PgR- and ER-positivity in breast carcinomas is a novel finding meriting further investigation.

In vitro studies suggest that the tau protein and paclitaxel both bind to the same pocket on the inner surface of the microtubules. Conceivably, low expression of the tau protein renders microtubules more sensitive to paclitaxel [40]–[42]. These data indicate that tau protein is a potential predictor of sensitivity to all microtubule-stabilizing agents, including ixabepilone. Indeed, low ER and MAPT mRNA expression were strongly predictive of sensitivity to ixabepilone in the neo-adjuvant setting [43], while ixabepilone seems to benefit patients with triple-negative breast cancer [44]. However, MAPT mRNA did not predict benefit from the addition of paclitaxel to epirubicin/CMF dose-dense adjuvant chemotherapy in a different study [45]. On the other hand, tau protein expression assessed by IHC was not related to ixabepilone response in a small phase II study in metastatic breast cancer [28]. In the present series MAPT mRNA and tau protein expression were strongly associated with each other, while high expression of either was associated with favorable outcome. This is in line with ER and PgR being favorable predictors in the present study, since MAPT expression is influenced by ER [46]. It is also in line with the inverse correlation of high MAPT with high Ki67 and TopoIIa-positivity, which are generally associated with poor prognosis. Evidently, all these pro-ER and pro-MAPT related findings appear to be discordant with the reports cited above. Sample size and consistency with respect to breast cancer molecular subtypes, as well as treatment setting may have accounted for this discrepancy. In support to the present findings, however, ER positive breast cancer cell lines were ixabepilone sensitive, while MAPT mRNA expression was not included in gene expression sets predictive of ixabepilone response in a very recent study [47]. Evidently, the issue of MAPT expression levels with respect to ixabepilone efficiency needs further clarification. From a different perspective, this study confirms the well-established good prognosis of ER/PgR-positive tumors, the association of MAPT expression with these favorable prognostic factors and the adverse prognostic effect of TopoIIa-positivity [48].

As previously stated, resistance to taxanes may develop via different mechanisms, such as MDR [49], β-tubulin mutations [50] or overexpression of the βIII-tubulin isoform [51], [52] or microtubule-associated proteins [53]. βΙΙΙ is one of the eight different isoforms of β-tubulin that have been identified so far and has been linked to paclitaxel resistance in vitro [54], [55]. In contrast to paclitaxel and to different epothilones, ixabepilone binds to βIII-tubulin containing microtubules and stabilizes them [11], [56] but its efficacy does not seem to be affected by βIII-tubulin in vitro [57]. At present, information about a potential link between βIII-tubulin expression and clinical activity of ixabepilone is limited. Herein we showed that TUBB3 mRNA and βIII-tubulin-positivity were strongly associated, but only transcript levels tended to correlate with prognosis (survival). Further, we noticed that high TUBB3 transcript levels were predictive of poor survival in patients treated with a cumulatively higher dose of ixabepilone (Group B, weekly schedule). However, since this finding is based on a small sample size it should be interpreted with caution. Whether ixabepilone efficacy is indeed dependent on TUBB3 transcript levels is an interesting question that needs to be answered and validated in larger multi-arm studies.

In this study we also investigated MMR status in primary tumors from patients treated with ixabepilone in the metastatic setting, based on previous reports suggesting that taxanes may benefit patients with MMR deficient tumors [58]. Other than reported elsewhere [59], [60], we observed loss of only one MMR protein, MSH6, and this in only one case. This finding is in line with a previous study in tumors from Greek patients that demonstrated no loss for the MMR proteins tested [61].

In conclusion, this study shows that ixabepilone had a manageable safety profile with neutropenia, sensory neuropathy, arthralgias/myalgias and fatigue being the most prominent adverse events. Out of the 5 genetic, 5 mRNA and 12 protein markers examined in this study, none was associated with patient ORR and drug toxicity. However, despite the descriptive use of p-values, which poses a limitation to the study conclusions, some interesting marker correlations and prognostic effects were observed that merit further investigation. These include: (a) the association of three ABCB1 variant T-alleles with PgR and ER-positivity, as well as with a better outcome for the corresponding patients; (b) the positive association of MAPT mRNA expression and tau protein expression with the ER-positive phenotype, and their corresponding favorable prognostic impact; (c) the inverse correlation of MAPT mRNA expression with TopoIIa protein expression, with TopoIIa positivity being an unfavorable prognostic factor in our study; (d) a positive interaction of TUBB3 mRNA expression with cumulatively high dose ixabepilone for patient survival. These markers were of potential merit in the prognostic setting in this small cohort of patients. However, they need to be further evaluated for their prognostic and/or predictive value and validated in larger multi-arm studies.

## Supporting Information

Figure S1 **REMARK diagram for biomarker studies in ixabepilone treated patients.**
(TIF)

Table S1 **Number of patients with adverse events (any grade).**
(DOC)

Table S2 **Kaplan-Meier estimates of PFS, TTP, TTF and survival.**
(DOC)

Table S3 **SNP frequencies (where applicable, SNPs located next to the main variants corresponding to the rs-ID indicated are presented).**
(DOC)

Table S4 **IHC results for all markers.**
(DOC)

Table S5 **FFPE tissue mRNA expression as continuous RQ values.**
(DOC)

Table S6 **mRNA expression represented by binary RQ values according to the cut-offs indicated (25%: lower quartile; 50%: median; 75%: upper quartile). RQ values were calculated as [40-dCT].**
(DOC)

Table S7 **Steps of the multivariate selection process for progression-free survival (PFS).**
(DOC)

Table S8 **Steps of the multivariate selection process for survival.**
(DOC)

Protocol S1 **Approved protocol for the trial.**
(PDF)

Checklist S1 **CONSORT checklist.**
(DOC)

Supporting Information S1 **Detailed eligibility criteria and treatment dose modifications.**
(DOC)

Supporting Information S2 **Detailed methodology for the assessment of translational biomarkers.**
(DOC)
