# Supplementary figures and images for: Ixabepilone Administered Weekly or Every Three Weeks in HER2-Negative Metastatic Breast Cancer Patients; A Randomized Non-Comparative Phase II Trial
Source: PLoS One. 2013 Jul 23;8(7):e69256. doi: 10.1371/journal.pone.0069256 (PMC3720651; doi:10.1371/journal.pone.0069256)

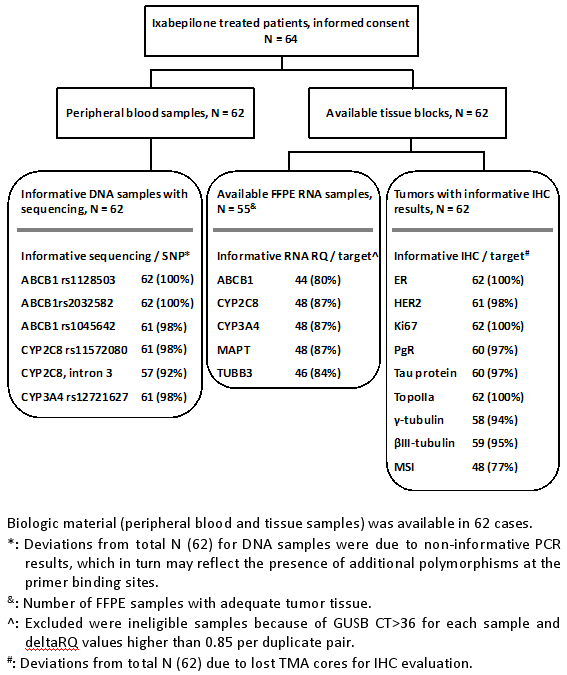

Supplement: Figure S1 — REMARK diagram for biomarker studies in ixabepilone treated patients. (TIF) [file pone.0069256.s001.tif]
