# Supplementary material for: Ixabepilone Administered Weekly or Every Three Weeks in HER2-Negative Metastatic Breast Cancer Patients; A Randomized Non-Comparative Phase II Trial
Source: PLoS One. 2013 Jul 23;8(7):e69256. doi: 10.1371/journal.pone.0069256 (PMC3720651; doi:10.1371/journal.pone.0069256)
Supplement: Table S1 — Number of patients with adverse events (any grade). (DOC) [file pone.0069256.s002.doc]

|  | **Safety Group** | | | | | | | | | |
| --- | --- | --- | --- | --- | --- | --- | --- | --- | --- | --- |
|  | **A: 40 mg/m^2^ (3-weekly)** | | | | | **B: 20 mg/m^2^ (weekly)** | | | | |
|  | **Grade** | | | | | **Grade** | | | | |
|  | **1** | **2** | **3** | **4** | **5** | **1** | **2** | **3** | **4** | **5** |
| ALT | 7 | 2 | . | . | . | 9 | 2 | 1 | . | . |
| AST | 8 | 2 | . | . | . | 8 | 2 | . | . | . |
| Acne | 1 | 2 | . | . | . | . | . | . | . | . |
| Alkaline phosphatase | 6 | 1 | . | . | . | 10 | 2 | . | . | . |
| Allergic Reaction | . | . | 2 | . | . | 1 | . | 1 | . | . |
| Alopecia | 4 | 12 | . | . | . | 3 | 5 | . | . | . |
| Anorexia | 1 | 3 | . | . | . | 3 | 3 | . | . | . |
| Bilirubin | 1 | . | 1 | . | . | 2 | 1 | 1 | . | . |
| Blurred vision | 1 | . | . | . | . | . | . | . | . | . |
| CPK | . | . | 1 | . | . | . | . | . | . | . |
| Cardiac ischemia/infarction | . | . | 1 | . | 1 | . | . | . | . | . |
| Cholesterol | 4 | . | . | . | . | 5 | . | . | . | . |
| Cognitive disturbance | 1 | . | . | . | . | . | . | . | . | . |
| Constipation | 4 | 1 | 1 | . | . | 9 | . | . | . | . |
| Cough | 5 | 1 | 1 | . | . | 6 | 1 | . | . | . |
| Creatinine | 2 | . | . | . | . | 2 | 1 | . | . | . |
| Dermatology-Other ( | 2 | . | . | . | . | 2 | . | . | . | . |
| Diarrhea | 3 | 3 | 1 | . | . | 4 | 4 | 1 | . | . |
| Dizziness | 3 | . | . | . | . | 1 | 1 | . | . | . |
| Dyspnea | 1 | 1 | 1 | 1 | . | 3 | . | . | . | . |
| Edema: limb | 1 | . | . | . | . | 1 | . | . | . | . |
| Fatigue | 9 | 2 | 3 | . | . | 5 | 6 | 5 | . | . |
| Febrile neutropenia | . | . | 1 | . | 1 | . | . | 3 | . | . |
| Fever | 2 | 2 | . | . | . | 2 | 5 | . | . | . |
| GGT | 5 | 3 | . | . | . | 6 | 6 | 1 | . | . |
| GI-Other | 1 | . | . | . | . | . | . | . | . | . |
| Anemia | 13 | 6 | . | 1 | . | 8 | 9 | 1 | . | . |
| Hemorrhage – Other | 1 | . | . | . | . | 1 | . | . | . | . |
| Hot flashes | 1 | . | . | . | . | . | . | . | . | . |
| Hypercalcemia | 2 | . | . | . | . | 1 | . | . | . | . |
| Hyperglycemia | 7 | . | . | . | . | 9 | 5 | . | . | . |
| Hyperkalemia | 1 | 1 | 2 | . | . | 4 | 1 | . | . | . |
| Hypernatremia | 3 | . | . | . | . | 1 | . | . | . | . |
| Hypertriglyceridemia | 7 | . | . | . | . | 3 | . | . | . | . |
| Hyperuricemia | 2 | . | . | . | . | 3 | . | . | . | . |
| Hypoalbuminemia | 1 | 1 | . | . | . | 5 | 1 | . | . | . |
| Hypocalcemia | 5 | . | . | . | . | . | 1 | . | . | . |
| Hypokalemia | . | . | 1 | 1 | . | 2 | . | 1 | . | . |
| Hyponatremia | 5 | . | 1 | . | . | 7 | . | 2 | . | . |
| Hypophosphatemia | 1 | 1 | . | . | . | . | . | 1 | . | . |
| Hypotension | 1 | . | . | 1 | . | 2 | . | . | . | . |
| Infection (documented clinically) | 1 | . | . | . | . | . | . | 3 | . | . |
| Infection with normal ANC | . | 3 | . | . | . | . | 2 | 2 | . | . |
| Infection-Other | 4 | 2 | . | . | 1 | 4 | 1 | 1 | . | . |
| Insomnia | . | . | 1 | . | . | . | . | . | . | . |
| Leukopenia | 6 | 8 | 5 | 1 | . | 4 | 7 | 7 | 1 | . |
| Lymphatics-Other | . | 1 | . | . | . | 1 | . | . | . | . |
| Lymphopenia | 2 | . | . | . | . | 1 | 1 | . | . | . |
| Metabolic/Lab – Other | 4 | 3 | 3 | . | . | 9 | 2 | . | . | . |
| Mucositis (clinical exam) | 3 | 1 | 1 | . | . | 2 | 1 | . | . | . |
| Musculoskeletal-Other | . | 1 | . | . | . | . | . | 1 | . | . |
| Nail changes | . | 1 | 1 | . | . | 4 | 2 | 1 | . | . |
| Nausea | 8 | 4 | . | . | . | 9 | 3 | . | . | . |
| Neuropathy-motor | . | . | 2 | . | . | 1 | 1 | 1 | . | . |
| Neuropathy-sensory | 7 | 13 | 4 | . | . | 5 | 11 | 8 | . | . |
| Neuropathy: cranial | 1 | . | . | . | . | . | . | . | . | . |
| Neutropenia | 1 | 7 | 10 | 1 | . | 3 | 8 | 4 | 3 | . |
| Ocular surface disease | 1 | . | . | . | . | 1 | . | . | . | . |
| PTT | 1 | . | . | . | . | . | . | 1 | . | . |
| Pain | 4 | 6 | 3 | . | . | 6 | 8 | . | . | . |
| Pain-Other | 2 | 4 | . | . | . | 6 | . | 1 | . | . |
| Pericarditis | . | 1 | . | . | . | . | . | . | . | . |
| Thrombocytopenia | 1 | 1 | . | . | . | 3 | . | . | . | . |
| Rash | . | 2 | . | . | . | 1 | 1 | . | . | . |
| Renal failure | . | . | 1 | . | . | . | . | . | . | . |
| Somnolence | . | 3 | . | . | . | . | . | . | . | . |
| Supraventricular arrhythmia | 1 | 2 | . | . | . | . | 1 | . | . | . |
| Taste alteration | . | 3 | . | . | . | 1 | 2 | . | . | . |
| Voice changes | 1 | 1 | . | . | . | . | . | . | . | . |
| Vomiting | 2 | 1 | 2 | . | . | 2 | . | . | . | . |
| Weight gain | 1 | 1 | . | . | . | 1 | . | 1 | . | . |
| Weight loss | . | 2 | . | . | . | 3 | . | . | . | . |
| Blood-Other | . | . | . | . | . | 1 | . | . | . | . |
| Cardiac Arrhythmia-Other | . | . | . | . | . | 1 | . | . | . | . |
| Cardiac General-Other | . | . | . | . | . | 2 | . | . | . | . |
| Edema: head and neck | . | . | . | . | . | 1 | . | . | . | . |
| Flatulence | . | . | . | . | . | 2 | . | . | . | . |
| Flu-like syndrome | . | . | . | . | . | 1 | 1 | . | . | . |
| Fracture | . | . | . | . | . | . | 1 | . | . | . |
| Hemorrhage pulmonary | . | . | . | . | . | 1 | . | . | . | . |
| Hemorrhoids | . | . | . | . | . | 1 | . | . | . | . |
| Hypermagnesemia | . | . | . | . | . | 1 | . | 1 | . | . |
| Hypoglycemia | . | . | . | . | . | 1 | . | . | . | . |
| Hypomagnesemia | . | . | . | . | . | 1 | . | . | . | . |
| Hypothyroidism | . | . | . | . | . | . | 1 | . | . | . |
| Incontinence, anal | . | . | . | . | . | 1 | . | . | . | . |
| Injection site reaction | . | . | . | . | . | 1 | . | . | . | . |
| Left ventricular systolic dysfunction | . | . | . | . | . | 1 | . | . | . | . |
| Memory impairment | . | . | . | . | . | 1 | . | . | . | . |
| Muscle weakness | . | . | . | . | . | 1 | . | . | . | . |
| Neurology-Other | . | . | . | . | . | 1 | . | . | . | . |
| Ocular-Other | . | . | . | . | . | . | 1 | . | . | . |
| Opportunistic infection | . | . | . | . | . | . | . | 1 | . | . |
| Palpitations | . | . | . | . | . | 2 | . | . | . | . |
| Pulmonary-Other | . | . | . | . | . | . | . | . | 1 | . |
| Rhinitis | . | . | . | . | . | 1 | . | . | . | . |
| Rigors/chills | . | . | . | . | . | 3 | . | . | . | . |
| Tinnitus | . | . | . | . | . | 1 | . | . | . | . |
| Tremor | . | . | . | . | . | 1 | . | . | . | . |
| Vaginitis | . | . | . | . | . | 1 | . | . | . | . |
| Watery eyes | . | . | . | . | . | . | 1 | . | . | . |
