# Supplementary material for: Ixabepilone Administered Weekly or Every Three Weeks in HER2-Negative Metastatic Breast Cancer Patients; A Randomized Non-Comparative Phase II Trial
Source: PLoS One. 2013 Jul 23;8(7):e69256. doi: 10.1371/journal.pone.0069256 (PMC3720651; doi:10.1371/journal.pone.0069256)
Supplement: Table S2 — Kaplan-Meier estimates of PFS, TTP, TTF and survival. (DOC) [file pone.0069256.s003.doc]

|  | Group A  (3-weekly) | Group B  (weekly) |
| --- | --- | --- |
| Progression-free survival (PFS) | 9 months  (95% CI 4-14; range 0.2-31+) | 12 months  (95% CI 6-28; range 1-33+) |
| Time to progression (TTP) | 10 months  (95% CI 6-14; range 0.2-31+) | 14 months  (95% CI 6-not reached; range 1-33+) |
| Time to treatment failure (TTF) | 5 months  (95% CI 4-7; range 1-11) | 4 months  (95% CI 3-6; range 0-9) |
| Survival | 26 months  (95% CI 13-not reached; range 0.2-35+) | Not reached  (95% 24-not reached; range 1-33+) |
