# Supplementary material for: Ixabepilone Administered Weekly or Every Three Weeks in HER2-Negative Metastatic Breast Cancer Patients; A Randomized Non-Comparative Phase II Trial
Source: PLoS One. 2013 Jul 23;8(7):e69256. doi: 10.1371/journal.pone.0069256 (PMC3720651; doi:10.1371/journal.pone.0069256)
Supplement: Table S3 — SNP frequencies (where applicable, SNPs located next to the main variants corresponding to the rs-ID indicated are presented). (DOC) [file pone.0069256.s004.doc]

| **Available peripheral blood DNA samples. n=62** | | **N** | **%** |
| --- | --- | --- | --- |
| **ABCB1 rs1128503, 1236C/T, C-allele** | **C or T/C** | 49 | 79,0 |
|  | **T** | 13 | 21,0 |
|  | **Total** | 62 | 100,0 |
| **ABCB1 rs1128503, 1236C/T, 3-scale** | **C** | 19 | 30,6 |
|  | **T** | 13 | 21,0 |
|  | **T/C** | 30 | 48,4 |
|  | **Total** | 62 | 100,0 |
| **ABCB1 rs1128503, 1236C/T, T-allele** | **C** | 19 | 30,6 |
|  | **T or T/C** | 43 | 69,4 |
|  | **Total** | 62 | 100,0 |
| **ABCB1 rs2032582, 2677G/A/T, G/T vs. GA** | **G or T or T/G** | 59 | 95,2 |
|  | **G/A** | 3 | 4,8 |
|  | **Total** | 62 | 100,0 |
| **ABCB1 rs2032582, 2677G/A/T, T-homozygotes** | **G or G/A or T/G** | 48 | 77,4 |
|  | **T** | 14 | 22,6 |
|  | **Total** | 62 | 100,0 |
| **ABCB1 rs2032582, 2677G/A/T, 4-scale** | **G** | 18 | 29,0 |
|  | **G/A** | 3 | 4,8 |
|  | **T** | 14 | 22,6 |
|  | **T/G** | 27 | 43,5 |
|  | **Total** | 62 | 100,0 |
| **ABCB1 rs2032582, 2677G/A/T, T-allele** | **G or G/A** | 21 | 33,9 |
|  | **T or T/G** | 41 | 66,1 |
|  | **Total** | 62 | 100,0 |
| **ABCB1 rs1045642, 3435C/T, C-allele** | **C or T/C** | 42 | 68,9 |
|  | **T** | 19 | 31,1 |
|  | **Total** | 61 | 100,0 |
| **ABCB1 rs1045642, 3435C/T, 3-scale** | **C** | 14 | 23,0 |
|  | **T** | 19 | 31,1 |
|  | **T/C** | 28 | 45,9 |
|  | **Total** | 61 | 100,0 |
| **ABCB1 rs1045642, 3435C/T, T-allele** | **C** | 14 | 23,0 |
|  | **T or T/C** | 47 | 77,0 |
|  | **Total** | 61 | 100,0 |
| **CYP2C8 rs11572080, 7225G/A, 3-scale** | **A** | 3 | 4,9 |
|  | **G** | 41 | 67,2 |
|  | **G/A** | 17 | 27,9 |
|  | **Total** | 61 | 100,0 |
| **CYP2C8 rs11572080, 7225G/A, A-allele** | **G** | 41 | 67,2 |
|  | **G/A or A** | 20 | 32,8 |
|  | **Total** | 61 | 100,0 |
| **CYP2C8 rs11572080, 7225G/A, G-allele** | **A** | 3 | 4,9 |
|  | **G/A of G** | 58 | 95,1 |
|  | **Total** | 61 | 100,0 |
| **CYP2C8, intron 3, T-insertion (next to rs11572080)** | **lns T** | 27 | 44,3 |
|  | **no lns T** | 34 | 55,7 |
|  | **Total** | 61 | 100,0 |
| **CYP3A4 rs12721627, 20716C/G** | **C** | 61 | 100,0 |
|  | **G** | 0 | 0 |
|  | **Total** | 61 | 100,0 |
| **CYP2C8, intron 3, 2077A/G, 3-scale (next to rs11572080)** | **A** | 2 | 3,5 |
|  | **A/G** | 28 | 49,1 |
|  | **G** | 27 | 47,4 |
|  | **Total** | 57 | 100,0 |
| **CYP2C8, intron 3, 2077A/G, A-allele (next to rs11572080)** | **A/G or A** | 30 | 52,6 |
|  | **G** | 27 | 47,4 |
|  | **Total** | 57 | 100,0 |
| **CYP2C8, intron 3, 2077A/G, G-allele (next to rs11572080)** | **A** | 2 | 3,5 |
|  | **A/G of G** | 55 | 96,5 |
|  | **Total** | 57 | 100,0 |
