# Supplementary material for: Ixabepilone Administered Weekly or Every Three Weeks in HER2-Negative Metastatic Breast Cancer Patients; A Randomized Non-Comparative Phase II Trial
Source: PLoS One. 2013 Jul 23;8(7):e69256. doi: 10.1371/journal.pone.0069256 (PMC3720651; doi:10.1371/journal.pone.0069256)
Supplement: Table S4 — IHC results for all markers. (DOC) [file pone.0069256.s005.doc]

| **Available tissue blocks. n=62** | | **N** | **%** |
| --- | --- | --- | --- |
| **ER** | **Negative** | 9 | 14,5 |
|  | **Positive** | 53 | 85,5 |
|  | **Total** | 62 | 100,0 |
| **HER2** | **Negative** | 54 | 88,5 |
|  | **Positive** | 7 | 11,5 |
|  | **Total** | 61 | 100,0 |
| **Ki67** | **High (≥14%)** | 46 | 74,2 |
|  | **Low (<14%)** | 16 | 25,8 |
|  | **Total** | 62 | 100,0 |
| **PgR** | **Negative** | 19 | 31,7 |
|  | **Positive** | 41 | 68,3 |
|  | **Total** | 60 | 100,0 |
| **Tau protein** | **Negative** | 35 | 58,3 |
|  | **Positive** | 25 | 41,7 |
|  | **Total** | 60 | 100,0 |
| **TopoIIa** | **Negative** | 47 | 75,8 |
|  | **Positive** | 15 | 24,2 |
|  | **Total** | 62 | 100,0 |
| **γ-tubulin** | **Negative** | 29 | 50,0 |
|  | **Positive** | 29 | 50,0 |
|  | **Total** | 58 | 100,0 |
| β**III-tubulin** | **Negative** | 41 | 69,5 |
|  | **Positive** | 18 | 30,5 |
|  | **Total** | 59 | 100,0 |
| **MSI** | **Low (MSH6-negative)** | 1 | 2,0 |
|  | **Stable** | 47 | 98,0 |
|  | **Total** | 48 | 100,0 |
