# Supplementary material for: Ixabepilone Administered Weekly or Every Three Weeks in HER2-Negative Metastatic Breast Cancer Patients; A Randomized Non-Comparative Phase II Trial
Source: PLoS One. 2013 Jul 23;8(7):e69256. doi: 10.1371/journal.pone.0069256 (PMC3720651; doi:10.1371/journal.pone.0069256)
Supplement: Table S5 — FFPE tissue mRNA expression as continuous RQ values. (DOC) [file pone.0069256.s006.doc]

| **Available mRNA samples N=55** | **Descriptives** | | | | | | | |
| --- | --- | --- | --- | --- | --- | --- | --- | --- |
|  | **N (%)** | **Mean** | **Standard Deviation** | **Min** | **Lower quartile** | **Median** | **Upper quartile** | **Max** |
| **ABCB1 RQ (40-dCTavg)** | 44 (80) | 31.1 | 2.8 | 26.2 | 29.0 | 31.0 | 33.7 | 36.0 |
| **CYP2C8 RQ (40-dCTavg)** | 48 (87.3) | 30.3 | 3.4 | 25.1 | 27.4 | 29.4 | 33.3 | 37.5 |
| **CYP3A4 RQ (40-dCTavg)** | 48 (87.3) | 27.8 | 1.6 | 24.7 | 26.5 | 27.7 | 29.1 | 30.8 |
| **MAPT RQ (40-dCTavg)** | 48 (87.3) | 39.6 | 2.2 | 32.7 | 38.1 | 40.2 | 41.0 | 42.4 |
| **TUBB3 RQ (40-dCTavg)** | 46 (83.6) | 38.5 | 2.3 | 29.0 | 37.2 | 38.6 | 40.1 | 43.3 |

**Note:** Numbers in parenthesis in column N represent percentage of informative measurements for each mRNA target.
