# Supplementary material for: Ixabepilone Administered Weekly or Every Three Weeks in HER2-Negative Metastatic Breast Cancer Patients; A Randomized Non-Comparative Phase II Trial
Source: PLoS One. 2013 Jul 23;8(7):e69256. doi: 10.1371/journal.pone.0069256 (PMC3720651; doi:10.1371/journal.pone.0069256)
Supplement: Table S6 — mRNA expression represented by binary RQ values according to the cut-offs indicated (25%: lower quartile; 50%: median; 75%: upper quartile). RQ values were calculated as [40-dCT]. (DOC) [file pone.0069256.s007.doc]

|  | | **N** | **%** |
| --- | --- | --- | --- |
| **ABCB1 25%** | **High** | 33 | 75,0 |
|  | **Low** | 11 | 25,0 |
|  | **Total** | 44 | 100,0 |
| **ABCB1 50%** | **High** | 22 | 50,0 |
|  | **Low** | 22 | 50,0 |
|  | **Total** | 44 | 100,0 |
| **ABCB1 75%** | **High** | 11 | 25,0 |
|  | **Low** | 33 | 75,0 |
|  | **Total** | 44 | 100,0 |
| **CYP2C8 25%** | **High** | 36 | 75,0 |
|  | **Low** | 12 | 25,0 |
|  | **Total** | 48 | 100,0 |
| **CYP2C8 50%** | **High** | 24 | 50,0 |
|  | **Low** | 24 | 50,0 |
|  | **Total** | 48 | 100,0 |
| **CYP2C8 75%** | **High** | 12 | 25,0 |
|  | **Low** | 36 | 75,0 |
|  | **Total** | 48 | 100,0 |
| **CYP3A4 25%** | **High** | 36 | 75,0 |
|  | **Low** | 12 | 25,0 |
|  | **Total** | 48 | 100,0 |
| **CYP3A4 50%** | **High** | 24 | 50,0 |
|  | **Low** | 24 | 50,0 |
|  | **Total** | 48 | 100,0 |
| **CYP3A4 75%** | **High** | 12 | 25,0 |
|  | **Low** | 36 | 75,0 |
|  | **Total** | 48 | 100,0 |
| **MAPT 25%** | **High** | 36 | 75,0 |
|  | **Low** | 12 | 25,0 |
|  | **Total** | 48 | 100,0 |
| **MAPT 50%** | **High** | 24 | 50,0 |
|  | **Low** | 24 | 50,0 |
|  | **Total** | 48 | 100,0 |
| **MAPT 75%** | **High** | 12 | 25,0 |
|  | **Low** | 36 | 75,0 |
|  | **Total** | 48 | 100,0 |
| **TUBB3 25%** | **High** | 34 | 73,9 |
|  | **Low** | 12 | 26,1 |
|  | **Total** | 46 | 100,0 |
| **TUBB3 50%** | **High** | 23 | 50,0 |
|  | **Low** | 23 | 50,0 |
|  | **Total** | 46 | 100,0 |
| **TUBB3 75%** | **High** | 11 | 23,9 |
|  | **Low** | 35 | 76,1 |
|  | **Total** | 46 | 100,0 |
