# Supplementary material for: Ixabepilone Administered Weekly or Every Three Weeks in HER2-Negative Metastatic Breast Cancer Patients; A Randomized Non-Comparative Phase II Trial
Source: PLoS One. 2013 Jul 23;8(7):e69256. doi: 10.1371/journal.pone.0069256 (PMC3720651; doi:10.1371/journal.pone.0069256)
Supplement: Table S7 — Steps of the multivariate selection process for progression-free survival (PFS). (DOC) [file pone.0069256.s008.doc]

| **Parameter** | | **PFS models** | | | | | |
| --- | --- | --- | --- | --- | --- | --- | --- |
|  |  | **Step 1** | | **Step 2** | | **Step 3** | |
|  |  | **Model with backward selection (n=42)** | | **Exclude MAPT RQ values (n=56)** | | **Exclude MAPT RQ values, ABCB1 1236C/T (rs1128503) and Tau protein (n=58)** | |
|  |  | **HR** | **P-value** | **HR** | **P-value** | **HR** | **P-value** |
| **ABCB1 1236C/T (rs1128503)** | **C vs. T or T/C** | 3.7 | 0.006 | - | - | - | - |
| **IHC, ER** | **Positive vs. negative** | 0.3 | 0.056 | 0.3 | 0.007 | 0.3 | 0.009 |
| **Group** | **B: 20 mg/m^2^ (weekly) vs. A: 40 mg/m^2^ (3-weekly)** | 0.3 | 0.003 | - | - | - | - |
| **MAPT RQ values (50% cut-off)** | **High vs. Low** | 2.2 | 0.087 | - | - | - | - |
| **IHC, Tau protein** | **Positive vs. Negative** | 0.2 | 0.003 | - | - | - | - |
| **IHC, TopoIIa** | **Positive vs. Negative** | - | - | 3.3 | 0.002 | 3.3 | 0.002 |

HR, hazard ratio
