# Supplementary material for: Ixabepilone Administered Weekly or Every Three Weeks in HER2-Negative Metastatic Breast Cancer Patients; A Randomized Non-Comparative Phase II Trial
Source: PLoS One. 2013 Jul 23;8(7):e69256. doi: 10.1371/journal.pone.0069256 (PMC3720651; doi:10.1371/journal.pone.0069256)
Supplement: Table S8 — Steps of the multivariate selection process for survival. (DOC) [file pone.0069256.s009.doc]

| **Parameter** | | **Survival models** | | | | | |
| --- | --- | --- | --- | --- | --- | --- | --- |
|  |  | **Step 1** | | **Step 2** | | **Step 3** | |
|  |  | **Model with backward selection**  **(n=42)** | | **Exclude TUBB3 RQ values**  **and MAPT RQ values**  **(n=56)** | | **Exclude TUBB3 RQ values, MAPT RQ values, ABCB1 3435C/T (rs1045642) and Tau protein (n=58)** | |
|  |  | **HR** | **P-value** | **HR** | **P-value** | **HR** | **P-value** |
| **ABCB1 3435C/T (rs1045642)** | **T or T/C vs. C** | 0.01 | 0.0001 | 0.25 | 0.002 | - | - |
| **Group*TUBB3 RQ values (75% cut-off)** | **Interaction term** | 121.51 | 0.003 | - | - | - | - |
| **Group** | **B (weekly) vs. A (3-weekly) for TUBB3 low** | 0.22 | 0.071 | - | - | - | - |
| **TUBB3 RQ values (75% cut-off)** | **High vs. Low for group A (3-weekly)** | 0.11 | 0.039 | - | - | - | - |
| **IHC, PgR** | **Positive vs. Negative** |  |  |  |  | 0.41 | 0.028 |
| **IHC, Tau protein** | **Positive vs. Negative** | 0.03 | 0.001 | 0.41 | 0.068 | - | - |
| **IHC, TopoIIa** | **Positive vs. Negative** | 0.18 | 0.093 | - | - | - | - |
| **Visceral metastasis** | **Yes vs. No** | 0.12 | 0.019 | - | - | - | - |
| **Multiple metastases** | **>3 vs. 1-3** | 0.15 | 0.050 | - | - | 2.23 | 0.056 |

HR, hazard ratio.
