# Supplementary material for: Ixabepilone Administered Weekly or Every Three Weeks in HER2-Negative Metastatic Breast Cancer Patients; A Randomized Non-Comparative Phase II Trial
Source: PLoS One. 2013 Jul 23;8(7):e69256. doi: 10.1371/journal.pone.0069256 (PMC3720651; doi:10.1371/journal.pone.0069256)
Supplement: Protocol S1 — Approved protocol for the trial. (PDF) [file pone.0069256.s010.pdf]

Phase II randomized trial of ixabepilone administered weekly or every three weeks in patients with HER-2 negative metastatic breast cancer previously treated with chemotherapy in the neo-adjuvant or adjuvant setting
HE 11/A08

Trial Conducting Collaborative Group
Hellenic Cooperative Oncology Group [HeCOG] (leading group)
18 Hatzikonstanti Street, 115 24 Athens, Greece
Tel +30 210 6912 520
Fax +30 210 6912 713

Study Head and Principal Investigator
George Fountzilas, M.D.
Professor of Medical Oncology
University of Thessaloniki
Greece

Co-Investigator
Evangelos Briasoulis, M.D.
Assistant Professor of Oncology
University of Ioannina
Greece

CONTENTS

I. INTRODUCTION....4

A. IXABEPILONE....4
1. IXABEPILONE PHASE II CLINICAL DATA IN BREAST CANCER....4
2. IXABEPILONE PHASE III CLINICAL DATA IN BREAST CANCER....8
3. PRELIMINARY RESULTS OF PHASE II TRIALS WITH WEEKLY IXABEPILONE ADMINISTRATION....9

II. STUDY RATIONALE....11

III. TRANSLATIONAL RESEARCH STUDIES RATIONALE....11

IV. TRIAL DESIGN....12

A. REGULATORY ISSUES....12
B. PURPOSE AND ELIGIBILITY....12
1. OBJECTIVES....12
2. ENDPOINTS....13
3. ELIGIBILITY CRITERIA....13
C. TREATMENT PLAN....15
1. RANDOMIZATION....15
2. TREATMENT PROTOCOL....15
3. TREATMENT DURATION....16
4. RE-TREATMENT CRITERIA AND DOSE MODIFICATIONS FOR THE ONCE EVERY 3 WEEKS DOSING ARM (ARM A)....16
5. RE-TREATMENT CRITERIA AND DOSE MODIFICATIONS FOR THE WEEKLY DOSING ARM (ARM B)....17
6. DOSING DELAYS....17
7. TREATMENT DISCONTINUATION....17
8. CONCOMITANT TREATMENTS....18
9. PATIENT FOLLOW-UP....18
10. DATA MANAGEMENT AND MONITORING OF STUDY....18
11. PLANNED TIMETABLE....18
12. TARGET PATIENT POPULATION....19
13. NUMBER OF PATIENTS....19
14. INVESTIGATIONAL TREATMENT....19

V. TRANSLATIONAL RESEARCH STUDIES....22

A. PHARMACOGENOMICS....22
B. PREDICTIVE BIOMARKERS FOR RESPONSE....22
C. STUDY OF ANTI-ANGIOGENIC EFFECTS....22

VI. CLINICAL ASSESSMENTS....23

A. EFFICACY....23

B. TOXICITY 23
1. SERIOUS ADVERSE EVENTS 23
2. TOXIC DEATHS 25
3. EVALUATION OF TOXICITY 25

VII. EVALUATION AND VISIT SCHEDULE 26

VIII. STATISTICAL DESIGN AND METHODS 27

A. SAMPLE SIZE CALCULATION .... ERROR! BOOKMARK NOT DEFINED.
B. DEFINITIONS 27
C. ANALYSES 28

IX. REFERENCES 29

X. ETHICS AND GENERAL TRIAL CONDUCTION ASPECTS 32

A. ETHICAL ASPECTS 32
1. GENERAL ISSUES 32
2. INFORMED CONSENT 32
3. ETHICS COMMITTEES 32
4. CONDITIONS FOR MODIFYING THE PROTOCOL 32
5. CONDITIONS FOR TERMINATING THE STUDY 33
B. STUDY DOCUMENTATION AND RECORD KEEPING 33
1. INVESTIGATOR'S FILES / RETENTION OF DOCUMENTS 33
2. SOURCE DOCUMENTS AND BACKGROUND DATA 33
3. AUDITS AND INSPECTIONS 33
4. CASE REPORT FORMS – DATA FLOW 34
5. CONFIDENTIALITY OF TRIAL DOCUMENTS AND PATIENT RECORDS 35
6. PUBLICATION OF DATA AND PROTECTION OF TRADE SECRETS 35
C. ADMINISTRATIVE RESPONSIBILITIES 35

XI. APPENDICES 36

A. RESPONSE EVALUATION CRITERIA IN SOLID TUMORS 36
B. WORLD MEDICAL ASSOCIATION DECLARATION OF HELSINKI 40
C. PATIENT INFORMATION SHEET 43
D. NCI COMMON TOXICITY CRITERIA VERSION 3 47

I. INTRODUCTION

Breast cancer is a leading cause of cancer-related mortality in women[1]. The death toll of breast cancer can be ameliorated to some extend by administering appropriate adjuvant systemic therapy in patients who had an early-stage breast cancer resected[2, 3]. Firstly CMF and later anthracycline-based regimens have been proven to decrease the risk of relapse and cancer-related mortality in women with early-stage breast cancer[4]. Lately post surgical adjuvant chemotherapy has been managed to enhance its life-saving effects with the incorporation of taxanes into adjuvant regimens[5-9]. Today, as data get mature over time, the taxanes are found to add benefit in both disease-free and overall survival over standard chemotherapy, which takes them steadily to the standard clinical practice. It is now foreseeable that in the near future the majority of patients with early-stage breast cancer will be treated with a taxane in the adjuvant setting[10-12].
However, the successful incorporation of the taxanes into adjuvant chemotherapy has deprived patients who relapse from a class of highly active drugs and has left treating oncologists short of therapeutic options. This fact brings a new scene in the therapy of metastatic breast cancer, with limited therapeutic options practically left, particularly for patients with steroid receptor-negative and triple-negative tumors. Clinical research is now up to face this challenge with the development of new drugs. Among others, epothilones are rigorously tested as potential replacements of taxanes in patients with metastatic breast cancer previously treated with a taxane in the adjuvant setting.

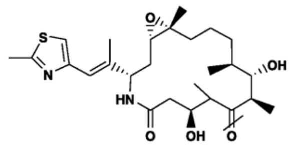

Ixabepilone (BMS-247550-01)

Epothilones are microtubule-stabilizing drugs that come with credentials to fill the emerging therapeutic gap. The epothilones are macrolide fermentation products of the myxobacterium sorangium cellulosum, which compete paclitaxel in stabilizing microtubules and cause cell cycle arrest and cytotoxicity[13]. Ixabepilone (BMS-247550, picture) is a semisynthetic analogue of epothilone B, in which the lactone oxygen of epothilone B is replaced by nitrogen to increase drug stability[14]. Ixabepilone has been found to be active in paclitaxel-resistant breast cancer in preclinical models[15-17]. In humans ixabepilone has demonstrated promising antitumor activity in metastatic breast cancer and an acceptable safety profile in previously untreated patients and also in patients treated with and resistant to taxanes[18-20].

A. IXABEPILONE

1. Ixabepilone Phase II Clinical Data in Breast Cancer

Four phase II clinical studies (CA163009, CA163010, CA163031 and CA163081) have evaluated ixabepilone 40 mg/m^2 every 3 weeks in subjects with metastatic breast cancer. In these studies ixabepilone demonstrated promising activity either as a single agent (CA163009, CA163010, and CA163081) or in combination with capecitabine (CA163031) (see also table 2).
The monotherapy studies are presented in greater detail in this section. Further details of these studies and the other BMS sponsored studies are provided in the Investigator Brochure.

The monotherapy study CA163010 was conducted in subjects with MBC who were anthracycline pre-treated (in adjuvant and neo-adjuvant setting). Subjects were dosed with ixabepilone at 40 mg/m^2 as a 3-hour infusion every 3 weeks. The primary endpoint was response rate. Tumor evaluation was performed every 2 cycles. Dosing continued every 3 weeks as long as subjects tolerated the treatment and were without evidence of progressive disease up to a maximum of 18 cycles. Sixty-five subjects were response-evaluable, 27 achieved a PR to ixabepilone therapy for a RR of 41.5% (Confidence Interval (CI) =29.4-54.4%) and 24 (35%) subjects achieved disease stabilization. The majority of responses were achieved within 12 weeks of initiating therapy. Of the 27 responders, 20 first met criteria for response within 6 weeks of first ixabepilone dose, 5 subjects first met criteria between 10 and 12 weeks of initiating therapy and the last 2 subjects first met response criteria between 14 and 16 weeks after the initiation of ixabepilone. The median duration of response was 8.2 months (95% CI=5.7-10.2 months). The median time to progression for all treated subjects was 4.8 months and the median overall survival was 22.0 months (95% CI=15.6-27.0 months). Treatment related adverse events were manageable and mostly Grade ½. The most common adverse event excluding alopecia was neuropathy. Treatment-related neuropathy was primarily sensory, cumulative and reversible and most often mild to moderate in severity. Sensory neuropathy was Grade ½ in 33 (51%) subjects and Grade 3 in 13 (20%) subjects. No subjects had Grade 4 sensory neuropathy. Motor neuropathy was uncommon: Grade 2 in 1 (2%) subject and Grade 3 in 3 (5%) subjects. No subjects had Grade 4 motor neuropathy. Eighteen (28%) subjects discontinued because of sensory neuropathy, however this occurred after a median of 6 cycles (range 1-10). Other commonly reported Grade ¾ treatment related adverse events include myalgia (8%), vomiting (6%), infection with Grade ¾ neutropenia (6%), fatigue (6%), arthralgia (5%), neuropathic pain (5%) stomatitis/pharyngitis (5%) and febrile neutropenia (5%). Among the responders, 8 discontinued therapy due to disease progression, 4 were withdrawn by investigators and 15 discontinued due to ixabepilone related toxicity (11 of them due to neurotoxicity).

The monotherapy study CA163009 was conducted in subjects with MBC who had received an anthracycline and were resistant to paclitaxel and/or docetaxel. All subjects had taxane-based chemotherapy as their most recent treatment and had progressed within 4 months of their last dose of the taxane-containing regimen (within 6 months if this regimen was administered in either the adjuvant or neo-adjuvant setting). At the beginning of the study, ixabepilone was administered as a 50 mg/m^2 infusion over 1 hour every 3 weeks. Due to observations from other studies, the schedule was subsequently changed to 40 mg/m^2 over 3 hours every three weeks. Only data from the subject cohort treated after this modification is reported here. The primary objective was to assess the clinical activity of ixabepilone, as measured by the tumor response rate. Dosing continued every 3 weeks as long as subjects tolerated the treatment, unless there was evidence of progressive disease (PD) or the subject met discontinuation criteria. Subjects with stable disease (SD) or partial response (PR) were treated until either disease progression or up to a maximum of 18 cycles of treatment. Subjects who achieved a complete response (CR) were treated for up to a maximum of 4 cycles post CR. Among the 49 treated and response-evaluable subjects on this regimen, 6 achieved a PR to ixabepilone therapy (RR=12.2%; 95% CI=4.7-26.5%). The 6 responders received a median of 10.5 cycles (range 5.0-15.0 cycles) of ixabepilone therapy and had a median duration of response of 10.4 months (95% CI=6.3-22.0 months). Stable

disease was reported as the best overall response for 20 (41%) subjects treated on this regimen. The median time to progression for all subjects treated on the 40 mg/m^2 over 3 hours regimen was 2.2 months (95% CI=1.4-3.2 months) and the median survival was 7.9 months (95% CI=6.1-14.5 months). Treatment-related adverse events were manageable and mostly mild to moderate in severity (Grade ½). Treatment-related neuropathy was mostly sensory, and generally mild to moderate, cumulative, and reversible. Sensory neuropathy was Grade 3 in 6 (12%) subjects. No subjects had Grade 4 sensory neuropathy. 5 (10%) subjects discontinued ixabepilone therapy because of neuropathy after a median of 6 cycles (range 3-8). Although follow-up was limited in some subjects, treatment-limiting or severe neuropathy generally resolved or lessened in intensity within 1-2 months after discontinuing ixabepilone therapy. Grade ¾ treatment-related adverse events (other than neuropathy) that occurred in ≥ 5% of subjects included fatigue (27%), myalgia (10%), nausea (6%) and vomiting (6%), none of which were treatment-limiting. Hematologic abnormalities were manageable: The most common Grade ¾ abnormality was neutropenia, which was Grade 3 in 33% and Grade 4 in 20% of subjects. Treatment-related febrile neutropenia was reported in 2 (4%) subjects and led to discontinuation of ixabepilone in 1 (2%) subject. Grade 4 anemia (4%) and thrombocytopenia (2%) were uncommon. Eight (16%) subjects were discontinued because of treatment-related adverse events. Five (10%) subjects died on-study or within 30 days of last dose; none of the deaths were judged related to ixabepilone.

The monotherapy study CA163081 was conducted in subjects with MBC who were resistant to an anthracycline (or were not candidates for further treatment with an anthracycline) and who were resistant to taxanes and capecitabine. Ixabepilone was administered as monotherapy at 40 mg/m^2 every 21 days. Objective antitumor activity was confirmed by an IRRC. IRRC confirmed ORR was 11.5% in response-evaluable subjects. The ORR as assessed by the investigator was 18.3% for all treated subjects. Tumor responses were durable with a median duration of response of 5.3 months. Responders had extensive baseline disease and disease resistant to an anthracycline, a taxane, and capecitabine as well as other agents commonly used to treat metastatic breast cancer (eg, vinorelbine, gemcitabine). Adverse events were manageable and primarily mild to moderate (Grade ½). The most common adverse event was peripheral neuropathy. Consistent with other studies, peripheral neuropathy was primarily sensory, mild to moderate (Grade ½) in severity, cumulative, and reversible. At baseline, peripheral neuropathy was common in this heavily-pretreated population (25% Grade 1, 2% Grade 2). Peripheral neuropathy was Grade ½ in 49%, Grade 3 in 13%, and Grade 4 in only 1 (1%) subject and led to discontinuation in 7 (6%) subjects. Peripheral neuropathy (Grade 3 or higher) was reversible with a median time to improvement (by 1 grade) of 4.6 weeks and a median time to resolution (to Grade 1 or baseline) of 5.4 weeks. Hematologic toxicity, consisting mostly of neutropenia and leukopenia, was manageable and did not contribute notably to dose reductions or discontinuations. Neutropenia was common (31% Grade 3; 23% Grade 4). Febrile neutropenia (4 subjects, 3%) and infection with Grade ¾ neutropenia or leukopenia (3 subjects, 2%) were uncommon. One subject died from infection with Grade 4 neutropenia in Cycle 1. Anemia was primarily mild to moderate in severity and was often present at baseline (30% Grade 1, 3% Grade 2). Severe thrombocytopenia was uncommon (7%).

The combination study CA163031 was conducted in subjects with MBC previously

treated with a taxane and an anthracycline. The aim was to determine the recommended Phase II (and III) dose and schedule of ixabepilone and capecitabine. The data reported in Tables 1 and 2 refer to the cohort treated with the dose and schedule which was finally recommended for subsequent studies, 40 mg/m^2 every 3 weeks of ixabepilone, and 2000 mg/m^2 of capecitabine given orally on Days 1–14 of a 21-day schedule.

A summary of adverse events and response data in metastatic breast cancer is presented in Tables 1 and 2, respectively.

Table 1: Treatment Related Grade ¾ Adverse Events in MBC Studies

Adverse Event	CA163010 (n = 65)	CA163009 (n = 49)	CA163081 (n = 126)	CA163031 (n = 62)
	Grade ¾ (%)	Grade ¾ (%)	Grade ¾ (%)	Grade ¾ (%)
Neutropenia	58	53	54	57
Febrile neutropenia	5	6	2	4
Sensory neuropathy	20	12	14	18
Diarrhea	3	8	1	8
Arthralgia	5	2	2	2
Myalgia	8	10	7	24
Fatigue	6	27	10	14
Stomatitis	5	4	6	5

Table 2: Summary of Tumor Responses in Phase II Therapy for MBC

			Number (%) of Subjects		
**Study (population)**	**Dose/schedule**	**N**	**Complete Response**	**Partial Response**	**Stable Disease**
CA163009 - (MBC) Taxane-resistant	40 mg/m^2/3h q3w	49	0	6 (12%)	20 (41%)
CA163010 - (MBC) Prior anthracycline in adjuvant, taxane-sensitive	40 mg/m^2/3h q3w	65	0	27 (42%)	23 (35%)

Table 2: Summary of Tumor Responses in Phase II Therapy for MBC

Study (population)	Dose/schedule	N	Number (%) of Subjects Complete Response	Partial Response	Stable Disease
CA163081 (MBC) Resistant to anthracyclines, taxanes and capecitabine	40 mg/m^2/3h q3w	113	0	23 (18%)[a]	55 (44%)
CA163031 (Phase I/II) - (MBC) Anthracycline/taxane-pretreated	Ixabepilone: 40 mg/m^2/3h q3w and Capecitabine: 2000 mg/m^2 daily for 14 day q3w	50[b]	1 (2%)	15 (30%)	16 (32%)

[a] Assessed by investigator, all treated subjects

[b] 62 pts treated include 12 subjects with non-measurable disease

In addition to the described phase II studies in MBC, one study was conducted in the neo-adjuvant setting.

The neo-adjuvant monotherapy study CA163080 was conducted in subjects with invasive breast cancer whose tumors were not amenable to breast conservation surgery. The primary objective of the study was to analyze the pre-treatment expression of mRNA from tumor samples collected from subjects and identify potential predictors of response to ixabepilone administered in the neo-adjuvant setting. Eligible subjects received neo-adjuvant therapy with ixabepilone at 40 mg/m^2 administered as a 3-hour intravenous (IV) infusion on Day 1 of each 21-day cycle for up to 4 continuous cycles. Clinical tumor response in the primary site and in the axilla was assessed after each cycle prior to the next treatment. A total of 161 subjects received at least 1 dose of ixabepilone. Among all treated subjects, 61% achieved a best overall response of CR or PR and 35% were non-responders (i.e. SD or PD), 18% achieved pathological complete response in the breast and 11% achieved pathological complete response in both breast and lymph nodes. In this subject population, treatment with 40 mg/m^2 of ixabepilone resulted in an acceptable safety profile when administered as neo-adjuvant therapy for a total of 4 cycles. Most AEs were Grade 1 or Grade 2. Grade ¾ AEs were reported in 25% of subjects overall. Grade ¾ neutropenia were reported in 21 (13%) subjects, Grade ¾ leukopenia in 13 (8%) subjects and Grade 3 peripheral neuropathy in 5 (3%) subjects. In the subjects with Grade 3 peripheral neuropathy, the toxicity grade improved after dosing was stopped, and with the exception of one subject, the neuropathy completely resolved in 2 to 9 months. In one subject, peripheral neuropathy was unresolved at the last contact 3 months after the last ixabepilone dose.

2. Ixabepilone Phase III Clinical Data in Breast Cancer

Ixabepilone has been tested in a large phase III clinical trial in anthracycline and taxane

resistant breast cancer (Vahdat et al. Journal of Clinical Oncology, 2007 ASCO Annual Meeting Proceedings Part I. Vol 25, No. 18S (June 20 Supplement), 2007: 1006)
In this large multinational phase III trial, patients with MBC who were anthracycline pre-treated and met predefined resistance criteria to taxanes were randomized to ixabepilone (40mg/m^2 IV over 3h Q3w) + capecitabine (1,000mg/m^2 PO BID Q14d) or capecitabine (1,250mg/m^2 PO BID Q14d). The primary endpoint was progression-free survival (PFS); secondary endpoints included objective response rate (ORR), safety, and overall survival (available after 2007). Response and progression were assessed by an independent review committee (IRC) and the investigators (INV).
752 patients were randomized. Median age was 53; 84% had visceral disease, 48% and 43% had 1 and =2 prior metastatic regimens. Median of 5 and 4 cycles of ixabepilone + capecitabine and capecitabine were administered.
Ixabepilone + capecitabine were superior to capecitabine alone. Significant benefit was consistently maintained across predefined subgroups, including HER2-/ER-/PR- and HER2+. The primary analysis of PFS resulted in a hazard ratio= 0.75 in favour of the combination arm (5.8 months versus 4.2 months, p=0.0003). ORR was 35% in the combination arm and 14% in the capecitabine only arm (p<0.0001).
Grade (G) ¾ adverse events included neuropathy (ixabepilone + capecitabine 23% vs capecitabine 0%), hand-foot syndrome (18% vs 17%), and fatigue (9% vs 3%). Neuropathy was cumulative and reversible (median time to resolution of G3/4 to baseline/G1 was 6 weeks). G3 and 4 neutropenia were reported in 32% and 36% vs 9% and 2%, respectively; febrile neutropenia was 5% with ixabepilone + capecitabine. Toxic death rate was 3% vs 1%. Patients with liver dysfunction were at greater risk.

3. Preliminary results of phase II trials with weekly ixabepilone administration

Preliminary data are now available from 4 NCI/CTEP sponsored Phase II clinical studies of ixabepilone administered in a weekly schedule (NCI-5342, NCI-4470, NCI-5913, E3803 and E2301). The NCI-5342 clinical trial is a phase II study conducted in patients with indolent and mantle cell lymphoma that were refractory to standard therapy.
Treatment consisted of ixabepilone at 25 mg/m^2 over 1 hour on days 1, 8 and 15 every 28 days. Dose reductions to 20 and 17.5 mg/m^2 were allowed for patients with drug related grade ≥ 3 toxicity. Preliminary results were presented for 18 patients, 3 patients had follicular lymphoma, 3 had small lymphocytic lymphoma/chronic lymphocytic leukemia (SLL/ CLL) and 12 had mantle cell lymphoma. The overall response rate was 33% in this population. Patients came off study after a median of 2 cycles frequently as a result of toxicity. The following adverse events were reported in the 18 patients: thrombocytopenia grade 3/4 (5 patients); leukopenia grade 3/4 (10); neutropenia grade 3/4 (9); lymphopenia grade 3 (12); and 1 patient had grade 3 sensory neuropathy.
Grade 1/2 adverse events included diarrhea (6); constipation (4); fatigue (9); sensory neuropathy (4) and nausea (4).[21]
In the phase II, NCI-5913 study, ixabepilone is administered to patients with relapsed, aggressive non-Hodgkin's lymphomas (diffuse large B cell lymphoma, mantle cell lymphoma, and follicular grade 3 lymphoma). The treatment regimen consisted of ixabepilone 20 mg/m^2 given intravenously over 1 hour on Days 1, 8, and 15 of a 28 day cycle on an outpatient basis. To date, 27 patients have been enrolled, 10 patients have

died and 17 patients were alive at last assessment. One patient had rapidly progressive disease and died before completing cycle 1. All patients were heavily pretreated and at least 3 patients had failed a prior autologous stem cell transplant. All treated patients received a median of 2 cycles (range 1–5) of ixabepilone. Of 25 evaluable patients,1 patient achieved a CR, 7 a PR, 8 stable disease (SD) and 9 progressed. However, of the patients with radiologic SD, at least one patient had become PET negative and successfully underwent a matched sibling allogeneic transplant. The median time to progression and the progression-free survival was 108 days, and the overall survival was399 days. The main toxicities were hematologic (grade 3/4 toxicities were as follows: neutropenia (8), leukopenia (8) and lymphopenia (2). Nine patients had grade 3 peripheral neuropathy, 1 grade 3 neuralgia, and 1 grade 3 unilateral hearing loss. All toxicities, except the patient with a grade 3 unilateral hearing loss, resolved to baseline with cessation of drug. Other significant grade 3/4 AEs reported include fatigue (9 patients), anemia (5), infection (3), syncope (2), dermatitis exfoliative (2), and single reports of dehydration, dyspnea, diarrhea, vomiting, AST, ALT and non-cardiac and non-pleuritic chest pain (Smith S, personal communications).

The NCI-4470 study treated a total of 25 patients with advanced melanoma. Patients were stratified into previously untreated (n = 13) and prior therapy subgroups (n = 12). Patients in the pre-treated subgroup must have received DTIC or temazolomide and may have received a maximum of 2 prior chemotherapies. The protocol was written with the dose of ixabepilone set at 25 mg/m^2 on days 1, 8 and 15 every 28 days (28-day cycle). Dose reductions were allowed to 20 mg/m^2. The first patient enrolled, who had no prior therapy, was dosed at 25 mg/m^2. The patient developed septic shock and died prior to starting cycle 2. As a consequence of this toxicity, the protocol was revised and all subsequent patients received ixabepilone at 20 mg/m^2. At 20 mg/m^2 weekly for 3 weeks followed by 1 week rest the schedule was tolerated. Grade 3/4 toxicities across the 2 subgroups included sensory neuropathy (2 subjects, 8%), neutropenia (3, 12%), leukopenia (2, 12%), diarrhea (2, 12%), dyspnea (2, 12%) and other grade 3/4 toxicities reported in single patients included arrhythmias, fatigue, hypotension, thrombocytopenia and pneumonitis. No objective response was reported in this study. Eighteen patients progressed after 2 cycles, 5 patients received 3-6 cycles and had stable disease, but were removed due to toxicity (Median TTP = 8 weeks) and 2 patients were not evaluable for response assessment.[22]

E3803 is a trial conducted by ECOG in patients with refractory prostate cancer. Thirty-three chemo naive patients and 37 patients with prior taxane therapy were treated with ixabepilone at doses of 20 mg/m^2 intravenously over 1 hour weekly for 3 weeks followed by 1-week rest. Safety has been reported for the first 67 enrolled patients. Grade ¾ neutropenia was reported in 12 (17.7%); grade 3/4 neuropathy in 12 (17.7%); grade 3 fatigue in 14 (20.9%) grade 3 diarrhea in 6 (0.1%) and grade 3 nausea in 6 (0.1%). A PSA response of 40.6% in the no prior chemotherapy arm was observed. Other efficacy data for this study is currently not available.[23]

E2301 is a trial conducted in patients with metastatic or recurrent squamous cell cancer of the head and neck. This study assessed activity of ixabepilone in this patient population in 2 schedules: ixabepilone administered as 6 mg/m2 daily x 5 days every 21 days or at a dose of 20 mg/m^2 weekly for 3 consecutive weeks followed by 1 week rest. The schedule with 20 mg/m^2 weekly for 3 consecutive weeks followed by 1 week rest

demonstrated objective partial response and stable disease (PR = 5/52; SD = 17/52) and the daily x 5 q3 weeks schedule (PR = 1/32; SD = 12/32). Ixabepilone in both schedules was tolerated. In the daily x 5 schedule, grade 3/4 toxicities included neutropenia 1 (3%), anemia 3 (9.4%), fatigue 5 (15.6%), sensory neuropathy 1 (3%), and nausea 2 (6%) and in the weekly schedule grade 3/4 toxicities included neutropenia 8 (15.4%), anemia 3 (5.8%), fatigue 14 (26.9%), sensory neuropathy 4 (7.6%), motor neuropathy 12 (23%) nausea 7 (13.6%) and diarrhea 3 (6%).[24]

II. STUDY RATIONALE

The objectives of the present randomized phase II study are to evaluate the efficacy and toxicity of two administration schedules of ixabepilone given to HER-2 negative metastatic breast cancer (MBC) patients. that were previously treated with chemotherapy in the adjuvant or neo-adjuvant setting. Our previous experience with weekly paclitaxel over the last 10 years, that has later been shared by others, warrants investigation of a split dose “weekly” schedule against the standard three-weekly administration of ixabepilone, at an early stage of drug development, in a non-comparative trial[26-28]. Studies with weekly ixabepilone administration already in numerous indications also support the investigation in MBC. In addition to the clinical part of the trial, we will conduct translational research studies, in an effort to identify possible predictors of response and investigate kinetics of pro-angiogenic and anti-angiogenic proteins as surrogate biomarkers of angiogenesis in the two treatment arms[29-31].

III. TRANSLATIONAL RESEARCH STUDIES RATIONALE

Translational research studies will be a major part of this clinical trial. We plan to assess tumor samples for predictive biomarkers. Tubulin stabilization, acetylated alpha-tubulin, beta-3-microtubulin, Tau-1, p53, Bcl2, Bax, TACC3, and multidrug transporters will be assessed[18, 27]. Also, given the clinical significance of the anti-angiogenic effects of cytotoxic chemotherapy, we will investigate the effect of each dosing schema on circulating surrogate biomarkers of angiogenesis[33], such as VEGF, FGFb and TSP1.
Ixabepilone, as a microtubule stabilizer, is expected and already known to cause peripheral neuropathy, which is poorly characterized[34]. Moreover, a potential neurological toxicity is of major concern in patients who have previously been exposed to neurotoxic chemotherapy [taxanes]. Early studies suggest that ixabepilone related neuropathy, similarly to the taxanes, is schedule dependent[35]. Therefore, the incidence, characterization, severity, and reversibility of peripheral neuropathy will be prospectively assessed in this trial[36-38]. In addition, genetic polymorphisms in drug-metabolizing enzymes and drug transporters, such as the CYP3A and ABCB1 genes, will be explored in blood DNA and their association with the risk to develop treatment-related peripheral neuropathy and neutropenia will be assessed in each treatment arm.

IV. TRIAL DESIGN

This is a Phase II Randomized, Open Label, Non-comparative Trial. (Parallel Assignment and Efficacy Study).
Patients will be randomized to receive Ixabepilone either every three weeks, or weekly followed by one week off. Stratification factors include time to recurrence from adjuvant treatment and age at diagnosis.
150 patients will be enrolled in the study. Patients will be treated until consent withdrawal, intolerable toxicity or documented disease progression.

A. REGULATORY ISSUES

This trial has been designed and will be conducted according to the World Medical Association Declaration of Helsinki Good Clinical Practice. [*Appendix* B] The trial will be approved by the National Ethics Committee [NEC] and will be registered with *ClinicalTrials.Gov* [http://www.clinicaltrials.gov] and *EudraCT* [http://eudract.emea.eu.int/] prior to initiation.
Local institutional review boards (IRB) must approve the trial according to European Directives and National Law before it is allowed to start at each participating center. All patients will sign the NEC and IRB approved informed consent before registering. [*Appendix* C]

B. PURPOSE and ELIGIBILITY

The purpose of this clinical study is to evaluate the activity of ixabepilone when given at the recommended dose, on Day 1 of a 21-day cycle, or at half the dose on Days 1, 8 and 15 of a 28-day cycle, in patients with metastatic HER-2 negative breast cancer. who had been treated with adjuvant or neo-adjuvant chemotherapy.

1. Objectives

This is a randomized phase II clinical and translational research study.

a) Clinical Trial Primary objective

The primary objective is to assess the clinical activity of ixabepilone administered weekly or every 3 weeks in female patients with Metastatic Breast Cancer not previously treated in the metastatic setting.

b) Clinical Trial Secondary objective

- Assess the safety profile of both regimens concerning hematological and non-hematological toxicities

c) Translational Research objectives

- Explore the association of genetic polymorphisms in drug-metabolizing enzymes and drug transporters with the risk to develop treatment-related peripheral neuropathy and neutropenia
- Define molecular characteristics of responsiveness to treatment, by assessing selected biomarkers of potentially predictive value in tumor samples

- Investigate kinetics of pro-angiogenic and anti-angiogenic proteins in the two treatment arms
- Investigate for associations between molecular data and survival

2. Endpoints

a) Clinical Trial Endpoints

The primary endpoint will be:
Best Overall Response
Secondary endpoints will be:

- Efficacy endpoints
  - Time to Response
  - Progression-free survival (PFS)
  - Time to Treatment Failure (TTF)
  - Duration of response
  - Overall survival
- Toxicity endpoints
  - Non-hematological toxicities: estimation of incidence of neuropathy for both arms
  - Hematological toxicity: estimation of incidence of leukopenia, anemia, neutropenia and thrombocytopenia for both arms

b) Translational Research Endpoints

- Analysis of genetic polymorphisms in drug-metabolizing enzymes and drug transporters from blood DNA
- Assessment by immunohistochemistry of tubulin related proteins, oncogenic proteins and multidrug transporter proteins from tumor samples
- Assessment by quantitative real-time PCR of the alpha- and beta-tubulin genes from tumor samples
- ELISA measurements of circulating biomarkers of angiogenesis from blood samples

3. Eligibility criteria

a) Inclusion Criteria:

For inclusion into the study the following criteria must be met:

- Written informed consent
- Female patients aged 18 to 75 years inclusive
- Prior neo-adjuvant or adjuvant chemotherapy

- Diagnosis of HER-2 negative (HER-2 <2+ by immunohistochemistry and/or FISH negative) metastatic breast adenocarcinoma confirmed by the pathology department of the enrolling institution
- Eastern Cooperative Oncology Group performance status of 0 or 1
- Life expectancy of at least 12 weeks
- Measurable disease by the Response Criteria in Solid Tumors (RECIST) method

- Laboratory values within the specified ranges within 1 week of study enrolment:
    - Absolute neutrophil count of ≥ 1.5 x 10^9/L
- Thrombocyte count of ≥ 100 x 10^9/L
- Subjects must not have received cytotoxic chemotherapy for locally recurrent/metastatic disease
- Prior hormonal therapy for locally recurrent or metastatic disease allowed
- AST and ALT ≤ 2.5 x ULN
- Bilirubin ≤ 1.5 x ULN
- Recovery from prior palliative radiotherapy for bone metastases

b) Exclusion Criteria:

- Because of concerns that ixabepilone metabolism may be inhibited by potent cytochrome P450 3A4 inhibitors, patients must not receive the following medications, up to 72 hours prior to initiation of study therapy and until they come off treatment with ixabepilone: amprenavir, delavirdine, voriconazole, erythromycin, cyclosporine, troleandomycin, terfenadine, ketoconazole, nelfinavir, and ritonavir
- Patients with CTC grade 2 or greater neuropathy at baseline
- Patients with any history or evidence of brain an/or leptomenigneal metastasis
- Patients with clinically significant cardiac disease (e.g. unstable angina, congestive heart failure, myocardial infarction) within 6 months from study entry
- Psychiatric disorders or other conditions rendering the subject incapable of complying with the requirements of the protocol
- Any concurrent active malignancy other than non-melanoma skin cancer or in situ carcinoma of the cervix (subjects with a history of previous malignancies but without evidence of disease for 5 years will be allowed to enter the trial)
- Prior severe HSR to agents containing Cremophor EL
- Women of childbearing potential (WOCBP) who are unwilling or unable to use an adequate method of contraception to avoid pregnancy throughout the study and for up to 12 weeks from the last dose of ixabepilone, in such a manner that the risk of pregnancy is minimized

  WOCBP include: any female who has experienced menarche and who has not undergone successful surgical sterilization (hysterectomy, bilateral tubal ligation or bilateral oophorectomy) or is not postmenopausal (defined as amenorrhea ≥ 12 consecutive months; women on hormone replacement therapy with documented FSH level > 35mIU/mL. Even women who are practising abstinence or whom their partner is sterile (e.g. vasectomy) should be considered of childbearing potential.
- Women who are pregnant or breastfeeding
- Women with a positive pregnancy test on enrolment or prior to study therapy
- No other concomitant chemotherapy, endocrine therapy, immunotherapy, radiation therapy (except for palliative radiotherapy for bone metastases) or investigational treatments are allowed during subject’s participation in the study

C. TREATMENT PLAN

1. Randomization

Patients who fulfil the eligibility criteria, and have signed inform consent for the trial will be centrally randomized by electronic means to one of two ixabepilone treatment arms. Stratification factors will include: time to recurrence from adjuvant treatment, calculated from the date of the last dose of adjuvant treatment to the date of relapse (≤ 1 year vs. > 1 year); and previous chemotherapy with taxane regimen (yes vs. no). Randomization will be balanced by site.

2. Treatment Protocol

- **Arm A** [standard once every three weeks schedule]:

Ixabepilone [BMS-247550] will be administered on Day 1 (D1) every three weeks as a 3-hour infusion at a dose of 40 mg/m^2. [39-41]

- **Arm B** [weekly schedule]:

Ixabepilone [BMS-247550] will be administered weekly for three weeks as a 3-hour infusion at a dose of 20 mg/m^2, followed by one week-off. [42]

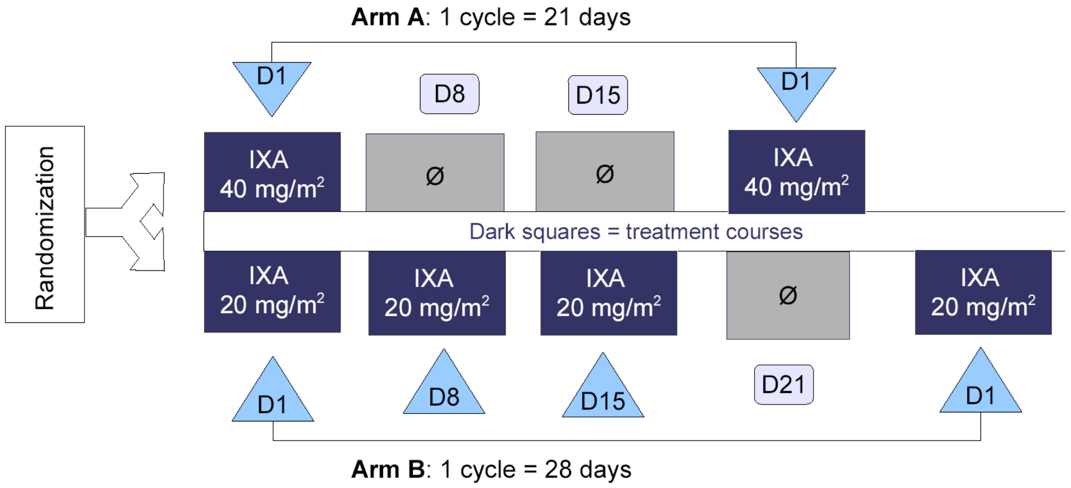

Body surface area (BSA) should be recalculated prior to each cycle of dosing. In calculating surface areas actual height and weight should be used; that is, there should be no adjustment to "ideal" weight. BSA will be capped at 2.2 m^2.

Supportive Treatment: Hypersensitivity prophylaxis will be given prior to the infusion of ixabepilone. All patients will receive the following premedication:

- an H1 antagonist (e.g., chlorpheniramine 10 mg, intravenously, or dexchlorpheniramine 2 mg orally, or equivalent) and
- an H2 antagonist (e.g., ranitidine 150-300 mg orally or 50 mg intravenously, or equivalent)

Additional premedication with corticosteroids (e.g. dexamethasone 20 mg intravenously, 30-60 minutes before infusion or orally, 6-12 hours before infusion) is required for patients who have experienced a hypersensitivity reaction in any previous cycle.
Prophylactic antiemetics will not be routinely administered, but can be added to the

regimen in patients who experienced toxicity. Granulocyte colony-stimulating factors (G-CSF) will not be given initially at cycle 1, but could be added to subsequent treatment cycles in patients with febrile neutropenia or delayed neutrophil recovery requiring a dose delay. Use of G-CSF should not replace the dose modification schema. Growth factor use must be consistent with product label.

3. Treatment Duration

Treatment can be continued until consent withdrawal by the patient, intolerable toxicity or documented disease progression.

4. Re-treatment criteria and dose modifications for the once every 3 weeks dosing arm (Arm A)

Re-treatment criteria

Patients should not begin a new cycle of treatment unless the **neutrophil** count is at least 1,500 cells/mm^3 and the **platelet** count is at least 100,000 cells/mm^3 (see section 4.3), and non-hematological toxicities have improved.

Dose modifications

Dose reductions will be implemented based on non-hematological toxicity or blood counts according to the following table. If toxicities recur after the initial dose reduction, an additional 20% dose reduction is recommended. If toxicities recur after the second dose reduction, ixabepilone must be discontinued.

Table 3: Dose Adjustments for Toxicities

	**Suggested Dose Modification**
**Non-hematological:**	
Grade 2 neuropathy (moderate) lasting ≥ 7 days	Decrease the dose by 20%
Grade 3 neuropathy (severe) lasting < 7 days	Decrease the dose by 20%
Grade 3 neuropathy (severe) lasting ≥ 7 days or disabling neuropathy	Discontinue treatment
Any Grade 3 toxicity (severe) other than neuropathy or transient Grade 3 arthralgia/myalgia and fatigue	Decrease the dose by 20%
Any Grade 4 toxicity (disabling)	Discontinue treatment
**Hematological:**	
Neutrophils < 500 cells/mm^3 for ≥ 7 days	Decrease the dose by 20%
Febrile neutropenia	Decrease the dose by 20%
Platelets < 25,000/mm^3 or platelets < 50,000/mm^3 with bleeding	Decrease the dose by 20%

5. Re-treatment criteria and dose modifications for the weekly dosing arm (Arm B)

In the arm with weekly dosing re-treatment **within a cycle** will be allowed **at a reduced dose,** if ANC is between 1000/mm^3 and 1499/mm^3 and/or platelets between 75,000/mm^3 and 99,999/mm^3 and treatment related non-hematological toxicities are ≤ grade 2 except for grade 2 neuropathy. Subjects with **ongoing ≥ grade 2 neuropathy** will not be retreated. Patients with ≤ Grade 2 neuropathy who previously experienced Grade 2 neuropathy lasting < 7 days will be re-treated at the same dose. Patients with prior grade 2 neuropathy lasting ≥ 7 days will require dose reduction. Missed doses within a cycle will not be made-up and should not influence the duration of the treatment cycle.

Table 4: Dose Modifications for Weekly Re-treatment (intra-cycle) in Ixabepilone Arm B (weekly treatment)

Hematological Toxicity					
**Neutrophils (mm^3)**		**Platelets (mm^3)**		**Neurotoxicity**	**Ixabepilone Weekly Dose (Arm)**
≥ 1500	**AND**	≥ 100,000	**AND**	<Gr 2 or Gr 2 < 7 days and resolved to < Gr 2	Maintain dose
1000 - 1499	**AND/OR**	75,000 - 99,999	**AND/OR**	Gr 2 ≥ 7 days and resolved to < Gr 2	Decrease 1 level
< 1000[a]	**OR**	< 75,000[a]	**OR**	Unresolved Gr 2	OMIT

[a] If treatment is held, repeat CBC until neutrophils ≥ 1500/mm^3 and platelets ≥ 100,000/mm^3 prior to re-treatment.

6. Dosing delays

Toxicities (including neurotoxicity) require resolution to grade 1 or to baseline before the next cycle of treatment will be administered. If patients cannot be treated within 2 weeks of intended dosing, due to toxicity, they should be taken off treatment.

7. Treatment discontinuation

Patients will be removed from the study for the following reasons:

- Documented disease progression
- Persistent grade 2 neuropathy
- Grade 3 neuropathy lasting more than 7 days
- Grade 3 or 4 toxicities requiring more than two dose reductions, or delay of treatment for more than 5 weeks in arm A or 6 weeks in arm B from the beginning of the last cycle

- Patient's request, or consent withdrawal

8. Concomitant treatments

Biphosponates will be administered sequentially **after** the infusion of ixabepilone. Normal saline will be used to wash out the catheter and the vain before the administration of biphosphonates.

Prohibited treatments: Subjects must not continue or institute treatment with strong inhibitors of CYP3A4 72 hours prior to the initiation of study therapy until end of treatment with ixabepilone (e.g., ketoconazole, itraconazole, ritonavir, amprenavir, indinavir, nelfinavir, delavirdine, or voriconazole).

Restricted treatments: The effect of mild or moderate inhibitors (e.g., erythromycin, fluconazole, or verapamil) on exposure to ixabepilone has not been studied. Therefore, caution should be used when considering administration with mild or moderate CYP3A4 inhibitors during treatment with ixabepilone, and alternative therapeutic agents that do not inhibit CYP3A4 should be considered for co-administration with ixabepilone. Patients receiving CYP3A4 inhibitors during treatment with ixabepilone should be monitored more closely for acute toxicities (e.g., frequent monitoring of peripheral blood counts between cycles of ixabepilone).

9. Patient follow-up

Patients will be seen at the clinic every three months following the discontinuation of the treatment.

10. Data management and monitoring of study

Each of the 8 sites that will be involved in the study employs one or two full-time data managers responsible for following the randomization/registration procedures, according to the eligibility criteria. Randomization of the subjects will be done centrally at the HeCOG central office in Athens. Data managers ensure that the subject's medical file contains all source data and documents required. They are also delegated to completing and continuously updating the CRFs, entering the data in the HeCOG electronic database and handling the queries whenever necessary.
In addition, a full-time monitor will ensure that the investigators and the trial staff are adequately informed about the trial and will verify that they are performing the specified activities in accordance to the approved protocol and amendment(s), and that the reported trial data is accurate, complete and verifiable from source documents. The monitor also verifies that written informed consent was obtained before each subject's participation in the trial and ensures that all Adverse Events are reported within the time periods required. After each site visit, the monitor submits a written report of the observations and findings to the Investigators.

11. Planned timetable

Estimated date of first patient enrolled: March 2008
Estimated date of last patient completed: November 2009

12. Target patient population

Female patients aged 18 to 75 years inclusive, with histologically confirmed metastatic breast cancer that has been characterized HER-2 negative by standard techniques, will be enrolled. Patients must have previously been treated with chemotherapy in the adjuvant or neo-adjuvant setting. They must have a World Health Organization (WHO) performance status of 0 to 1 and a sufficient hepatic, renal and cardiac function. They must have sufficient bone marrow function and absence of peripheral neuropathy grade 2 or higher. Patients must have a life expectancy of > 3 months and sign a written informed consent.

13. Number of patients

A total of 150 patients (75 per study arm) will be enrolled in this study. With this sample size and an estimated response rate of 30% we'll be able to estimate response rates of the two arms with a 95% confidence interval of +/- 10%.

14. Investigational Treatment

Product Identification

Ixabepilone for injection is supplied as a lyophilized, white to off-white, whole or fragmented cake in a vial. The drug product is available as a 15 mg/vial. The vial containing vehicle for constitution of ixabepilone for injection, 8.0 mL/vial, will be supplied with the freeze-dried product. The vehicle is a mixture of dehydrated alcohol plus BMS-purified polyoxyethylated castor oil, which appears as a clear to slightly hazy, colorless to pale yellow solution. One vial of 8.0 mL/vial vehicle product is provided whenever a 15 mg/vial of ixabepilone for injection is supplied. Sites will be responsible for recording the container number of the supplies dispensed on drug accountability forms and CRFs. Vials that are provided by BMS will be labeled according to the respective country regulations and may contain information regarding the product strength, quantity, storage conditions and direction of use.

Handling and Dispensing

Administration of all investigational products must be according to the product label or as specified in the protocol.

The investigational product should be stored in a secure area according to local regulations. It is the responsibility of the investigator to ensure that the investigational product is only dispensed to study subjects. The investigational product must be dispensed only from official study sites by authorized personnel according to local regulations.

The investigator should ensure that the investigational product is stored in accordance with the environmental conditions (temperature, light, and humidity) as determined by the manufacturer.

Ixabepilone for injection should be stored refrigerated at 2°C to 8°C (36 to 46°F) and should be protected from light. The vehicle for constitution should be stored at 2°C to 8°C, or 2°C to 25°C, as directed on the label. If t he vehicle is refrigerated, it must be allowed to warm to room temperature before constitution of the lyophyle. After initial constitution with the accompanying vehicle, the solution may be stored in the vial for a maximum of one hour at room temperature and room light. The constituted solution should not be stored in the syringe. After final dilution with Lactated Ringer's Injection (LRI) to ixabepilone concentration between 0.2 mg/mL and 0.6 mg/mL, the solution is

stable when stored at room temperature and room light for a maximum of 6 hours.
Administration of the entire infusion volume must be completed within the 6-hour time period as noted above.
Prior to constitution of the lyophile, the vehicle should be kept at room temperature for approximately 1 hour. Using a suitable syringe, slowly inject the appropriate volume of vehicle into the vial of ixabepilone. Gently swirl the vial until the lyophile is completely dissolved. When completely dissolved the solution concentration of ixabepilone is 2 mg/mL. This solution must be further diluted with LRI to a final ixabepilone concentration ranging from 0.2 mg/mL to 0.6 mg/mL before administration to the subject. Withdraw the appropriate volume of the constituted solution containing 2 mg/mL of active drug, and transfer the constituted solution into the i.v. bag containing the appropriate volume of LRI to achieve the final desired concentration of ixabepilone. The infusion must be administered through an appropriate inline filter with a microporous membrane of 0.22 to 5.0 microns. LRI should be used to flush the i.v. line or extension set at the end of the infusion if flushing is required. Any remaining solution should be discarded according to the institutional procedures for cytotoxics.
Note 15 mg/vial: The label fill for the drug is 15 mg/vial lyophile, which is to be constituted to a concentration of 2 mg/mL with the vehicle. To account for via/needle/syringe (VNS) loss the actual amount of drug in the vial is 16 mg (± 3%).
Hence the drug should be constituted using 8.0 mL of the vehicle for constitution (to achieve a concentration of ixabepilone of 2 mg/mL).

Initial Orders and re-supply

Ixabepilone for injection and its vehicle for constitution (diluent) will be supplied by BMS. Both ixabepilone for injection and vehicle for constitution are packaged in Type I glass vials, stoppered with butyl rubber closures and sealed with aluminum seals. A sufficient excess of drug and vehicle is provided in the respective vials to allow for withdrawal losses. Sites will be responsible for recording the label batch number/lot number of the supplies dispensed on drug accountability forms and CRFs.

Ixabepilone for injection will be labelled as follows:
BMS-247550-01 for Injection
15 mg /vial
For Intravenous Use.
Reconstitute, dilute and administer as directed in the protocol.
Store at 2 - 8℃ (36 - 46℉)
Protect from light.
Batch No:

The **diluent** for ixabepilone for injection will be labeled as follows:
Diluent/Vehicle for Constitution for BMS-247550-01 Injection
(50% Cremophor®EL + 50% dehydrated ethanol, USP)
8.0 mL/ vial
For dilution only.
Store at 2 - 8℃ (36 - 46℉) **OR some may read as:**
Store at 2 - 25℃

In order to minimize subject exposure to the plasticizer di-(2-ethylhexyl) phthalate (DEHP) which may be leached from some brands of polyvinyl chloride (PVC) infusion bags or administration sets, diluted ixabepilone solutions should be stored in bottles (glass, polypropylene) or plastic bags (polyethylene, polypropylene, polyolefin, ethylene-vinyl-acetate) and administered through polyethylene-lined administration sets

plasticized with TOTM (trioctyl trimellitate). i.v. sets and components, including filters 0.20 to 0.22, typically used for the administration of paclitaxel, have been found to be compatible with infusions of BMS-247550. Lactated Ringers Injection (LRI) in non-DEHP Excel® bags is available from B. Braun McGaw, Inc., and can be used for preparing the infusion.
The following infusion components have been qualified for use with BMS-247550:

i.v. sets containing an in-line 0.22 micron filter
Baxter Vented Paclitaxel Set (Catalog # 2C7553)
Abbott Primary i.v. Plumset (Catalog #11947)

i.v. sets **not** containing an in-line 0.22 micron filter
• McGaw AccuPro Pump Nitroglycerin i.v. Set (Catalog # V8333)
• Clintec IV Fat Emulsion Set (Catalog # 2C1105)

Filter Extension Sets (to be used with i.v. sets not containing an in-line filter
Braun Filtered Extension Set with 5 Micron Filter (Catalog #FE-5010Y).
The infusion must be administered through an appropriate in-line filter with a microporous membrane of 0.20 to 5.0 microns.
Diluted ixabepilone solutions may also be administered using a syringe pump and polyethylene-lined extension sets.
Appropriate mask, protective clothing, eye protection, gloves and Class II vertical-laminar-airflow safety cabinets are recommended during preparation and handling.
Initial orders of ixabepilone will be requested to Creapharm Developpment SAS, Z.A. Airspace, Avenue de magudas, 33185 Le Haillan, France, fax number: +33 (0)5 57 92 46 50 , e-mail: micheletti@creapharm.fr upon screening of the first patient.
Drug re-supply request form should be submitted electronically or by fax to +33 (0)5 57 92 46 50 at least 5 business days before the expected delivery date. Deliveries will be made Tuesday through Friday.

Accountability

It is the responsibility of the Investigators to ensure that a current record of ixabepilone disposition is maintained at each study site where ixabepilone is inventoried and disposed. Records or logs must comply with applicable regulations and guidelines, and should include:
- Amount received and placed in storage area.
- Amount currently in storage area.
- Label ID number and expiry date.
- Dates and initials of person responsible for each ixabepilone inventory entry/movement.
- Amount dispensed to and returned by each subject, including unique subject identifiers.
- Amount transferred to another area/site for dispensing or storage.
- Non-study disposition (e.g., lost, wasted, broken).
- Amount returned, if applicable.
- Amount destroyed at study site, if applicable.

Ixabepilone dispensing record/inventory logs and copies of signed packing lists must be maintained at the investigational site. Batch numbers for ixabepilone must be recorded

in the drug accountability records.

Destruction

It is the Investigator's responsibility to ensure that arrangements have been made for disposal and that procedures for proper disposal have been established according to applicable regulations, guidelines, and institutional procedures. Appropriate records of the disposal must be maintained.

Destruction of supplies should only be carried out once any discrepancies have been investigated and satisfactorily explained, and the reconciliation has been accepted. In addition recording of destruction operations should be carried out in such a manner that all operations may be accounted for.

Study drug will be returned to BMS for destruction.

When destruction of drug takes place, a dated certificate of or a receipt for destruction should be provided to the sponsor. These documents should clearly identify, or allow traceability to, the batches and/or patient numbers involved and the actual quantities destroyed.

V. TRANSLATIONAL RESEARCH STUDIES

The following translational research studies will be conducted in conjunction with this trial.

A. PHARMACOGENOMICS

Genetic polymorphisms in drug-metabolizing enzymes and drug transporters, such as the CYP3A and ABCB1 genes, will be explored in blood DNA (150 patients) and their association with the risk to develop treatment-related peripheral neuropathy and neutropenia will be assessed in each treatment arm. Patients will have a thorough clinical examination, which will include sensory and motor testing of the extremities, with assessment of proprioception, vibration, sharp/dull discrimination, and deep tendon reflexes. Functional testing will include balance testing (BT) and dexterity and will be evaluated by the Jebsen-Taylor test of hand function (JTHF).

B. PREDICTIVE BIOMARKERS FOR RESPONSE

Formalin-fixed paraffin-embedded (FFPE) tumor samples of the primary tumors of all enrolled patients (150 patients) and, if available, of metastatic lesions prior to treatment initiation will be assessed for biomarkers of a potentially predictive value. Tumor tissues will be assessed by immunohistochemistry (IHC) for tubulin stabilization, acetylated alpha-tubulin, beta-3-microtubulin, Tau-1, p53, Bcl2, Bax, transforming acid coiled coil-3 (TACC3), and multidrug transporters at the protein level. Alpha- and beta-tubulin genes will also be assessed by quantitative real-time PCR at the RNA level and for mutations, in FFPE tumor samples, because microtubule-stabilization has been shown to confer resistance to microtubule-targeting drugs.[43-46]

C. STUDY OF ANTI-ANGIOGENIC EFFECTS

Baseline blood samples will be collected prior to treatment initiation from all patients

using EDTA as an anticoagulant. Samples will also be collected 3 and 6 weeks after treatment initiation to study the effects of each dosing schedule on kinetics of soluble biomarkers. All samples will be centrifuged for 10 minutes at 2000 rpm within 5 minutes of collection. The plasma will be separated immediately and 4 aliquot samples of 0.5 ml will be stored at -80℃ for a maximum of 24 months for ELISA determination of circulating VEGF, FGFb and TSP1 levels. A total of 450 samples will be assessed (150 patients X 3 collection time points). In addition, the possible predictive value of the blood levels of the above angiogenic factors on PFS and OS will be assessed.
The time-plan for the translational research studies is given in the table in Section VII.

VI. CLINICAL ASSESSMENTS

A. EFFICACY

Measurable disease will be assessed by imaging studies, preferably CT scans, during the duration of the treatment using the RECIST method [Appendix A].

Patients must undergo baseline imaging during the 2 weeks preceding treatment initiation, and will be scanned repeatedly 8, 16 and 24 weeks after the initiation of the treatment and every 3 months thereafter. Response evaluation will be done centrally by two independent radiologists.

B. TOXICITY

Toxicities will be assessed using NCI Common Toxicity Criteria version 3 at baseline and at the conclusion of each cycle of treatment. [Appendix D]. All adverse events will be recorded in Case Report Forms [CRFs].

1. Serious adverse events

Serious adverse events (SAEs) must be reported within 24hours by direct telephone and fax contacts to the HeCOG central Office and one of the Principal Investigators.
A ***serious adverse event*** or reaction is any untoward medical occurrence that at <u>any dose</u>:

- results in death
- is life-threatening (defined as an event in which the patient or subject was at risk of death at the time of the event; it does not refer to an event which hypothetically might have caused death if it were more severe) or requires inpatient hospitalization or prolongation of existing hospitalization (refer to note for exceptions)
- results in persistent or significant disability/incapacity
- is a congenital anomaly/birth defect
- results in the development of drug dependency or drug abuse
- is an important medical event [defined as a medical event that may not be immediately life-threatening or result in death or hospitalization but, based upon appropriate medical and scientific judgment, may jeopardize the patient/subject or may require intervention (e.g., medical, surgical) to prevent one of the other serious outcomes listed in the definition above]

Exceptions to the 2nd SAE:

- Planned hospitalization for a medical/surgical procedure
- Routine health assessment requiring admission for baseline/trending of health status documentation (e.g.: routine colonoscopy)
- Medical/surgical admission for purpose other than remedying ill health state (planned prior to entry in study trial; appropriate documentation required)
- Admission encountered for other life circumstance that carries no bearing on health status and requires no medical/surgical intervention (e.g. lack of housing, economic inadequacy, care-giver respite, family circumstances, administrative)

For reporting purposes, occurrences of pregnancy or overdose (regardless of adverse outcome) should be considered as events, which must be reported as important medical events. An overdose is defined as the accidental or intentional administration of any dose of a product that is considered both excessive and medically important.

Serious adverse events must be thoroughly investigated and the results must be kept available in patient's medical records and on data collection documents.

a) Reporting of SAEs

Following the subject's written consent to participate in the study, all SAEs should be collected and reported, including those thought to be associated with clinical trial procedures. Following study completion, any SAE thought to be related to study drug or clinical trial procedures should also be reported to BMS.

SAE terminology and severity grading will be based on *CTCAEv3*.

The following categories and definitions of causal relationship to study drug should be considered for use for all clinical studies supported by BMS:

- Certain: There is a known causal relationship between the study drug and the SAE. The event responds to withdrawal of study drug (de-challenge), and recurs with re-challenge when clinically feasible. (>95% certainty)
- Probable: There is reasonable causal relationship between the study drug and the SAE. The event responds to de-challenge. Re-challenge is not required. (65%-95% probability)
- Possible: There is reasonable causal relationship between the study drug and the SAE. De-challenge information is lacking or unclear. (35%-65% probability of relatedness)
- Not likely: There is temporal relationship to study drug administration, but there is not a reasonable causal relationship between the study drug and the SAE. (5-35% probability of relatedness)
- Not related: There is not a temporal relationship to study drug administration (too early or late, or study drug not taken), or there is known causal relationship between the SAE and another drug, concurrent disease, or other circumstance. (<5% chance of relatedness)

Handling of Expedited Safety Reports

In accordance with local regulations, the sponsor will notify investigators of all SAEs that are unexpected (ie, not previously described in the Investigator Brochure), and certainly, probably, or possibly related to the investigational product or that could be associated

with the study procedures. This notification will be in the form of an expedited safety report (ESR).

Upon receiving such notices, the investigator must review and retain the notice with the Investigator Brochure. Where required by local regulations or when there is a central IRB/IEC for the study, the sponsor will submit the ESR to the appropriate IRB/IEC. The investigator and IRB/IEC will determine if the informed consent requires revision. The investigator should also comply with the IRB/IEC procedures for reporting any other safety information. Where required, submission of ESRs by the investigator to Health Authorities should be handled according to local regulations.

2. Toxic deaths

Toxic death is defined as death due to the study treatment.

3. Evaluation of toxicity

All patients who have started the treatment will be included in the overall toxicity analyses.
Patients who have discontinued treatment because of toxicity will always be included in the toxicity analyses. Toxicity and adverse events occurring in ineligible patients will be reported separately.

VII. EVALUATION AND VISIT SCHEDULE

Evaluation	Screening Baseline	Cycle 1	Cycle 2	Cycle 3	Cycle 4	Cycle 5	Cycle 6	Cycle 7	Cycle 8	Follow up (every 3 months)
Selection (inclusion/exclusion criteria), medical history, demography, signed informed consent	x									
Arm A Physical exam, PS	x	Week 1	Week 1	Week 1	Week 1	Week 1	Week 1	Week 1	Week 1	x
Arm B Physical exam, PS	x	Weeks 1-3	Weeks 1-3	Weeks 1-3	Weeks 1-3	Weeks 1-3	Weeks 1-3			x
Laboratory: hematology	x	weekly	weekly	weekly	weekly	weekly	weekly	weekly[a]	weekly[a]	x
Lab: serum biochemistry, urinalysis	x	x	x	x	x	x	x	x[a]	x[a]	x
BSA + Pregnancy test	x	x	x	x	x	x	x	x[a]	x[a]	
EKG	x									
Concomitant medications	x	x	x	x	x	x	x	x	x	x
Adverse events, toxicity		x	x	x	x	x	x	x	x	x
Arm A: Evaluation/staging (Radiographic imaging)	x			8 weeks			16 weeks		24 weeks	
Arm B: Evaluation/staging (Radiographic imaging)	x		8 weeks		16 weeks		24 weeks			
Neurological exams	x			x [b]	X [a]		x [b]		x [a]	
Tissue Sampling for Translational Res.	Prim/Met									
Blood Sampling for Translational Res.	x	3weeks	6 weeks							

[a] arm A only Prim = Primary tumor
[b] arm B only Met = Metastatic lesion, if available

VIII. STATISTICAL DESIGN AND METHODS

A. STATISTICAL DESIGN

With a fixed sample size of 150 patients (75 patients in each treatment arm), α=5% significance level and an expected objective response rate around 30%, the maximum width of the confidence interval (using the normal approximation) of the response rate will be 2 X 10% (i.e. 20%).
The study accrual is estimated at 10 patients per month, leading to a total accrual time of 15 months. Response evaluation will be determined at 8, 16 and 24 weeks of treatment, therefore the total study duration is estimated at 21 months, a which time the final analysis is expected to be performed.

B. DEFINITIONS

Enrolled patients
Any subject who signed an informed consent form.
Randomized patients
Any patient randomized in one of the 2 arms of the study.
Treated patients
Any patient that received at least 1 dose of the investigational product.
Response-evaluable patients
Any patient with measurable disease included in the "randomized patients" group. That would include all randomized patients, since measurable disease is one of the inclusion criteria.
Definition of Best overall response
It is the best response recorded from the day of randomization until disease progression. (See also Appendix A, page 38).
Definition of Time to Response
A subject's time to response is defined as the time in days from randomization until measurement criteria are first met for a PR or CR (whichever is recorded first). Time to response is only computed for subjects whose best overall response is PR or CR.
Definition of Duration of Response
A subject's duration of response is defined as the period measured in months from the time that measurement criteria are first met for CR or PR (whichever is recorded first) until the first date of documented progressive disease or death from any cause without prior documentation of progression. Subjects who neither relapse, nor die, will be censored on the date of their last tumor assessment. Duration of response is only computed for subjects whose best overall response is PR or CR.
Definition of Progression-Free Survival
A subject's progression-free survival (PFS) time is defined as the time in months from randomization until the first date of documented progressive disease (PD) or death from any cause without prior documentation of progression. Subjects who do not progress or die, will be censored on the date of their last tumor assessment.
Definition of Time to Treatment Failure
A subject's Time to Treatment Failure (TTF) is defined as the time in months from randomization until the date of discontinuation of treatment for any reason, including disease progression, treatment toxicity, and death. Subjects who do not progress, die or discontinue treatment due to toxicity or any other reason, will be censored on the date of their last tumor assessment.

Definition of Overall Survival
Overall survival is defined as the time in months from randomization until the time of death. Subjects who have not died or who are lost to follow-up will be censored on the last date on which the subject is known to be alive.

C. ANALYSES

Demographic data, medical history and baseline conditions will be summarized using descriptive statistics for all randomized patients
Summary tables of dosing and safety parameters will be presented for all treated patients.
Efficacy analyses will include all randomized patients.
All patients randomized in the study will be assessed for response to treatment, even if there are minor protocol treatment deviations. Each patient will be assigned to one of the following categories: 1) complete response, 2) partial response, 3) stable disease, 4) progressive disease, 5) early death from malignant disease, 6) early death from toxicity, 7) early death because of other cause, or 8) unknown (not assessable, insufficient data). Patients in response categories 4-8 should be considered as failing to respond to treatment (disease progression).
Best overall response will be determined (centralized review of the scans) for each treatment arm. All time to event endpoints will be estimated using the Kaplan-Meier product-limit method. Medians with corresponding 95% confidence intervals will be used to summarize survival in the two groups.
For the translational research studies, the association between genetic polymorphisms in the CYP3A or ABCB1 genes and the development of treatment related peripheral neuropathy or neutropenia would be assessed with the use of Fisher's exact test. The possible predictive value of the IHC and PCR biomarkers on PFS and OS will be evaluated with the use of both univariate and multivariate Cox regression analysis. The same methods will be used in order to assess for possible predictive value of blood levels of angiogenic factors on PFS and OS. Finally, repeated measures analysis of variance (ANOVA) will be used to test for within and between arm differences regarding the effect of each dosing schedule on blood levels of angiogenic factors at different time-points (baseline, 3 and 6 weeks).

IX. REFERENCES

1. Jemal A, Siegel R, Ward E et al. Cancer statistics, 2006. CA Cancer J Clin 2006; 56(2):106-130.
2. Bonadonna G, Brusamolino E, Valagussa P et al. Combination chemotherapy as an adjuvant treatment in operable breast cancer. N Engl J Med 1976; 294(8):405-410.
3. Jenks S. Benefits of adjuvant therapy for breast cancer reaffirmed. J Natl Cancer Inst 1992; 84(3):144.
4. Polychemotherapy for early breast cancer: an overview of the randomised trials. Early Breast Cancer Trialists' Collaborative Group. Lancet 1998; 352(9132):930-942.
5. Fountzilas G, Papadimitriou C, Aravantinos G et al. Dose-dense sequential adjuvant chemotherapy with epirubicin, paclitaxel and CMF in high-risk breast cancer. Oncology 2001; 60(3):214-220.
6. Henderson IC, Berry DA, Demetri GD et al. Improved outcomes from adding sequential Paclitaxel but not from escalating Doxorubicin dose in an adjuvant chemotherapy regimen for patients with node-positive primary breast cancer. J Clin Oncol 2003; 21(6):976-983.
7. Mamounas EP, Bryant J, Lembersky B et al. Paclitaxel after doxorubicin plus cyclophosphamide as adjuvant chemotherapy for node-positive breast cancer: results from NSABP B-28. J Clin Oncol 2005; 23(16):3686-3696.
8. Martin M, Pienkowski T, Mackey J et al. Adjuvant docetaxel for node-positive breast cancer. N Engl J Med 2005; 352(22):2302-2313.
9. Roche H, Fumoleau P, Spielmann M et al. Sequential adjuvant epirubicin-based and docetaxel chemotherapy for node-positive breast cancer patients: the FNCLCC PACS 01 Trial. J Clin Oncol 2006; 24(36):5664-5671.
10. Bria E, Nistico C, Cuppone F et al. Benefit of taxanes as adjuvant chemotherapy for early breast cancer: pooled analysis of 15,500 patients. Cancer 2006; 106(11):2337-2344.
11. Jones SE, Savin MA, Holmes FA et al. Phase III trial comparing doxorubicin plus cyclophosphamide with docetaxel plus cyclophosphamide as adjuvant therapy for operable breast cancer. J Clin Oncol 2006; 24(34):5381-5387.
12. Dang C, Hudis C. Adjuvant taxanes in the treatment of breast cancer: no longer at the tip of the iceberg. Clin Breast Cancer 2006; 7(1):51-58.
13. Bollag DM, McQueney PA, Zhu J et al. Epothilones, a new class of microtubule-stabilizing agents with a taxol-like mechanism of action. Cancer Res 1995; 55(11):2325-2333.
14. Altmann KH. Epothilone B and its analogs - a new family of anticancer agents. Mini Rev Med Chem 2003; 3(2):149-158.
15. Lee FY, Borzilleri R, Fairchild CR et al. BMS-247550: a novel epothilone analog with a mode of action similar to paclitaxel but possessing superior antitumor efficacy. Clin Cancer Res 2001; 7(5):1429-1437.
16. Yamaguchi H, Paranawithana SR, Lee MW, Huang Z, Bhalla KN, Wang HG. Epothilone B analogue (BMS-247550)-mediated cytotoxicity through induction of Bax conformational change in human breast cancer cells. Cancer Res 2002; 62(2):466-471.
17. Hoffmann J, Buchmann B, Schwede W et al. New synthetic Epothilone Derivative ZK-EPO inhibits breast cancer metastasis. AACR Meeting Abstracts 2005; 2005(1):807-80b.

18. Denduluri N, Low JA, Lee JJ, Berman AW, Walshe JM et al. Phase II Trial of Ixabepilone, an Epothilone B Analog, in Patients With Metastatic Breast Cancer Previously Untreated With Taxanes. J Clin Oncol 2007; epub ahead of print on July 2nd, 2007; 25 (23).

19. Conte P, Thomas E, Martin M, Klimovsky J, Tabernero J. Phase II Clinical Trial of Ixabepilone (BMS-247550), an Epothilone B Analog, in Patients With Taxane-Resistant Metastatic Breast Cancer. J Clin Oncol 2007; epub ahead of print on July 2nd, 2007; 25 (23).

20. Low JA, Wedam SB, Lee JJ et al. Phase II clinical trial of ixabepilone (BMS-247550), an epothilone B analog, in metastatic and locally advanced breast cancer. J Clin Oncol 2005; 23(12):2726-2734.

21. Oconnor O, Strauss D, Moskwitz C et al. Targeting the microtubule apparatus in indolent and mantle cell lymphoma with the novel epothilone analog BMS247550 induces a major and durable remissions in drug resistant disease. ASCO 2005, Poster discussion.

22. Pavlick AC, Millward M, Farrell K Broseus A et al, A phase II study of epothilone B analogue BMS 247550 in stage IV malignant melanoma, 2004 ASCO poster presentation (NSC#710428).

23. Liu G, Wang W, Dipaola R et al. A phase II study of weekly schedule of BMS- 247550 for patients with Hormonal refractory prostate Cancer: A trial of the Eastern Cooperative Oncology Group (E3803) ASCO Abstract submitted for 2006 annual meeting.

24. B Burtness, M Goldwasser, R Axelrod, et al. Randomized phase II study of BMS-247550 (NSC 710428) given daily X 5 days or weekly in patients with metastatic or recurrent squamous cell cancer of the head and neck: E2301. ASCO 2006.

25. Phase II Clinical Trial of Ixabepilone (BMS-247550), an Epothilone B Analog, As First-Line Therapy in Patients With Metastatic Breast Cancer Previously Treated With Anthracycline Chemotherapy
Henri Roché, Louise Yelle, Francesco Cognetti, Louis Mauriac, Craig Bunnell, Joseph Sparano, Pierre Kerbrat, Jean-Pierre Delord, Linda Vahdat, Ronald Peck, David Lebwohl, Rana Ezzeddine, Hervé Curé. JCO Jul 2 2007: doi:10.1200/JCO.2006.09.7535

26. Fountzilas G, Tsavdaridis D, Kalogera-Fountzila A et al. Weekly paclitaxel as first-line chemotherapy and trastuzumab in patients with advanced breast cancer. A Hellenic Cooperative Oncology Group phase II study. Ann Oncol 2001; 12(11):1545-1551.

27. Briasoulis E, Pentheroudakis G, Karavasilis V, Tzamakou E, Rammou D, Pavlidis N. Weekly paclitaxel combined with pegylated liposomal doxorubicin (CaelyxTM) given every 4 weeks: dose-finding and pharmacokinetic study in patients with advanced solid tumors. Ann Oncol 2004; 15(10):1566-1573.

28. Green MC, Buzdar AU, Smith T et al. Weekly paclitaxel improves pathologic complete remission in operable breast cancer when compared with paclitaxel once every 3 weeks. J Clin Oncol 2005; 23(25):5983-5992.

29. Lee H, Xu L, Wu S et al. Predictive biomarker discovery and validation for the targeted chemotherapeutic ixabepilone. J Clin Oncol (Meeting Abstracts) 2006; 24(18_suppl):3011.

30. Furstenberger G, von MR, Senn HJ, Boneberg EM. Real-time PCR of CD146 mRNA in peripheral blood enables the relative quantification of circulating endothelial cells and is an indicator of angiogenesis. Br J Cancer 2005; 93(7):793-798.

31. Schneider M, Tjwa M, Carmeliet P. A surrogate marker to monitor

angiogenesis at last. Cancer Cell 2005; 7(1):3-4.

32. Low JA, Wedam SB, Brufsky A et al. A phase 2 trial of BMS-247550 (ixabepilone), an epothilone B analog, given daily x 5 in breast cancer. J Clin Oncol (Meeting Abstracts) 2004; 22(14_suppl):545.

33. Bertolini F, Paul S, Mancuso P et al. Maximum tolerable dose and low-dose metronomic chemotherapy have opposite effects on the mobilization and viability of circulating endothelial progenitor cells. Cancer Res 2003; 63(15):4342-4346.

34. Lee JJ, Low JA, Croarkin E et al. Changes in neurologic function tests may predict neurotoxicity caused by ixabepilone. J Clin Oncol 2006; 24(13):2084-2091.

35. de JM, Verweij J. The epothilone dilemma. J Clin Oncol 2005; 23(36):9048-9050.

36. Freilich RJ, Balmaceda C, Seidman AD, Rubin M, DeAngelis LM. Motor neuropathy due to docetaxel and paclitaxel. Neurology 1996; 47(1):115-118.

37. Mielke S, Sparreboom A, Steinberg SM et al. Association of Paclitaxel pharmacokinetics with the development of peripheral neuropathy in patients with advanced cancer. Clin Cancer Res 2005; 11(13):4843-4850.

38. Mielke S, Sparreboom A, Mross K. Peripheral neuropathy: a persisting challenge in paclitaxel-based regimes. Eur J Cancer 2006; 42(1):24-30.

39. Shimizu T, Yamamoto N, Fujisaka Y et al. Phase I study of BMS-247550 (ixabepilone) given every 3 weeks in Japanese patients with refractory solid tumors. J Clin Oncol (Meeting Abstracts) 2005; 23(16_suppl):2050.

40. Aghajanian C, O'Connor O, Cohen M, Peck R, Burris H. Final results from a phase I trial of ixabepilone in patients with advanced malignancies and lymphoma. J Clin Oncol (Meeting Abstracts) 2006; 24(18_suppl):2039.

41. Mani S, McDaid H, Hamilton A et al. Phase I clinical and pharmacokinetic study of BMS-247550, a novel derivative of epothilone B, in solid tumors. Clin Cancer Res 2004; 10(4):1289-1298.

42. Dickson N, Peck R, Wu C, Burris H. Ixabepilone given weekly in patients with advanced malignancies: Final efficacy and safety results of a phase I trial. J Clin Oncol (Meeting Abstracts) 2006; 24(18_suppl):2040.

43. Don S, Verrills NM, Liaw TY et al. Neuronal-associated microtubule proteins class III beta-tubulin and MAP2c in neuroblastoma: role in resistance to microtubule-targeted drugs. Mol Cancer Ther 2004; 3(9):1137-1146.

44. Ganesh T, Yang C, Norris A et al. Evaluation of the tubulin-bound paclitaxel conformation: synthesis, biology, and SAR studies of C-4 to C-3' bridged paclitaxel analogues. J Med Chem 2007; 50(4):713-725.

45. Poruchynsky MS, Kim JH, Nogales E et al. Tumor cells resistant to a microtubule-depolymerizing hemiasterlin analogue, HTI-286, have mutations in alpha- or beta-tubulin and increased microtubule stability. Biochemistry 2004; 43(44):13944-13954.

46. Yang CP, Verdier-Pinard P, Wang F et al. A highly epothilone B-resistant A549 cell line with mutations in tubulin that confer drug dependence. Mol Cancer Ther 2005; 4(6):987-995.

X. ETHICS AND GENERAL TRIAL CONDUCTION ASPECTS

A. ETHICAL ASPECTS

1. General Issues

The investigators will ensure that this study is conducted in full conformance with the principles of the “Declaration of Helsinki” or with the laws and regulations of the country in which the research is conducted, whichever affords the greatest protection to the individual. The study must fully adhere to the principles outlined in the “Guideline for Good Clinical Practice” ICH Tripartite Guideline (January 1997) or with local law if it affords greater protection to the patient. [Appendix B]

2. Informed Consent

It is the responsibility of the investigators, or a person designated by the investigators to obtain written informed consent from each patient participating in this study, after adequate explanation of the aims, methods, anticipated benefits, and potential hazards of the study. [Appendix D] For patients not qualified or incapable of giving legal consent, written consent must be obtained from a legally acceptable representative. In the case where both the patient and his/her legally acceptable representative are unable to read, an impartial witness should be present during the entire informed consent discussion. After the patient and representative have orally consented to participation in the trial, the witness’ signature on the form will attest that the information in the consent form was accurately explained and understood. The investigator or designee must also explain that the patients are completely free to refuse to enter the study or to withdraw from it at any time, for any reason. The Case Report Forms [CRF] for this study contain a section for documenting informed patient consent, and this must be completed appropriately. If new safety information results in significant changes in the risk/benefit assessment, the consent form should be reviewed and updated if necessary. All patients (including those already being treated) should be informed of the new information, given a copy of the revised form and give their consent to continue in the study.

3. Ethics Committees

This protocol and patient information sheet will be submitted by the investigator to the National Ethics Committee on Clinical Trials. Approval from the committee will be obtained before starting the study, and should be documented in a letter to the investigator specifying the date on which the committee met and granted the approval.

Any modifications made to the protocol after receipt of the Ethics Committee approval must also be submitted by the investigator to the Committee, in accordance with local procedures and regulatory requirements.

4. Conditions for Modifying The Protocol

All protocol modifications must be submitted to the appropriate Independent Ethics Committee or Institutional Review Board for information and approval in accordance

with local requirements, and to Regulatory Agencies if required. Approval must be awaited before any changes can be implemented, except for changes necessary to eliminate an immediate hazard to trial patients, or when the change(s) involves only logistical or administrative aspects of the trial (e.g. change in monitor(s), change of telephone number(s)).

5. Conditions for Terminating the Study

Both the Sponsor and the investigator reserve the right to terminate the study at any time. Should this be necessary, both parties will arrange the procedures on an individual study basis after review and consultation. In terminating the study, Sponsor and the investigator will assure that adequate consideration is given to the protection of the patient's interests.

B. STUDY DOCUMENTATION AND RECORD KEEPING

1. Investigator's Files / Retention of Documents

The Investigator must maintain adequate and accurate records to enable the conduct of the study to be fully documented and the study data to be subsequently verified. These documents should be classified into two different separate categories (1) Investigator's Study File, and (2) Patient clinical source documents.

The Investigator's Study File will contain the protocol/amendments, Case Report and Query Forms, Independent Ethics Committee/Institutional Review Board and governmental approval with correspondence, sample informed consent, drug records, staff curriculum vitae and authorization forms and other appropriate documents/correspondence etc.

Patient clinical source documents (usually defined by the project in advance to record key efficacy/safety parameters independent of the CRFs) would include patient hospital/clinic records, physician's and nurse's notes, appointment book, original laboratory reports, ECG, EEG, Xray, pathology and special assessment reports, signed informed consent forms, consultant letters, and patient screening and enrolment logs. The Investigator must keep these two categories of documents on file for at least 15 years after completion or discontinuation of the study. After that period of time the documents may be destroyed, subject to local regulations.

2. Source Documents and Background Data

The investigator shall supply the sponsor on request with any required background data from the study documentation or clinic records. This is particularly important when Case Report Forms are illegible or when errors in data transcription are suspected. In case of special problems and/or governmental queries or requests for audit inspections, it is also necessary to have access to the complete study records, provided that patient confidentiality is protected.

3. Audits and Inspections

The investigators should understand that source documents for this trial should be made available to authorized trial-monitors or health authority inspectors after

appropriate notification. The verification of the Case Report Form data must be made by direct inspection of source documents.

4. Case Report Forms – Data Flow

a) CRFs

For each patient enrolled, a Case Report Form must be completed and signed by the principal investigator or an authorized delegate from the study staff. This also applies to records for those patients who fail to complete the study (even during the pre-randomization screening period if a Case Report Form was initiated). If a patient withdraws from the study, the reason must be noted on the Case Report Form. If a patient is withdrawn from the study because of a treatment-limiting adverse event, thorough efforts should be made to clearly document the outcome.

All forms should be typed or filled out using indelible ink, and must be legible. Errors should be crossed out but not obliterated, the correction inserted, and the change initialled and dated by the investigator or his/her authorized delegate. The investigator should ensure the accuracy, completeness, legibility, and timeliness of the data reported to the sponsor in the CRFs and in all required reports.

b) Data Flow

The CRFs must be completed and signed by the investigator or one of his/her authorized staff members as soon as the requested information is available, according to the above-described schedule. The list of staff members authorized to sign CRFs must be sent to the HeCOG Data Center by the responsible investigators before the start of the study.

In all cases, it remains the responsibility of the investigator to check that CRFs are sent to the HeCOG Data Center, and that they are completely and correctly filled out. A copy of the CRF will be sent to the HeCOG data Center as the initial form, with the investigator keeping the original copy until the study is completed or all queries are satisfactorily answered. The original will then be requested by and must be sent to the HeCOG data center.

The HeCOG Data Center will perform extensive consistency checks on the CRFs and issue Query Forms in case of inconsistent data. Query Forms must be answered immediately and signed by the investigator (or an authorized staff member). Corrections should be made to the original CRF (held by the investigator) and a copy of the corrected CRF must be sent with the Query response.

If an investigator (or an authorized staff member) needs to modify a CRF after the original copy has been returned to the HeCOG Data Center, he/she should notify the Data Center in writing with a copy of the changes.

It will be the monitor's responsibility to inspect the Case Report Forms at regular intervals throughout the study, to verify the adherence to the protocol and the completeness, consistency and accuracy of the data being entered on them. The monitor should have access to laboratory test reports and other patient records needed to verify the entries on the Case Report Form. The investigator (or his/her deputy) agrees to cooperate with the monitor to ensure that any problems detected in the course of these monitoring visits are resolved.

5. Confidentiality of Trial Documents And Patient Records

The investigator must assure that patients' anonymity will be maintained and that their identities are protected from unauthorized parties. On CRFs or other documents submitted to the sponsor, patients should not be identified by their names, but by an identification code. The investigator should keep a patient enrolment log showing codes, names and addresses. The investigator should maintain documents not for submission to BMS, e.g., patients' written consent forms, in strict confidence.

6. Publication of Data and Protection Of Trade Secrets

The results of this study may be published or presented at scientific meetings. The final publication of the trial results will be written by the Principal Investigator in collaboration with the Study Co-ordinators on the basis of the data analysis performed at the HeCOG Data Centre. A draft manuscript will be completed no later than 6 months after the last patient has discontinued therapy. After revision by the co-authors and BMS, this manuscript will be sent to a major scientific journal.

The authorship of any publication will include all investigators who have enrolled more than 5% of the eligible study patients, and any individuals who have significantly contributed to the inception, implementation, monitoring, or interpretation of the study.

The investigator agrees to submit all manuscripts or abstracts to BMS prior to submission at least two weeks prior to submission for abstracts, and six weeks for manuscripts or slides for presentation. This allows the BMS to protect proprietary information and to provide comments based on information from other studies that may not yet be available to the investigator.

All manuscripts will include an appropriate acknowledgement section, mentioning all investigators who have contributed to the trial, as well as supporting bodies.

C. ADMINISTRATIVE RESPONSIBILITIES

The Principal Investigators will be responsible for writing the protocol, reviewing all CRFs and documenting their review on evaluation forms, discussing the contents of the reports with the Data Manager and/or the Statistician, and publishing the study results. They will also be generally responsible for answering all clinical questions concerning eligibility, treatment, and evaluation of the patients.

XI. APPENDICES

A. RESPONSE EVALUATION CRITERIA IN SOLID TUMORS

Response Evaluation Criteria in Solid Tumors (RECIST)
Quick Reference (http://ctep.cancer.gov/guidelines/recist.html):
Eligibility
Only patients with measurable disease at baseline will be included

- **MEASURABLE DISEASE** - the presence of at least one measurable lesion. If the measurable disease is restricted to a solitary lesion, its neoplastic nature should be confirmed by cytology/histology.
- **MEASURABLE LESIONS** - lesions that can be accurately measured in at least one dimension with longest diameter ≥ 20 mm using conventional techniques or ≥ 10 mm with spiral CT scan.
- **NON-MEASURABLE LESIONS** - all other lesions, including small lesions (longest diameter <20 mm with conventional techniques or <10 mm with spiral CT scan), i.e., bone lesions, leptomeningeal disease, ascites, pleural/pericardial effusion, inflammatory breast disease, lymphangitis cutis/pulmonis, cystic lesions, and also abdominal masses that are not confirmed and followed by imaging techniques; and.

All measurements should be taken and recorded in metric notation, using a ruler or calipers. All baseline evaluations should be performed as closely as possible to the beginning of treatment and never more than 4 weeks before the beginning of the treatment.
The same method of assessment and the same technique should be used to characterize each identified and reported lesion at baseline and during follow-up.
Clinical lesions will only be considered measurable when they are superficial (e.g., skin nodules and palpable lymph nodes). For the case of skin lesions, documentation by color-photography, including a ruler to estimate the size of the lesion, is recommended.
Methods of Measurement
CT and MRI are the best currently available and reproducible methods to measure target lesions selected for response assessment. Conventional CT and MRI should be performed with cuts of 10 mm or less in slice thickness contiguously. Spiral CT should be performed using a 5 mm contiguous reconstruction algorithm. This applies to tumors of the chest, abdomen and pelvis. Head and neck tumors and those of extremities usually require specific protocols.
Lesions on chest X-ray are acceptable as measurable lesions when they are clearly defined and surrounded by aerated lung. However, CT is preferable.
When the primary endpoint of the study is objective response evaluation, ultrasound (US) should not be used to measure tumor lesions. It is, however, a possible alternative to clinical measurements of superficial palpable lymph nodes, subcutaneous lesions and thyroid nodules. US might also be useful to confirm the complete disappearance of superficial lesions usually assessed by clinical examination.
The utilization of endoscopy and laparoscopy for objective tumor evaluation has not yet been fully and widely validated. Their uses in this specific context require sophisticated equipment and a high level of expertise that may only be available in some centers. Therefore, the utilization of such techniques for objective tumor response should be restricted to validation purposes in specialized centers. However, such techniques can be useful in confirming complete pathological response when biopsies are obtained.

Tumor markers alone cannot be used to assess response. If markers are initially above the upper normal limit, they must normalize for a patient to be considered in complete clinical response when all lesions have disappeared.
Cytology and histology can be used to differentiate between PR and CR in rare cases (e.g., after treatment to differentiate between residual benign lesions and residual malignant lesions in tumor types such as germ cell tumors).

Baseline documentation of "Target" and "Non-Target" lesions

All measurable lesions up to a maximum of five lesions per organ and 10 lesions in total, representative of all involved organs should be identified as ***target lesions*** and recorded and measured at baseline.
Target lesions should be selected on the basis of their size (lesions with the longest diameter) and their suitability for accurate repeated measurements (either by imaging techniques or clinically).
A sum of the longest diameter (LD) for *all target lesions* will be calculated and reported as the baseline sum LD. The baseline sum LD will be used as reference by which to characterize the objective tumor.
All other lesions (or sites of disease) should be identified as ***non-target lesions*** and should also be recorded at baseline. Measurements of these lesions are not required, but the presence or absence of each should be noted throughout follow-up.

RESPONSE CRITERIA

Response Evaluation of target lesions

Complete Response (CR):	Disappearance of all target lesions
Partial Response (PR):	At least a 30% decrease in the sum of the LD of target lesions, taking as reference the baseline sum LD
Progressive Disease (PD):	At least a 20% increase in the sum of the LD of target lesions, taking as reference the smallest sum LD recorded since the treatment started or the appearance of one or more new lesions
Stable Disease (SD):	Neither sufficient shrinkage to qualify for PR nor sufficient increase to qualify for PD, taking as reference the smallest sum LD since the treatment started

Response Evaluation of non-target lesions

Complete Response (CR):	Disappearance of all non-target lesions and normalization of tumor marker level.
Incomplete Response/ Stable Disease (SD):	Persistence of one or more non-target lesion(s) or/and maintenance of tumor marker level above the normal limits
Progressive Disease (PD):	Appearance of one or more new lesions and/or unequivocal progression of existing non-target lesions (1)

(1) Although a clear progression of "non target" lesions only is exceptional, in such circumstances, the opinion of the treating physician should prevail and the progression status should be confirmed later on by the review panel (or study chair).

Evaluation of best overall response

The best overall response is the best response recorded from the start of the treatment until disease progression/recurrence (taking as reference for PD the smallest measurements recorded since the treatment started). In general, the patient's best response assignment will depend on the achievement of both measurement and confirmation criteria.

Target lesions	Non-Target lesions	New Lesions	Overall response
CR	CR	No	CR
CR	Incomplete response/SD	No	PR
PR	Non-PD	No	PR
SD	Non-PD	No	SD
PD	Any	Yes or No	PD
Any	PD	Yes or No	PD
Any	Any	Yes	PD

Patients with a global deterioration of health status requiring discontinuation of treatment without objective evidence of disease progression at that time should be classified as having "symptomatic deterioration". Every effort should be made to document the objective progression even after discontinuation of treatment.

In some circumstances it may be difficult to distinguish residual disease from normal tissue. When the evaluation of complete response depends on this determination, it is recommended that the residual lesion be investigated (fine needle aspirate/biopsy) to confirm the complete response status.

Confirmation

The main goal of confirmation of objective response is to avoid overestimating the response rate observed. In cases where confirmation of response is not feasible, it should be made clear when reporting the outcome of such studies that the responses are not confirmed.

To be assigned a status of PR or CR, changes in tumor measurements must be confirmed by repeat assessments that should be performed no less than 4 weeks after the criteria for response are first met. Longer intervals as determined by the study protocol may also be appropriate.

In the case of SD, follow-up measurements must have met the SD criteria at least once after study entry at a minimum interval (in general, not less than 6-8 weeks) that is defined in the study protocol

Duration of overall response

The duration of overall response is measured from the time measurement criteria are met for CR or PR (whichever status is recorded first) until the first date that recurrence or PD is objectively documented, taking as reference for PD the smallest measurements recorded since the treatment started.

Duration of stable disease

SD is measured from the start of the treatment until the criteria for disease progression are met, taking as reference the smallest measurements recorded since the treatment started.

The clinical relevance of the duration of SD varies for different tumor types and grades. Therefore, it is highly recommended that the protocol specify the minimal time interval required between two measurements for determination of SD. This time interval should take into account the expected clinical benefit that such a status may bring to the population under study.

Response review

For trials where the response rate is the primary endpoint it is strongly recommended that all responses will be reviewed by an expert(s) independent of the study, at the study's completion. Simultaneous review of the patients' files and radiological images is the best approach.

Reporting of results

All patients included in the study must be assessed for response to treatment, even if

there are minor protocol treatment deviations. Each patient will be assigned one of the following categories: 1) complete response, 2) partial response, 3) stable disease, 4) progressive disease, 5) early death from malignant disease, 6) early death from toxicity, 7) early death because of other cause, or 8) unknown (not assessable, insufficient data).

All of the patients who met the eligibility criteria should be included in the main analysis of the response rate. Patients in response categories 4-8 should be considered as failing to respond to treatment (disease progression). Thus, an incorrect treatment schedule or drug administration does not result in exclusion from the analysis of the response rate. Precise definitions for categories 4-8 will be protocol specific.

All conclusions should be based on all eligible patients.

Subanalyses may then be performed on the basis of a subset of patients, excluding those for whom major protocol deviations have been identified (e.g., early death due to other reasons, early discontinuation of treatment, major protocol violations, etc.). However, these subanalyses may not serve as the basis for drawing conclusions concerning treatment efficacy, and the reasons for excluding patients from the analysis should be clearly reported.

The 95% confidence intervals should be provided.

PS TABLE

EASTERN COOPERATIVE ONCOLOGY GROUP PERFORMANCE STATUS ASSESSMENTS

**ECOG PERFORMANCE STATUS***	
Grade	ECOG
0	Fully active, able to carry on all pre-disease performance without restriction
1	Restricted in physically strenuous activity but ambulatory and able to carry out work of a light or sedentary nature, e.g., light house work, office work
2	Ambulatory and capable of all selfcare but unable to carry out any work activities. Up and about more than 50% of waking hours
3	Capable of only limited selfcare, confined to bed or chair more than 50% of waking hours
4	Completely disabled. Cannot carry on any selfcare. Totally confined to bed or chair
5	Dead

* Oken, M.M., Creech, R.H., Tormey, D.C., Horton, J., Davis, T.E., McFadden, E.T., Carbone, P.P.: Toxicity And Response Criteria Of The Eastern Cooperative Oncology Group. Am J Clin Oncol 5:649-655, 1982. Also see: http://www.ecog.org/general/perf_stat.html

B. WORLD MEDICAL ASSOCIATION DECLARATION OF HELSINKI

Recommendations guiding physicians in
biomedical research involving human subjects

Adopted by the 18th World Medical Assembly
Helsinki, Finland, June 1964

and amended by the
29th World Medical Assembly
Tokyo, Japan, October 1983

and the
41th World Medical Assembly
Hong Kong, September 1989

Introduction
It is the mission of the physician to safeguard the health of the people. His or her knowledge and conscience are dedicated to the fulfilment of this mission.
The Declaration of Geneva of the World Medical Association binds the physician with the words, «The health of my patient will be my first consideration», and the International Code of Medical Ethics declares that, «A physician shall act only in the patient's interest when providing medical care which might have the effect of weakening the physical and mental condition of the patient.»
The purpose of biomedical research involving human subjects must be to improve diagnostic, therapeutic and prophylactic procedures and the understanding of the aetiology and pathogenesis of disease.
In current medical practice most diagnostic, therapeutic or prophylactic procedures involve hazards. This applies especially to biomedical research.
Medical progress is based on research, which ultimately must rest in part on experimentation involving human subjects.
In the field of biomedical research a fundamental distinction must be recognised between medical research in which the aim is essentially diagnostic or therapeutic for a patient, and medical research, the essential object of which is purely scientific and without the implication of direct diagnostic or therapeutic value to the person subjected to the research.
Special caution must be exercised in the conduct of research, which may affect the environment and the welfare of animals used for the research must be respected.
Because it is essential that the results of laboratory experiments be applied to human beings to the further scientific knowledge and to help suffering humanity, the World Medical Association has prepared the following recommendations as a guide to every physician in biomedical research involving human subjects. This should be kept under review in the future. It must be stressed that the standards as drafted are only a guide to physicians all over the world. Physicians are not relieved from criminal, civil and ethical responsibilities under the laws of their countries.
I. Basic principles
Biomedical research involving human subjects must conform to generally accepted scientific principles and should be based on adequately performed laboratory and animal experimentation and on thorough knowledge of the scientific literature.
The design and performance of each experimental procedure involving human subjects should be clearly formulated in an experimental protocol which should be transmitted for consideration, comment and guidance to a special appointed committee independent of the investigator and the sponsor provided that this independent committee is in conformity with the laws and regulations of the country

in which the research experiment is performed.

Biomedical research involving human subjects should be conducted only by scientifically qualified persons and under the supervision of a clinically competent medical person. The responsibility for the human subject must always rest with a medically qualified person and never rest on the subject of the research, even through the subject has given his or her consent.

Biomedical research involving human projects cannot legitimately be carried out unless the importance of the objective is in proportion to the inherent risk to the subject.

Every biomedical research project involving human subjects should be preceded by careful assessment of predictable risks in comparison with foreseeable benefits to the subject or to others. Concern for the interests of the subject must always prevail over the interests of science and society.

The right of the research subject to safeguard his or her integrity must always be respected. Every precaution should be taken to respect the privacy of the subject and to minimize the impact of the study on the subject's physical and mental integrity and on the personality of the subject.

Physicians should abstain from engaging in research projects involving human subjects unless they are satisfied that the hazards involved are believed to be predictable. Physicians should cease any investigation if the hazards are found to outweigh the potential benefits.

In publication of the results of his or her research, the physician is obliged to preserve the accuracy of the results. Reports of experimentation not in accordance with the principles laid down in this Declaration should not be accepted for publication.

In any research of human beings, each potential subject must be adequately informed of the aims, methods, anticipated benefits and potential hazards of the study and the discomfort it may entail. He or she should be informed that he or she is at liberty to abstain from participation in the study and that he or she is free to withdraw his or her consent to participation at any time. The physician should then obtain the subject's freely-given informed consent, preferably in writing.

When obtaining informed consent for the research project the physician should be particularly cautious if the subject is in a dependent relationship to him or her or may consent under duress. In that case the informed consent should be obtained by a physician who is not engaged in the investigation and who is completely independent of this official relationship.

In case of legal incompetence, informed consent should be obtained from the legal guardian in accordance with national legislation. Where physical or mental incapacity makes it impossible to obtain informed consent, or when the subject is a minor, permission from the responsible relative replaces that of the subject in accordance with national legislation.

The research protocol should always contain a statement of the ethical considerations involved and should indicate that the principles enunciated in the present Declaration are complied with.

II. Medical research combined with professional care (Clinical research)

In the treatment of the sick person, the physician must be free to use a new diagnostic and therapeutic measure, if in his judgment it offers hope of saving life, re-establishing health or alleviating suffering.

The potential benefits, hazards and discomfort of a new method should be weighed against the advantages of the best current diagnostic and therapeutic methods.

In any medical study, every patient - including those of a control group, if any - should be assured of the best proven diagnostic and therapeutic method.

The refusal of the patient to participate in a study must never interfere with the physician-patient relationship.

If the physician considers it essential not to obtain informed consent, the specific

reasons for this proposal should be stated in the experimental protocol for transmission to the independent committee (1,2).

The physician can combine medical research with professional care, the objective being the acquisition of new medical knowledge, only to the extent that medical research is justified by its potential diagnostic or therapeutic value for the patient.

III. Non-therapeutic biomedical research involving human subjects (Non-clinical biomedical research)

In the purely scientific application of medical research carried out on a human being, it is the duty of the physician to remain the protector of the life and health of that person on whom biomedical research is being carried out.

The subjects should be volunteers - either healthy persons or patients for whom the experimental design is not related to patient’s illness.

The investigator or the investigating team should discontinue the research if in his/her or their judgment it may, if continued, be harmful to the individual.

In research on man, the interest of science and society should never take precedence over considerations related to the well-being of the subject**.**

C. PATIENT INFORMATION SHEET

Τυχαιοποιημένη μελέτη φάσης ΙΙ χορήγησης ιξαμπεπιλόνης εβδομαδιαία ή κάθε 3 εβδομάδες σε ασθενείς με μεταστατικό καρκίνο μαστού HER-2 αρνητικό, που έχουν λάβει ταξάνες σε προηγούμενη προ-εγχειρητική ή συμπληρωματική θεραπεία

Αγαπητ ....

Αυτό το ενημερωτικό σημείωμα έχει σχέση με ενδεχόμενη θεραπεία σας μέσω της συμμετοχής σας σε κλινική μελέτη [μελετήστε το παρακαλούμε προσεκτικά]

Σας ενημερώνουμε ότι στο πλαίσιο των θεραπευτικών δυνατοτήτων που μπορούμε να σας προσφέρουμε στην παρούσα φάση της ασθένειάς σας έχουμε την δυνατότητα, μετά από ενυπόγραφη συγκατάθεσή σας, να σας δώσουμε την ευκαιρία να συμμετάσχετε στην κλινική δοκιμή ενός νέου φαρμάκου [ιξαμπεπιλόνη] με δράση σε διάφορους συμπαγείς όγκους, με σκοπό να μελετήσουμε τη δράση του στη νόσο σας. Η συγκεκριμένη μελέτη διεξάγεται σε επιλεγμένα εξειδικευμένα κέντρα της χώρας μας. Παρόμοιες κλινικές δοκιμές του ιδίου φαρμάκου διεξάγονται και σε άλλες χώρες της Ευρώπης και την Αμερική. Η συμμετοχή σας είναι απολύτως εθελοντική. Πριν αποφασίσετε να συμμετάσχετε σε αυτή τη μελέτη, είναι σημαντικό να κατανοήσετε τι περιλαμβάνει η μελέτη αυτή. Παρακαλείστε να διαβάσετε προσεκτικά αυτές τις πληροφορίες και να υποβάλλετε οποιαδήποτε ερώτηση στον υπεύθυνο ιατρό.
Η μελετώμενη στο παρόν κλινικό πρωτόκολλο θεραπεία, αφορά στην ενδοφλέβια χορήγηση ενός νέου φαρμάκου [ιξαμπεπιλόνη] που αδρανοποιεί το μικροσωληναριακό δίκτυο του μηχανισμού της κυτταρικής διαίρεσης. Ο θεραπευτικά πλέον ωφέλιμος τρόπος χορήγησης του συγκεκριμένου φαρμάκου δεν έχει ακόμη καθορισθεί και για αυτό τον λόγο διεξάγεται αυτή η κλινική μελέτη. Περισσότερες από 2.500 ασθενείς έχουν λάβει και συνεχίζουν να λαμβάνουν θεραπεία με την ιξαμπεπιλόνη σε κλινικές μελέτες σε άλλες χώρες.
Περίπου 150 ασθενείς συνολικά αναμένεται να συμμετάσχουν στη μελέτη, σε περίπου 12 κέντρα στην Ελλάδα. Εάν ενταχθείτε σε αυτή τη μελέτη, η διάρκεια της συμμετοχής σας αναμένεται να είναι έως και 12 μήνες.
Η επιλογή του ρυθμού χορήγησης του φαρμάκου σε μία από τις παρακάτω θεραπείες γίνεται με τυχαίο ηλεκτρονικό τρόπο που δεν είναι προκαθορισμένος για τον κάθε συμμετέχοντα ασθενή

1. Θεραπεία Α: ενδοφλέβια χορήγηση ιξαμπεπιλόνης την $1^{η}$ ημέρα σε κύκλο 21 ημερών
2. Θεραπεία Β: ενδοφλέβια χορήγηση ιξαμπεπιλόνης την $1^{η}$, $8^{η}$ και $15^{η}$ ημέρα σε κύκλο 28 ημερών

Πριν από την έναρξη της θεραπείας χρειάζεται να μελετηθεί το προηγούμενο ιατρικό ιστορικό σας, να πραγματοποιηθεί μία φυσική εξέταση και να γίνει ένας κύκλος εργαστηριακών εξετάσεων και αξονική τομογραφία. Στις γυναίκες με δυνατότητα τεκνοποίησης, θα πραγματοποιηθεί ένα τεστ εγκυμοσύνης. Δεν θα πρέπει να είστε έγκυος ή σε λοχεία κατά την έναρξη της μελέτης και δεν θα πρέπει να μείνετε έγκυος κατά τη διάρκεια της μελέτης. Θα πρέπει να ενημερώσετε τον γιατρό σας άμεσα σε περίπτωση εγκυμοσύνης κατά τη διάρκεια λήψης του φαρμάκου της μελέτης.
Η θεραπεία χορηγείται σε εξωτερική βάση. Πριν από την έγχυση θα χορηγούνται φάρμακα για την πρόληψη μίας αλλεργικής αντίδρασης. Πριν από κάθε θεραπεία, θα

πραγματοποιείται μία φυσική εξέταση και αιματολογικές εξετάσεις. Εάν έχετε χαμηλές τιμές στις παραμέτρους των αιματολογικών εξετάσεων, ο γιατρός σας μπορεί να σας ζητήσει να κάνετε πιο συχνά αιματολογικές εξετάσεις, έως ότου αυτές επανέλθουν στα φυσιολογικά επίπεδα. Αξονικές τομογραφίες (CT) για την αξιολόγηση της νόσου σας θα πραγματοποιούνται κάθε 8 βδομάδες έως την ολοκλήρωση της θεραπείας σας. Η θεραπεία σε περίπτωση που τεκμηριώνεται επωφελής για σας χωρίς κάποια μη αποδεκτή τοξικότητα θα συνεχίζεται, αφού συζητηθεί με τον θεράποντα ιατρό σας μέχρι μέγιστου αριθμού 12 μηνών χορήγησης του φαρμάκου. Σε περίπτωση που η θεραπεία αποτύχει να σας βοηθήσει η θεραπευτική αντιμετώπιση θα συνεχισθεί με άλλους διαθέσιμους αντινεοπλασματικούς παράγοντες και φάρμακα καταστολής των συμπτωμάτων της νόσου. Θα σας ζητηθεί να επιστρέψετε στην κλινική κατά την ολοκλήρωση της μελέτης για μία φυσική εξέταση, μία αξιολόγηση της νόσου σας καθώς και των σημείων και συμπτωμάτων σας και αιματολογικές εξετάσεις.

Όπως και με όλα τα φάρμακα πού αναστέλλουν τον κυτταρικό πολλαπλασιασμό, κάποιες ανεπιθύμητες ενέργειες από την χορήγηση της θεραπείας είναι αναμενόμενες. Οι συνήθεις παρενέργειες είναι παροδική μείωση του αριθμού των κυκλοφορούντων στο αίμα λευκών αιμοσφαιρίων και αιμοπεταλίων, ναυτία ή/και έμετος, κοιλιακές κράμπες, διάρροια και δυσκοιλιότητα, που σε γενικές γραμμές μπορούν να ελεγθούν με χορήγηση υποστηρικτικών φαρμάκων. Μπορεί να εμφανιστούν αλλεργικές αντιδράσεις, που μπορεί να κυμαίνονται από ήπιο εξάνθημα έως, σπανιότερα, σοβαρές αντιδράσεις που σχετίζονται με δυσκολία στην αναπνοή και χαμηλή αρτηριακή πίεση. Θα σας δοθούν προληπτικά φάρμακα πριν την έγχυση της ιξαμπεπιλόνης, για να αποφευχθεί να συμβεί κάτι τέτοιο. Άλλες ανεπιθύμητες ενέργειες που ενδέχεται να εμφανιστούν περιλαμβάνουν απώλεια των μαλλιών, μούδιασμα και μυρμήγκιασμα των άκρων, απώλεια της όρεξης, πόνους των μυών και των αρθρώσεων, κόπωση ή αδυναμία, και άλλες σπανιότερες, όπως πυρετός, οίδημα (πρήξιμο), αλλοιώσεις των νυχιών, ακανόνιστος καρδιακός ρυθμός ή χαμηλή αρτηριακή πίεση. Σε περίπτωση συμμετοχής σας στη μελέτη θα σας ζητηθεί να αναφέρετε κάθε περίπτωση εμφάνισης δυσάρεστων παρενεργειών στον θεράποντα ιατρό κατά προτίμηση άμεσα. Επίσης είναι σημαντικό να αναφέρετε σε κάθε επίσκεψη σας, κατά προτίμηση γραμμένες, τις οποιεσδήποτε δυσάρεστες καταστάσεις είχατε μετά από κάθε θεραπεία. Θα καταβάλλεται κάθε δυνατή προσπάθεια, ώστε να προληφθεί η εμφάνιση των παρενεργειών αυτών και στην περίπτωση που εμφανιστούν, θα αντιμετωπίζονται άμεσα.

Επιπλέον, θα θέλαμε εφόσον συμμετέχετε στη μελέτη να δώσετε τη συγκατάθεσή σας για να χρησιμοποιήσουμε βιολογικό σας υλικό (αίμα, υλικό από βιοψία), για ερευνητικούς σκοπούς. Είναι δεδομένο, ότι τα προσωπικά σας στοιχεία από τις μελέτες αυτές θα τύχουν απόλυτης προστασίας (δεν θα ανακοινωθούν). Ο σκοπός της λήψης των δειγμάτων βιολογικού σας υλικού είναι καθαρά ερευνητικός και τα δείγματα δεν θα χρησιμοποιηθούν για γενετικό έλεγχο. Όλα τα δείγματα θα καταστραφούν μετά από ένα μέγιστο διάστημα 10 ετών. Τα αποτελέσματα της έρευνας από το δικό σας βιολογικό υλικό θα γίνονται γνωστά στον θεράποντα ιατρό σας, ενώ τα γενικά αποτελέσματα της μελέτης θα δημοσιευτούν μετά την ολοκλήρωση της μελέτης. Τα δείγματα όγκου που θα συλλεγούν στη μελέτη, τα αποτελέσματα της μεταφραστικής έρευνας, οποιαδήποτε πατέντα, διαγνωστικό τεστ, φάρμακο και βιολογικό προιόν που θα αναπτυχθεί άμεσα ή έμμεσα σαν αποτέλεσμα αυτής της μελέτης, καθώς και κάθε πληροφορία που θα προέλθει άμεσα ή έμμεσα από αυτά τα δείγματα, είναι όλα περιουσία της Ελληνικής Συνεργαζόμενης Ογκολογικής Ομάδας (ΕΣΟΟ), και με την υπογραφή σας συμφωνείτε οτι δεν έχετε κανένα δικαίωμα σε αυτά.

Πληροφορίες σχετικά με άλλες θεραπείες που είναι διαθέσιμες για την πάθησή σας μπορούν να σας δοθούν από τον ιατρό σας. Εάν δεν επιλέξετε να συνεχίσετε σε αυτή τη μελέτη, θα συζητηθούν λεπτομερώς μαζί σας οι εναλλακτικές θεραπείες που είναι διαθέσιμες για τη νόσο σας, καθώς και τα πιθανά οφέλη τους.

Δεν θα υπάρξει καμία χρέωσή σας για το φάρμακο της μελέτης ιξαμπεπιλόνη. Το

φάρμακο θα χορηγηθεί δωρεάν από την παρασκευάστρια εταιρεία (Bristol-Myers Squibb).
Ο χορηγός έχει φροντίσει για την ύπαρξη ασφάλισης που σας παρέχει κάλυψη σε περίπτωση οποιασδήποτε βλάβης της υγείας σας.

- Ο χορηγός ευθύνεται για κάθε άμεση ή έμμεση ζημία που θα προκληθεί στο συμμετέχοντα από τη χορήγηση του φαρμάκου ή από οποιαδήποτε κλινική παρέμβαση ή διαδικασία στο πλαίσιο της συμμετοχής του στη μελέτη η οποία δεν θα είχε πραγματοποιηθεί, αν ο συμμετέχων δεν είχε λάβει μέρος στη μελέτη.
- Για κάθε αξίωση του συμμετέχοντα στην πιο πάνω κλινική δοκιμή κατά οποιουδήποτε υπευθύνου αρμόδια είναι τα ελληνικά δικαστήρια.
- Ο συμμετέχων στη μελέτη εκχωρεί από τώρα τις αξιώσεις του κατά των κατά τα ανωτέρω υπευθύνων, στον ασφαλιστικό του οργανισμό, αν αυτός ο ασφαλιστικός οργανισμός επιβαρυνθεί οικονομικά από τη συμμετοχή του ασφαλισμένου στην πιο πάνω μελέτη (ενδεικτικά και όχι αποκλειστικά αν ο ασφαλιστικός οργανισμός επιβαρυνθεί με εξετάσεις για την κλινική μελέτη που δεν θα γίνονται αλλιώς, την αξία του χορηγούμενου φαρμάκου, τη νοσηλεία από επιπλοκές εξαιτίας της κλινικής δοκιμής, κλπ.).

Οπωσδήποτε, μπορείτε όποτε ζητήσετε, να αποσύρετε τη συγκατάθεσή σας για τη συμμετοχή στη μελέτη και να αποσυρθείτε από αυτήν. Στην περίπτωση αυτή θα φροντίσουμε να έχετε την καλύτερη θεραπεία και περίθαλψη ανάλογα με την εξέλιξη της ασθένειάς σας.

Για οποιαδήποτε περαιτέρω πληροφορία σχετικά με τη μελέτη, παρακαλούμε επικοινωνείστε με:

Ονοματεπώνυμο ερευνητή: **Τηλ. No.**

ΣΥΓΚΑΤΑΘΕΣΗ ΑΣΘΕΝΟΥΣ

Σας παρακαλούμε να διαβάσετε προσεκτικά την παρακάτω ενότητα και, εάν συμφωνείτε, να υπογράψετε και να σημειώσετε ιδιοχείρως την ημερομηνία στο κάτω μέρος της σελίδας.

Δηλώνω ενυπόγραφα,
ότι έχω ενημερωθεί από τον/την Ιατρό
....………….
για την κλινική θεραπευτική δοκιμή με το φάρμακο ιξαμπεπιλόνη, έχω διαβάσει και κατανοώ τις πληροφορίες που παρουσιάζονται σε αυτό το Έντυπο Συγκατάθεσης μετά από Ενημέρωση και αποδέχομαι την εθελοντική συμμετοχή μου σε αυτή τη μελέτη.
Αποδέχομαι επίσης τις σύμφωνα με το εγκεκριμένο θεραπευτικό πρωτόκολλο επιπλέον εξετάσεις και δειγματοληψίες καθώς και την προσφορά βιολογικού μου υλικού (αίμα, υλικό βιοψίας).
Ο/η Ιατρός με ενημέρωσε για τις ενδεχόμενες παρενέργειες της θεραπείας και απάντησε σε όλες τις σχετικές με την πάθηση και την συγκεκριμένη θεραπεία που μου προτείνει ερωτήσεις μου.
Γνωρίζω ότι είμαι ελεύθερος/η να αρνηθώ την συμμετοχή μου και ότι μπορώ να αποσύρω την συγκατάθεση μου ανά πάσα στιγμή κατά τη διάρκεια της μελέτης. Γνωρίζω οτι δεν θα χάσω κανένα από τα δικαιώματα που έχω στο πλαίσιο της τοπικής νομοθεσίας υπογράφοντας και χρονολογώντας αυτό το έντυπο. Τέλος, γνωρίζω οτι θα λάβω ένα υπογεγραμμένο και χρονολογημένο αντίγραφο αυτού του Εντύπου Συγκατάθεσης.

Όνομα ασθενούς :...

Υπογραφή ασθενούς: ....

Ημερομηνία.............................

Υπογραφή μάρτυρος *που παρέστη στην προφορική συγκατάθεση της ασθενούς*

(αν υπήρχε):... Ημερομηνία :

Υπογραφή ερευνητή: .... Ημερομηνία :

D. NCI COMMON TOXICITY CRITERIA VERSION 3

[to be attached**]**

Adverse Events file
