# Supplementary material for: Ixabepilone Administered Weekly or Every Three Weeks in HER2-Negative Metastatic Breast Cancer Patients; A Randomized Non-Comparative Phase II Trial
Source: PLoS One. 2013 Jul 23;8(7):e69256. doi: 10.1371/journal.pone.0069256 (PMC3720651; doi:10.1371/journal.pone.0069256)
Supplement: Supporting Information S1 — Detailed eligibility criteria and treatment dose modifications. (DOC) [file pone.0069256.s012.doc]

***Detailed eligibility criteria***

Patients of child-bearing potential should have had a negative serum or urine pregnancy test within 72 hours before randomization. Patients had to be fully recovered from all prior treatment-related events. Patients with a) grade ≥2 peripheral neuropathy according to the National Cancer Institute Common Terminology Criteria (NCI-CTC) Version 2.0, b) psychiatric disorders or other medical conditions rendering the patient incapable to comply with protocol requirements, c) history or evidence of CNS metastases, d) clinically significant cardiac disease (e.g. unstable angina, congestive heart failure or myocardial infarction) within six months prior to randomization, e) prior severe hypersensitivity reactions to agents containing Cremophor EL and f) concurrent active malignancies, except of completely resected non-melanoma or in situ cervical tumors were excluded from the study. Because of concerns that ixabepilone metabolism may be inhibited by potent cytochrome P450 3A4 inhibitors, patients had to have stopped treatment with these medications at least 72 hours before randomization.

### *Re-treatment criteria and dose modifications for the 3-weekly dosing arm (Group A)*

***Re-treatment criteria***

Patients did not begin a new cycle of treatment unless the **neutrophil** count was at least 1,500 cells/mm^3^ and the **platelet** count was at least 100,000 cells/mm^3^ and non-hematological toxicities had improved.

***Dose modifications***

Dose reductions were implemented based on non-hematological toxicity or blood counts according to the following table. If toxicities recurred after the initial dose reduction, an additional 20% dose reduction was recommended. If toxicities recurred after the second dose reduction, ixabepilone had to be discontinued.

| Dose adjustments for toxicities. | |
| --- | --- |
| Toxicity | Suggested Dose Modification |
| Non-hematological |  |
| Grade 2 neuropathy (moderate) lasting ≥7 days | Decrease the dose by 20% |
| Grade 3 neuropathy (severe) lasting <7 days | Decrease the dose by 20% |
| Grade 3 neuropathy (severe) lasting ≥7 days or disabling neuropathy | Discontinue treatment |
| Any grade 3 toxicity (severe) other than neuropathy or transient grade 3 arthralgia/myalgia and fatigue | Decrease the dose by 20% |
| Any grade 4 toxicity (disabling) | Discontinue treatment |
| **Hematological** |  |
| Neutrophils <500 cells/mm^3^ for ≥7 days  Febrile neutropenia  Platelets <25,000/mm^3^ or platelets <50,000/mm^3^ with bleeding | Decrease the dose by 20%  Decrease the dose by 20%  Decrease the dose by 20% |

### *Re-treatment criteria and dose modifications for the weekly dosing arm (Group B)*

***Re-treatment criteria***

In Group B, weekly dosing re-treatment **within a cycle** was allowed **at a reduced dose,** if ANC was between 1000/mm^3^ and 1499/mm^3^ and/or platelets between 75,000/mm^3^ and 99,999/mm^3^ and treatment related non-hematological toxicities were ≤grade 2 except for grade 2 neuropathy. Subjects with **ongoing ≥grade 2 neuropathy** were retreated. Patients with ≤grade 2 neuropathy who previously experienced grade 2 neuropathy lasting <7 days were re-treated at the same dose. Patients with prior grade 2 neuropathy lasting ≥7 days require dose reduction. Missed doses within a cycle were made-up and did not influence the duration of the treatment cycle.

### *Dose delays*

Toxicities (including neurotoxicity) required resolution to grade 1 or to baseline before the next cycle of treatment was administered. If patients were not allowed to be treated within 2 weeks of intended dosing, due to toxicity, they had to be taken off treatment.

| Dose modifications for weekly re-treatment (intra-cycle) in Group B (weekly ixabepilone). | | | | | |
| --- | --- | --- | --- | --- | --- |
| Hematological toxicity | | | | | |
| Neutrophils  (per mm^3^) |  | Platelets  (per mm^3^) |  | Neurotoxicity | Action |
| ≥1500 | **AND** | ≥100,000 | **AND** | <Gr 2 or Gr 2 <7 days resolved to <Gr 2 | Maintain dose |
| 1000-1499 | **AND/OR** | 75,000-99,999 | **AND/OR** | Gr 2 ≥7 days resolved to <Gr 2 | Decrease dose by one level |

^a^If treatment was held, CBC was repeated until neutrophils were ≥1500/mm^3^ and platelets were ≥100,000/mm^3^.

### *Treatment discontinuation*

Patients had to be removed from the study for the following reasons:

- Documented disease progression
- Persistent grade 2 neuropathy
- Grade 3 neuropathy lasting more than 7 days
- Grade 3 or 4 toxicities requiring more than two dose reductions, or delay of treatment for more than 5 weeks in group A or 6 weeks in group B from the beginning of the last cycle.
