# Supplementary material for: Ixabepilone Administered Weekly or Every Three Weeks in HER2-Negative Metastatic Breast Cancer Patients; A Randomized Non-Comparative Phase II Trial
Source: PLoS One. 2013 Jul 23;8(7):e69256. doi: 10.1371/journal.pone.0069256 (PMC3720651; doi:10.1371/journal.pone.0069256)
Supplement: Supporting Information S2 — Detailed methodology for the assessment of translational biomarkers. (DOC) [file pone.0069256.s013.doc]

***Immunohistochemistry (IHC)*** was employed on serial 2.5 μm thick sections from the original blocks or the tissue microarray (TMA) blocks at the Laboratory of Molecular Oncology of the Hellenic Foundation of Cancer Research, Aristotle University of Thessaloniki School of Medicine. The tissues were mounted on adhesive microscope slides and subjected to IHC using Bond Max III™ (Leica Microsystems, Wetzlar, Germany) and i6000 (Biogenex, San Ramon, CA) autostainers. The methods employed for the tested markers, i.e., estrogen receptor alpha (ER), progesterone receptor (PgR), HER2, Ki67, βIII-tubulin, γ-tubulin, tau protein, topoisomerase II alpha (TopoIIa), MLH-1, MSH-2, MSH-6 and PMS-2 are shown in Table 1. The evaluation of all IHC sections was performed by experienced pathologists, blinded as to the patients’ clinical characteristics and survival data, according to previously proposed or established criteria (presented in Table 1). With respect to mismatch repair (MMR) proteins, tumors were classified as microsatellite instability-high (MSI-H) in case of no expression of two or more MMR proteins, MSI-low (MSI-L) in case of no expression of one protein and MSI-stable (MSS) in case of expression of all four proteins .

***Fluorescence in situ hybridization (FISH)***. The *HER2* gene was considered to be amplified when the ratio of the gene probe/centromere probe was ≥2.2 , or the *HER2* copy number was >6 . HER2 was considered to be positive if the gene was amplified (ratio >2.2 or copy number >6) by FISH and/or a HER2 score of 3+ was obtained by IHC. The TOP2A gene was considered to be amplified when the ratio of the respective gene probe/centromere probe was ≥2.0 and deleted when the ratio was <0.8 .

***Single Nucleotide Polymorphism (SNP) assessment***. DNA was extracted from peripheral blood buffy coats by using the QIAamp DNA Blood Mini Kit (Qiagen, Hilden, Germany). Since no SNPs specifically associated with epothilone toxicity have been reported, we investigated taxane-specific SNPs that were registered in the Pharmacogenomics database ([http://www.pharmgkb.org](http://www.pharmgkb.org/)). Sequencing assays were designed for: rs2032582 (c.2677T/G/A, p.S893T/A, also including ABCB1 c.2734C/T), rs1045642 (c.3435T>C, p.I1145I) and rs1128503 (c.1236T>C, p.G412G) in the ABCB1 gene; rs12721627 (c.20716C/G, p.T185S) in the CYP3A4 gene; and, rs11572080 (c.7225G/A, p.R69K, also including c.2077A/G and the intronic T-ins) in the CYP2C8 gene. SNP sequencing assays are presented in Table 2. dd-sequencing was performed with the BigDye® Terminator v1.1 Cycle Sequencing Kit on PCR products coupled with M13 primers and results were visualized in an ABI3130XL genetic analyzer (all from Applied Biosystems/Life Technologies).

***mRNA expression studies.*** Tumor tissue RNA was extracted from TMA cores (6 sections, 10μm thick) upon deparaffinization and overnight tissue lysis with proteinase K at 56^o^C with TRIZOL-LS and reverse transcribed with random primers and the Superscript III system according to the manufacturer’s instructions (Invitrogen, Life Technologies, Paisley, UK). Upon UV measurements, molecular samples were normalized at 25 ng template/μl and kept at -20^o^C until use. A commercially available reference RNA (TaqMan® Control Total RNA, cat. no 4307281, Applied Biosystems/Life Technologies) was used as a positive control in each run. As an endogenous control and for the normalization of CT (cycle threshold) values, an assay targeting GUSB mRNA (beta-glucuronidase [#4333767F]) was used . The assays for the assessment of ABCB1, CYP3A4, CYP2C8, MAPT, TUBB3, as well as for the endogenous reference GUSB transcript, are presented in detail in Table 3. ABCB1, CYP2C8 and CYP3A4 mRNA expression was considered in comparison with the respective germline SNPs; MAPT and TUBB3 mRNA expression with the respective proteins that were examined by IHC. Reactions were run in duplicates in an ABI7900HT real time PCR system. Relative quantification (RQ) was assessed in a linear mode as (40 – dCT) , whereby dCT = (avg CT target) – (avg CT GUSB). PCR assay stability was evaluated among runs with the reference RNA, revealing acceptable inter-run deltaRQ variability for the five assays (ABCB1 deltaRQ=0.33; CYP2C8 deltaRQ=0.69; CYP3A4 deltaRQ=0.47; MAPT deltaRQ=0.14; and TUBB3 deltaRQ=0.27). Exclusion criteria for RQ analysis were GUSB CT values higher than 36 for each duplicate; and, deltaRQ values higher than 0.85 per duplicate pair.

**Table 1.** Proteins, source and dilution of antibodies, staining procedures and patterns and interpretation analysis.

| Protein | Antibody clone (source) | Antibody dilution | Pretreatment (t/ER) | Incubation Time | IHC Staining Detection System | Scoring System | Cut-off (%, I) | Staining pattern | Reference |
| --- | --- | --- | --- | --- | --- | --- | --- | --- | --- |
| ER | 6F11 (1) | 1:60 | 20'/ER2 | 30' | Bond Polymer + DAB | Allred score | 1% | N |  |
| PgR | 1A6 (1) | 1:60 | 20'/ER2 | 30' | Bond Polymer + DAB | Allred score | 1% | N |  |
| HER2 | PL (2) | 1:250 | 25'/ER1 | 30' | Bond Polymer + DAB | 0-3+^1^ | 30% | M |  |
| Ki67 | MIB1 (2) | 1:70 | 15'/ER2 | 20' | Bond Polymer + DAB | SQ | 14%* | N |  |
| βIII-tubulin | MU177-UC (3) | 1:1250 | 15'/ER1 | 20' | Bond Polymer + DAB | SQ | I ≥ 2 | C |  |
| γ-tubulin | C-11 (4) | 1:300 | 20'/ER2 | 30' | Bond Polymer + DAB | SQ | I > 2 | C |  |
| Tau protein | T1029 (5) | 1:100 | 20'/ER1 | O/n | Envision + DAB | SQ | I ≥ 2 | C |  |
| TopoIIa | KiS1 (2) | 1:200 | 15'/ER2 | 30' | Envision + DAB | SQ | 5%, I ≥ 2 | N |  |
| MLH-1 | ES05 (1) | 1:80 | 30'/ER1 | 30' | Bond Polymer + DAB | SQ | Any+ | N |  |
| MSH-2 | 25D12 (1) | 1:40 | 20'/ER1 | 30' | Bond Polymer + DAB | SQ | Any+ | N |  |
| MSH-6 | PU29 (1) | 1:100 | 20'/ER1 | 30' | Bond Polymer + DAB | SQ | Any+ | N |  |
| PMS-2 | M0R4G (1) | 1:50 | 30'/ER2 | 30' | Bond Polymer + DAB | SQ | Any+ | N |  |
| C: Cytoplasm; DAB: 3,3'-Diaminobenzidine; ER: Epitope retrieval; ER1: Citric acid, pH: 6.0; ER2: Ethylenediaminetetraacetate, pH: 8.8; I: Intensity; M: Membranous; N: Nuclear; O/n: Overnight; SQ: Semi-quantitative; t: Time. | | | | | | | | | |
| 1: Leica Biosystems, Newcastle, UK; 2: Dako, Glostrup, Denmark; 3: Biogenex, San Ramon, CA; 4: Santa Cruz, Santa Cruz, CA; 5: United States Biological, Swampscott, MA.  *Proliferation Index was evaluated as Low if <14% and High if ≥14%; ^1^score 3+ in >30% of tumor cells was considered to be positive. | | | | | | | | | |

**Table 2: SNP sequencing assays**

| **SNP ID** | **Gene** | **Main SNP in the area** | **Ancestral** | **Aminoacid change** | **Primer orientation** | **Primer sequence (5' - 3')*** | **Amplicon length** |
| --- | --- | --- | --- | --- | --- | --- | --- |
| rs2032582 | ABCB1 | c. 2677T/G/A | G | p. S893T, S893A | forward | TTCATCTATGGTTGGCAACT | 316bp |
|  |  |  |  |  | reverse | AGAACTGGCTTTGCTACTTTC |  |
| rs1045642 | ABCB1 | c. 3435T/C | C | p. I1145I | forward | TGCTGGTCCTGAAGTTGATCT | 292bp |
|  |  |  |  |  | reverse | GAGGCTGCCACATGCTC |  |
| rs1128503 | ABCB1 | c. 1236T/C | C | p. G412G | forward | CATCTCGAAAAGAAGTTAAGG | 340bp |
|  |  |  |  |  | reverse | CTAGCTCGCATGGGTC |  |
| rs12721627 | CYP3A4 | c. 20716C/G | C | p. T185S | forward | GGGTTTCCTGTTGCAT | 318bp |
|  |  |  |  |  | reverse | TAAGTGGATGAATTACATGGT |  |
| rs11572080 | CYP2C8 | c. 7225G/A | G | p. R69K | forward | TGGAAAACAGAGCCCTAA | 306bp |
|  |  |  |  |  | reverse | GTTAAGGTCAATGACGCAGAG |  |

*: All primer sequences were coupled with M13 primers, M13-forward GTAAAACGACGGCCAGT and M13-reverse CAGGAAACAGCTATGACC; PCR annealing temperature was universally set at 68^o^ C (two-step PCR).

**Table 3: TaqMan® Gene Expression Assays (FAM - MGB) used in this study.**

| **Assay ID** | **Gene symbol** | **Other aliases** | **Gene name** | **Genbank reference** | **Amplicon size** | **Exon spanning** | **Assay location** |
| --- | --- | --- | --- | --- | --- | --- | --- |
| Hs00184491_m1 | ABCB1 | ABC20, CD243, CLCS, GP170, MDR1, MGC163296, P-GP, PGY1 | ATP-binding cassette, sub-family B (MDR/TAP), member 1 | NM_000927.4 | 110 | 25 - 26 | 3283 |
| Hs00946140_g1 | CYP2C8 | CPC8, CYPIIC8, MP-12/MP-20 | Cytochrome P450, family 2, subfamily C, polypeptide 8 | NM_000770.3, NM_001198853.1, NM_001198855.1 | 80 | 2 - 3, 2 - 3, 3 - 4 | 425, 463, 525 |
| Hs00604506_m1 | CYP3A4 | CP33, CP34, CYP3A, CYP3A3, CYPIIIA3, CYPIIIA4, HLP, MGC126680, NF-25, P450C3, P450PCN1 | Cytochrome P450, family 3, subfamily A, polypeptide 4 | NM_001202855.2, NM_017460.5 | 119 | 2 - 3 | 267 |
| Hs00902194_m1 | MAPT | DDPAC, FLJ31424, FTDP-17, MAPTL, MGC138549, MSTD, MTBT1, MTBT2, PPND, TAU | Microtubule-associated protein tau | NM_001123066.3, NM_001123067.3, NM_001203251.1, NM_001203252.1, NM_005910.5, NM_016834.4, NM_016835.4, NM_016841.4 | 59 | 14 - 15, 10 - 11, 9 - 10, 10 - 11, 11 - 12, 9 - 10, 13 - 14, 8 - 9 | 2442, 1350, 1257, 1344, 1437, 1263, 2388, 1170 |
| Hs00964963_g1 | TUBB3 | CDCBM, CFEOM3A, TUBB4, beta-4 | Tubulin, beta 3 | NM_001197181.1, NM_006086.3 | 67 | 3 - 4 | 455, 398 |
| 4333767F | GUSB |  | Beta glucuronidase | NM_000181.1 | 81 | 11 - 12 |  |

**References**

1. Murata H, Khattar NH, Kang Y, Gu L, Li GM (2002) Genetic and epigenetic modification of mismatch repair genes hMSH2 and hMLH1 in sporadic breast cancer with microsatellite instability. Oncogene 21: 5696-5703.

2. Wolff AC, Hammond ME, Schwartz JN, Hagerty KL, Allred DC, et al. (2007) American Society of Clinical Oncology/College of American Pathologists guideline recommendations for human epidermal growth factor receptor 2 testing in breast cancer. J Clin Oncol 25: 118-145.

3. Vanden Bempt I, Van Loo P, Drijkoningen M, Neven P, Smeets A, et al. (2008) Polysomy 17 in breast cancer: clinicopathologic significance and impact on HER-2 testing. J Clin Oncol 26: 4869-4874.

4. Knoop AS, Knudsen H, Balslev E, Rasmussen BB, Overgaard J, et al. (2005) retrospective analysis of topoisomerase IIa amplifications and deletions as predictive markers in primary breast cancer patients randomly assigned to cyclophosphamide, methotrexate, and fluorouracil or cyclophosphamide, epirubicin, and fluorouracil: Danish Breast Cancer Cooperative Group. J Clin Oncol 23: 7483-7490.

5. Sanchez-Navarro I, Gamez-Pozo A, Gonzalez-Baron M, Pinto-Marin A, Hardisson D, et al. (2010) Comparison of gene expression profiling by reverse transcription quantitative PCR between fresh frozen and formalin-fixed, paraffin-embedded breast cancer tissues. Biotechniques 48: 389-397.

6. Hennig G, Gehrmann M, Stropp U, Brauch H, Fritz P, et al. (2010) Automated extraction of DNA and RNA from a single formalin-fixed paraffin-embedded tissue section for analysis of both single-nucleotide polymorphisms and mRNA expression. Clin Chem 56: 1845-1853.

7. Hammond ME, Hayes DF, Dowsett M, Allred DC, Hagerty KL, et al. (2010) American Society of Clinical Oncology/College Of American Pathologists guideline recommendations for immunohistochemical testing of estrogen and progesterone receptors in breast cancer. J Clin Oncol 28: 2784-2795.

8. Cheang MC, Chia SK, Voduc D, Gao D, Leung S, et al. (2009) Ki67 index, HER2 status, and prognosis of patients with luminal B breast cancer. J Natl Cancer Inst 101: 736-750.

9. Pusztai L, Jeong JH, Gong Y, Ross JS, Kim C, et al. (2009) Evaluation of microtubule-associated protein-Tau expression as a prognostic and predictive marker in the NSABP-B 28 randomized clinical trial. J Clin Oncol 27: 4287-4292.

10. Fountzilas G, Kourea HP, Bobos M, Televantou D, Kotoula V, et al. (2011) Paclitaxel and bevacizumab as first line combined treatment in patients with metastatic breast cancer: the Hellenic Cooperative Oncology Group experience with biological marker evaluation. Anticancer Res 31: 3007-3018.

11. Bhargava R, Lal P, Chen B (2005) HER-2/neu and topoisomerase IIa gene amplification and protein expression in invasive breast carcinomas: chromogenic in situ hybridization and immunohistochemical analyses. Am J Clin Pathol 123: 889-895.

12. Hatch SB, Lightfoot HM, Jr., Garwacki CP, Moore DT, Calvo BF, et al. (2005) Microsatellite instability testing in colorectal carcinoma: choice of markers affects sensitivity of detection of mismatch repair-deficient tumors. Clin Cancer Res 11: 2180-2187.
